# Viral Perturbations of Host Networks Reflect Disease Etiology

**DOI:** 10.1371/journal.pcbi.1002531

**Published:** 2012-06-28

**Authors:** Natali Gulbahce, Han Yan, Amélie Dricot, Megha Padi, Danielle Byrdsong, Rachel Franchi, Deok-Sun Lee, Orit Rozenblatt-Rosen, Jessica C. Mar, Michael A. Calderwood, Amy Baldwin, Bo Zhao, Balaji Santhanam, Pascal Braun, Nicolas Simonis, Kyung-Won Huh, Karin Hellner, Miranda Grace, Alyce Chen, Renee Rubio, Jarrod A. Marto, Nicholas A. Christakis, Elliott Kieff, Frederick P. Roth, Jennifer Roecklein-Canfield, James A. DeCaprio, Michael E. Cusick, John Quackenbush, David E. Hill, Karl Münger, Marc Vidal, Albert-László Barabási

**Affiliations:** 1 Center for Complex Networks Research (CCNR) and Department of Physics, Northeastern University, Boston, Massachusetts, United States of America; 2 Center for Cancer Systems Biology (CCSB) and Department of Cancer Biology, Dana-Farber Cancer Institute, Boston, Massachusetts, United States of America; 3 Department of Genetics, Harvard Medical School, Boston, Massachusetts, United States of America; 4 Center for Cancer Computational Biology (CCCB), Department of Biostatistics and Computational Biology and Department of Cancer Biology, Dana-Farber Cancer Institute and Department of Biostatistics, Harvard School of Public Health, Boston, Massachusetts, United States of America; 5 Department of Chemistry, Simmons College, Boston, Massachusetts, United States of America; 6 Department of Natural Medical Sciences and Department of Physics, Inha University, Incheon, Korea; 7 Department of Medical Oncology, Dana-Farber Cancer Institute, and Department of Medicine, Brigham and Women's Hospital and Harvard Medical School, Boston, Massachusetts, United States of America; 8 Infectious Diseases Division, The Channing Laboratory, Brigham and Women's Hospital and Department of Medicine, Harvard Medical School, Boston, Massachusetts, United States of America; 9 Blais Proteomics Center and Department of Cancer Biology, Dana-Farber Cancer Institute, Boston, Massachusetts, United States of America; 10 Department of Health Care Policy, Harvard Medical School and Department of Sociology, Harvard University, Cambridge, Massachusetts, United States of America; 11 Department of Biological Chemistry and Molecular Pharmacology, Harvard Medical School, Boston, Massachusetts, United States of America; 12 Department of Medicine, Brigham and Women's Hospital, Harvard Medical School, Boston, Massachusetts, United States of America; University of Washington, United States of America

## Abstract

Many human diseases, arising from mutations of disease susceptibility genes (genetic diseases), are also associated with viral infections (virally implicated diseases), either in a directly causal manner or by indirect associations. Here we examine whether viral perturbations of host interactome may underlie such virally implicated disease relationships. Using as models two different human viruses, Epstein-Barr virus (EBV) and human papillomavirus (HPV), we find that host targets of viral proteins reside in network proximity to products of disease susceptibility genes. Expression changes in virally implicated disease tissues and comorbidity patterns cluster significantly in the network vicinity of viral targets. The topological proximity found between cellular targets of viral proteins and disease genes was exploited to uncover a novel pathway linking HPV to Fanconi anemia.

## Author Summary

Many “virally implicated human diseases” - diseases for which there is scientific consensus of viral involvement - are associated with genetic alterations in particular disease susceptibility genes. We proposed and demonstrated that for two human viruses, Epstein-Barr virus and human papillomavirus, topological proximity should exist between host targets of viruses and genes associated with virally implicated diseases on host interactome networks (local impact hypothesis). For representative EBV- and HPV16- implicated diseases, genes in the neighborhood of viral targets in the host interactome have significantly shifted expression levels in virally implicated disease tissues, in line with the local impact hypothesis. The viral neighborhoods in the host interactome, along with their disease associations, defined as “viral disease networks”, contain connections known to be informative upon disease mechanisms as well as diseases whose associations with viruses are not yet known. We prioritized these diseases for their candidacy as potential virally implicated diseases based on network topology, and benchmarked this prioritization of candidate diseases using relative risk measurement which depicts population-based clinical associations between candidate diseases and viral infection. Exogenous expression of HPV viral proteins in a human cell line offered evidence for a novel disease pathway that links HPV to Fanconi anemia.

## Introduction

Functional interactions between cellular targets of viral proteins and disease susceptibility genes [1], [2], [3], [4], [5] might play key roles in disease etiology. Advances in the mapping of the human interactome network, as well as in the systematic identification of gene-disease associations, provide functional data that can be used to explore fundamental connections between viral targets and disease genes. Here we formulate a *local impact* hypothesis, stating that diseases that can be either genetic or virally implicated can be better understood from a network perspective [6]. By this hypothesis the products of disease susceptibility genes should reside in the network vicinity of the corresponding viral targets [7], [8].

To test this hypothesis we focused on Epstein-Barr virus (EBV) and human papillomavirus (HPV) type 16, two human viruses that differ in their host tropism, genome and proteome size, and disease etiology. We find that the disease susceptibility genes of known virally implicated diseases are in the immediate network vicinity of the host proteins that are targeted by these viruses. We could identify a viral disease module for EBV and HPV, representing a subnetwork of the interactome that contains key mechanistic pathways responsible for the observed virus-disease associations. A computational prioritization procedure, joined by large-scale comorbidity and expression pattern analyses, identified new potential mechanistic disease pathways. To validate several of these pathways, HPV16 E6 and E7 oncogenes were independently expressed in primary human fibroblast (IMR90) and keratinocyte (HFK) cell populations to identify disease-associated genes whose expression levels were significantly altered in these E6/E7-expressing cell populations. We could identify a novel pathway that links HPV to a specific form of Fanconi Anemia. The systematic network-based framework we applied works to decipher the interplay between viruses and disease phenotypes.

## Results

### Virally implicated diseases and interactome construction

We define as “virally implicated diseases” those diseases whose association with a particular virus is supported by peer-reviewed publications in the literature. This list includes not only diseases for which there is universally accepted consensus that a virus is causal (such as cervical cancer for HPV16 and Burkitt's lymphoma for EBV), but also diseases which have some reproducible evidence of viral association but for which the mechanistic pathways are not worked out. There is significant and legitimate controversy and subjectivity regarding which diseases are virus-associated or virally implicated, so to avoid infusing personal bias in the selection process, we turned to several recently published authoritative review articles [1], [2], [9], [10] as well as additional literature searches. From these sources we compiled a list of 17 and 14 diseases for which a disease etiology with EBV and HPV16 has been claimed.

Most of the selected virally implicated diseases (13 for EBV and 9 for HPV16) are genetic diseases in that they have been associated with mutations in at least one human gene (Table 1), as compiled in the Online Mendelian Inheritance in Man (OMIM) Morbid Map repository [11], although there are notable exceptions. Infectious mononucleosis, a disease clearly linked to EBV infection, lacks any known susceptibility genes (Table 1a). Similarly, cervical carcinoma, known to be caused by HPV infections, does not have a known genetic association (Text S1). Whenever a given disease is universally associated with viral infection and not driven by genetic changes, our approach will not yield a link between these diseases and the corresponding virus.

**Table 1:** Virally implicated diseases.

Table 1A		
EBV-implicated diseases	Mapped genes	ICD-9 code(s)
1. B cell lymphomas incl. Burkitt's lymphoma	*BCL3, BCL2, CCND1, MYC*	200
2. Breast cancer	*SLC22A18, TP53, TSG101*	174, 217, 239.3
3.Hemophagocytic lymphohistiocytosis	*FHL3*	288.4
4. Hepatocellular carcinoma	*APC, TP53*	155, 211.5
5. Lung cancer	*EGFR, SLC22A18*	162, 231
6. Nasopharyngeal carcinoma	*TP53*	147
7. Severe combined immunodeficiency^i^	*ADA*	279.2
8. Stomach carcinoma	*APC, IL1B, KIT*	151
9. T cell lymphomas	*MSH2*	202
10. Classical Hodgkin lymphoma	*-*	201
11. Salivary carcinoma	*-*	142
12. Wiskott-Aldrich syndrome^i^	*-*	279.12
13. X-linked lymphoproliferative disorder^i^	*-*	238.79
14. Infectious mononucleosis	*	075
15. Lymphocytic interstitial pneumonia	*	516.8
16. Oral hairy leukoplakia	*	528.6
17. Thymus carcinoma	*	164

Table 1B		
HPV-implicated diseases	Mapped genes	ICD-9 code(s)
1. Bladder cancer	*RB1*	188
2. Breast cancer	*BRCA1, CASP8, RAD54L, TP53*	174, 217, 239.3
3. Colon cancer	*APC, BAX, EP300, MSH2, ODC1, RAD54B, RAD54L, TP53*	153
4. Head and neck squamous carcinoma	*TNFRSF10B*	173
5. Ovarian cancer	*BRCA1, ERBB2, FN1, MSH2*	183, 220
6. Prostate cancer	*CD82*	185, 233.4
7. Squamous cell carcinoma of the lung	*CASP8, ERBB2, EGFR, IRF1*	162, 231
8. Carcinoma of cervix uteri	*-*	180, 233.1
9. Laryngeal carcinoma	*-*	161
10. Bowen disease	*	230–234
11. Conjunctival carcinoma	*	190.3
12. Intraepithelial neoplasia	*	233
13. Oral carcinoma	*	140, 141
14. Oral leukoplakia	*	528.6

**A**, EBV-implicated diseases. **B**, HPV16-implicated diseases. Mapped gene column lists the genes found in the neighborhood of viral targets. An asterisk (*) in the “mapped gene” column corresponds to diseases where no known genes are reportedly associated with the disease in OMIM database, and (-) corresponds to diseases where there are genes reportedly associated with the disease in OMIM database but are not identified with our approach. Diseases marked with (^i^) correspond to EBV-implicated diseases within the framework of B cell lymphoma.

To explore the role of macromolecular networks in virus-disease associations we collected four categories of biological connections: 1) lists of previously published experimental virus-human protein-protein [12],[13],[14] and protein-DNA interactions [2], [15], [16]; 2) a newly generated dataset of EBV-human and HPV16-human protein-protein interactions (Tables S8, S9 in Text S1), with sets (1) and (2) together defining our set of “viral targets”; 3) previously published experimental human protein-protein interactions [17], [18], [19], [20], [21], experimental and predicted human protein-DNA interactions [22], [23], and predicted human metabolic coupling interactions [24], all of which together define our “host interactome”; and 4) human gene/disease associations [11] which define a set of human genes associated with human diseases (Text S1).

### Local impact hypothesis

**Figure 1:**
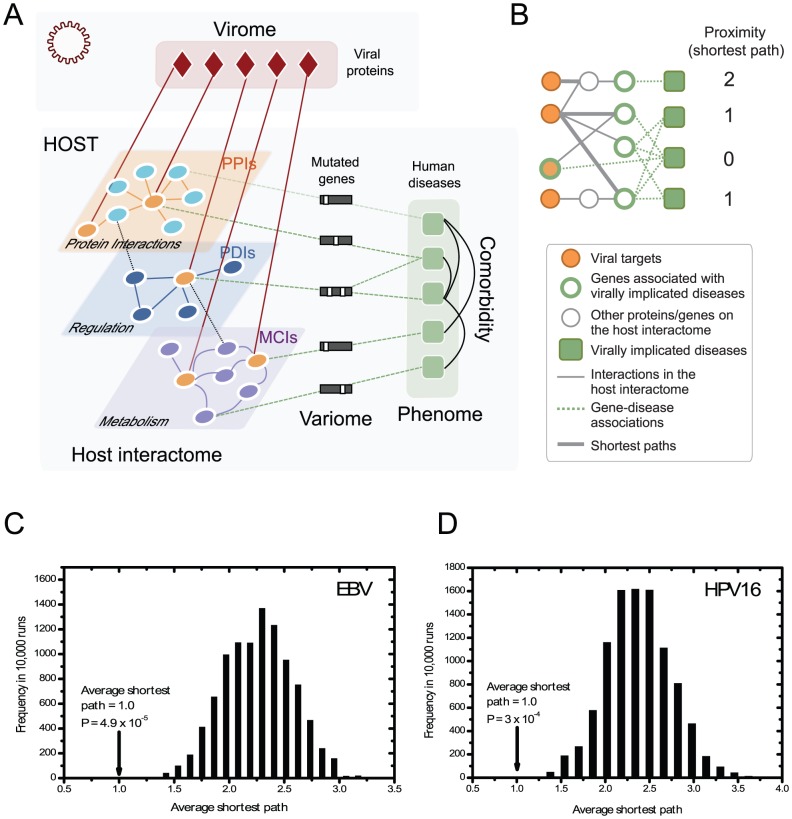
Linking a viral proteome to virally implicated diseases through the host interactome. **A**, Viral proteins (virome) interact with host proteins (viral targets) in the host interactome, which in turn are linked to various human diseases (phenome) through mutations in particular disease susceptibility genes (variome). **B**, Determining topological proximity between viral targets and genes associated with virally implicated diseases by measuring the shortest path lengths between them. For each disease, the minimum number of hops of interactions needed to connect any of its associated genes to any viral targets is designated as the shortest path. **C,D**, The average shortest path for either EBV (C) or HPV16 (D) was significantly shorter than random expectation.

To test our hypothesis that genes associated with virally implicated diseases are located in the network vicinity of viral targets (Figure 1A), we measured the shortest paths, defined as the minimum number of “hops” along the links of the host interactome from viral targets to genes associated with a given virally implicated disease (Figure 1B). For either EBV or HPV the average shortest path (averaged over the number of virally implicated diseases) is significantly shorter than when virally implicated diseases were replaced with randomly sampled human diseases in OMIM (Figures S2, S3 in Text S1; *P* = 2.3×10^−6^ for EBV and *P* = 7×10^−7^ for HPV16, based on empirical calculation). That this shortest path was less than one for both EBV (0.667) and HPV (0.5) indicates that viral proteins preferentially target a disease associated protein directly (hop 0) or a protein that directly interacts with a disease associated protein (hop 1). To mitigate potential investigational biases that accompany literature-curated datasets [25], we also examined the average shortest path upon removal of small-scale protein-protein and protein-DNA interactions from the host interactome, leaving only interactions derived from high-throughput investigations. The average shortest path remained significantly shorter than random (average shortest path = 1.0; *P* = 4.9×10^−5^ for EBV and average shortest path = 1.0; *P* = 3×10^−4^ for HPV16, based on empirical calculation; Figure 1C,D). The shortness of the path lengths between viral targets and genes associated with virally implicated diseases is mostly due to the tendency of the viral targets being hubs, and to a lesser degree to the properties of disease genes (Text S1).

The relative shortness of the paths from viral targets to disease genes validates the hypothesis that genes in the “neighborhood” of viral targets are more likely associated with virally implicated diseases, compared to genes in distant regions of the host interactome. But still, given the small world nature of the interactome, large numbers of proteins are within a few hops of the viral targets, potentially implicating hundreds of diseases for which there is no known relationship to HPV or EBV. Accordingly, a procedure is needed to identify the set of host cellular components (genes, proteins, and metabolites) that are most likely impacted by the virus, representing the network neighborhood of viral targets. Do the three kinds of interactions used to build the interactome — protein-protein, metabolic and regulatory interactions — play a comparable role in linking viral targets to virally-implicated diseases, and how deep into the interactome should one go, keeping in mind that most proteins are approximately three links from the viral proteins.

**Figure 2:**
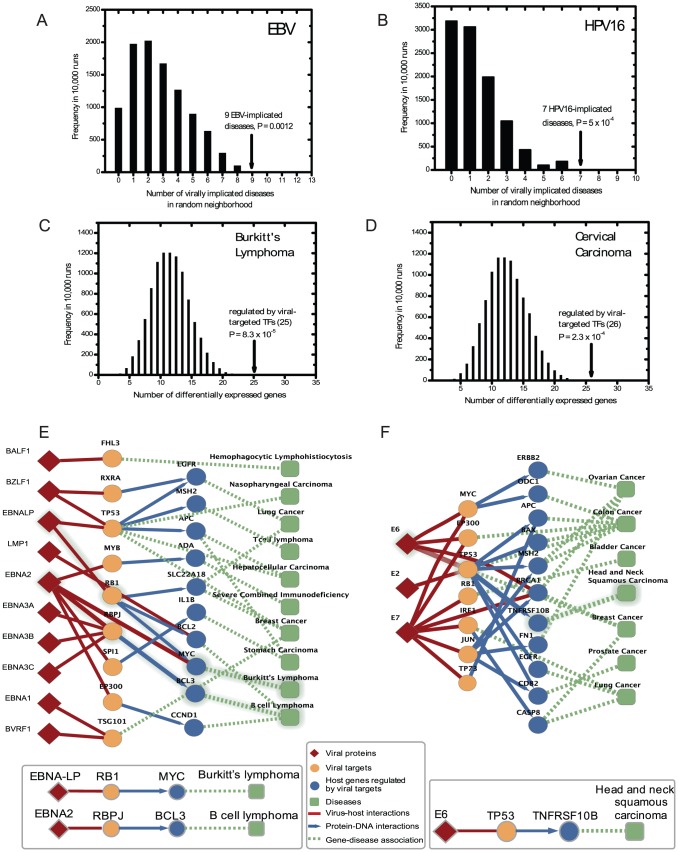
Virally implicated diseases associated with genes in the neighborhoods of viral targets. **A,B**, The number of virally implicated diseases in the neighborhoods was higher than randomly expected for EBV (A) and HPV16 (B). **C,D**, The number of differentially expressed genes in the neighborhood of viral targets of either EBV (C) or HPV16 (D) was significantly higher compared to that in the neighborhood of randomly sampled host genes. The total number of genes regulated by EBV and HPV targets is 109 and 122, respectively. Expression level was measured in tissues of two virally implicated diseases respectively, Burkitt's lymphoma (EBV) and cervical cancer (HPV), and compared to normal tissues. **E,F**, Known virally implicated diseases in the vicinity of viral targets for EBV (E) and HPV16 (F). Examples of paths that are known to correspond to disease mechanism are highlighted in grey and listed individually underneath.

To find the optimal neighborhood responsible for the phenotypic impact of a virus, we tested several “configurations” that govern the maximum hops allowed from the viral targets for each type of biological interaction. The simplest configuration includes only viral targets, while the more extended configurations capture increasing number of hops along the links of the interactome network, connecting an increasing number of proteins. The best configuration, as measured by the odds ratio of the enrichment of virally implicated diseases, defined the optimal neighborhood as the viral targets themselves and the genes regulated by them, and was the same for both viruses (Figure S4A,B in Text S1; Tables S3,S4 in Text S1). This agrees with our finding that genes associated with virally implicated diseases are themselves viral targets or are the interaction partners of viral targets (local impact hypothesis). For both viruses protein-protein and metabolic interactions in the host interactome were of secondary importance in linking the viral targets to the diseases they cause. For other viruses, however, these interactions could prove to be important. Indeed, analyses restricted to high-throughput data suggested additional relevance of host protein-protein and metabolic interactions (Figure S4C,D in Text S1; Tables S3,S4 in Text S1). In the selected optimal configuration, 9 out of the 13 virally implicated diseases for EBV were associated with genes in the neighborhood of EBV targets (Table 1a), and 7 out of 9 virally implicated diseases for HPV were associated with genes in the neighborhood of HPV16 targets (Table 1b). Both of these numbers were significantly higher than random expectation (Figure 2A,B; *P* = 0.0012 for EBV and *P* = 0.0005 for HPV based on empirical calculation). We therefore chose this configuration to define the network neighborhood of the viral targets, representing the viral disease modules, leaving aside the host metabolic and protein interactions.

According to the local impact hypothesis, the genes regulated by viral targets should have significantly altered expression levels in virally implicated disease tissues within the viral disease modules. To test this, we collected microarray gene expression data for two representative EBV-implicated diseases, Burkitt's lymphoma and B cell lymphoma [26], and for two HPV16-implicated diseases, cervical cancer [27] and head and neck squamous cell carcinoma [28] (Methods). We compared gene expression levels between disease tissues to control (unaffected) tissues. We defined genes with significantly altered expression levels (“differentially expressed genes”) as those whose changes in expression level between disease and normal tissues were among the top or bottom 5% of all genes (conclusions were unaltered across a wide range of cutoffs) (Table S5 in Text S1 and Text S1). In disease samples there were significantly more differentially expressed genes in the neighborhood of viral targets of either EBV or HPV16 than in the neighborhood of randomly sampled host genes that are regulated by at least one transcription factor in the TRANSFAC database (Figure 2C,D; Figures S5, S6 and Table S5 in Text S1).

### Viral disease network

Given the high interconnectivity of the host interactome, the number of all potential distinct paths linking viral targets to genes (or gene products) associated with virally implicated diseases exceeds 10^200^ for both viruses (Text S1). Yet, the local impact hypothesis argues that only paths within the neighborhood of viral targets might play a mechanistic role in virally implicated diseases. These paths, defined as the shortest paths between the set of viral targets and genes associated with virally implicated diseases, are much fewer (20 for EBV and 24 for HPV), and could be inspected individually to determine whether they may contribute to known disease mechanisms and whether they predict potentially novel links between viruses and virally implicated diseases. Several of these paths are already informative upon disease mechanisms (highlighted in Figure 2E,F): i) EBV protein EBNA-LP has been shown to bind to RB1, which in turn regulates *MYC*, a human gene associated with Burkitt's lymphoma, an EBV-implicated disease [2], [10]; ii) EBV protein EBNA2 binds to host protein RBPJ [2] which regulates *Bcl-3* [29], which is in turn associated with B cell lymphoma, an EBV-implicated disease [2], [10]; and iii) HPV E6 protein interacts with p53 which regulates *TNFRSF10B* which is associated with head and neck squamous carcinoma, an HPV16-implicated disease [30]. The many other suggestive paths uncovered between viral targets and genes associated with virally implicated diseases (Figure 2E,F) represent candidates for focused investigations into the molecular mechanisms of these diseases.

**Figure 3:**
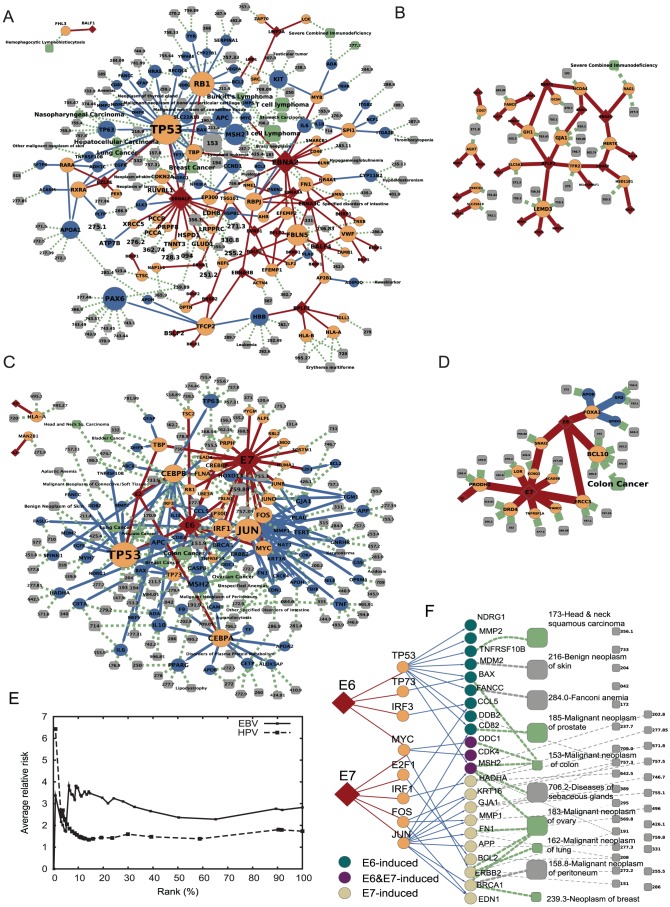
Viral disease networks. **A,C**, The neighborhoods of viral targets in the host interactome, along with their disease associations, represent “viral disease networks”. Diseases associated with genes in the neighborhood of EBV (A) or HPV16 (C) targets that are not yet characterized with viral implications are shown as grey nodes. Node size is proportional to the degree of a node (number of neighbors it has) in the viral disease network. **B,D**, Diseases associated with genes in the neighborhoods of randomly generated viral targets of EBV (B) or HPV16 (D) are significantly sparser than the neighborhoods of actual viral targets. **E**, Benchmarking the prioritization using relative risk with virally infected patients showed that the higher-ranked diseases in the prioritization are more often associated with viral infection. **F**, Differentially expressed genes in E6 or E7 induced IMR90 and HFK cell populations with their associated diseases. If a gene is regulated by a specific viral protein target, it is also almost always differentially expressed in the cell population where that specific viral protein is induced. For example, *EDN1* is regulated by FOS, an E7 target, and EDN1 is differentially expressed in E7 induced cell populations. Large grey nodes: diseases with high relative risk among HPV patients.

The neighborhoods of viral targets in the host interactome, along with their disease associations, represent “viral disease networks” (Figure 3A,C). The viral proteins, their viral targets, the proteins in their local neighborhood and diseases associated with all the host genes are included in the disease network. In line with the local impact hypothesis, we expect that these neighborhoods contain most cellular components that play a role in the phenotypic impact of the virus on the host. The neighborhood of randomly chosen human proteins as viral targets yields a much sparser and smaller network (Figure 3B,D), indicating that the observed viral disease networks had not emerged by chance, but instead reflect the functional adaptation of viruses to the host interactome. Randomly chosen degree-controlled viral targets also yielded random disease networks with significantly smaller connected components (Figure S8 in Text S1).

### Prioritizing virally implicated diseases

The uncovered viral disease networks contain several diseases that have not been previously associated with infection by the corresponding viruses (grey squares in Figure 3A,C). These diseases arise by mutations in cellular pathways that are targeted by these viruses. Some of these diseases might arise from infection with HPV or EBV. Given the large number of such disease candidates (128 for EBV and 141 for HPV), it is important to prioritize them based on their proximity to viral targets, inferring the likelihood that the virus-induced perturbations could contribute to the particular disease phenotype. We implemented a topology-based network flow algorithm [31] that simultaneously exploits the local modularity of the interactome and the non-random placement of the disease associated components in the network. Initially, only the viral targets have non-zero scores, and the score of other proteins in the entire interactome is zero. The algorithm iteratively distributes scores to host genes based on their potential association with viral perturbation, prioritizing the genes in the neighborhood of the viral targets. Using literature-derived virally implicated diseases (Table 1) as a positive reference set, we evaluated the precision-recall performance of the prioritization for both EBV and HPV16 (Figure S9 in Text S1) and found enrichment of virally implicated diseases among the high-ranking diseases (*e.g.* Burkitt's lymphoma for EBV and bladder cancer for HPV16, Tables S6, S7 in Text S1), supporting the feasibility of the prioritization procedure.

To independently benchmark the prioritization of candidate diseases, we turned to relative risk measurement [24], [32], [33], which provides population-based clinical associations between candidate diseases and viral infection in patients (Text S1). Using U.S. Medicare patient medical history data [24], [34] derived from 13 million patients, we found that higher-ranked diseases in the prioritization are more often associated with viral infection, for either EBV or HPV (Figure 3E). This comorbidity analysis indicates that diseases with associated genes in the network vicinity of viral targets are strong candidates for being virally implicated. The prioritized virally-implicated disease candidates (Tables S6, S7 in Text S1) indicate, for example, that malignant neoplasms of retina and bladder, ranked in the top three by the flow algorithm regarding their potential association with HPV, have relative risk 15.7 and 2.7 (Table S7 in Text S1), meaning that HPV patients have 15.7 and 2.7 times increased chance of developing these diseases. Several diseases that ranked high in our prioritization procedure are not commonly linked to the studied viruses, but their potential viral association was supported by recent suggestive reports, such as malignant neoplasm of thyroid gland association with EBV [35] and retinoblastoma association with HPV [36], [37].

### Experimental validation

To demonstrate the value of the network-based approach to generate new biological hypotheses, we explored whether the cellular perturbations induced by expression of individual viral proteins are similar to those seen in particular disease phenotypes. We generated primary human keratinocyte (HFK) populations with stable expression of the HPV16 E6 or E7 oncoproteins and analyzed the gene expression profiles of multiple independent samples for these cells in concert with expression data from IMR90 cells expressing HPV16 E6 or E7 proteins (Methods and Text S1). Of the 104 human genes regulated by the 15 human protein targets of E6 and E7 (*i.e.*, those two degrees away from E6 and E7 in Figure 3C), 22 were found to be differentially expressed in E6 or E7 induced IMR90 and/or HFK cell populations (Figure 3F; Table S14 in Text S1). Of these 22 genes 15 of them were also differentially expressed in cervical carcinoma tissues evaluated previously to test the local impact hypothesis (Text S1). These 22 genes have been linked to 39 diseases in OMIM, among which only six belong to known HPV-related diseases (Table 1B). We therefore asked if any of the remaining 33 diseases might be virally implicated (Figure 3F). Illustrative of our approach is ovarian cancer, which is linked to HPV via three lines of evidence: (i) the disease has significant comorbidity with HPV associated diseases; (ii) four of the ovarian cancer associated genes in the disease network are differentially expressed in E6 or E7 induced IMR90 or HFK cell populations; (iii) three of these, *FN1, BRCA1, ERBB2*, are differentially expressed in ovarian carcinoma tissues (GEO dataset: GDS3592; two-tailed t-test; *P*<0.05) [38].

Seven out of 39 diseases have high relative risks among HPV patients (Figure 3F), of which four are previously unknown. Among these four diseases, neoplasm of peritoneum, benign neoplasm of skin, and diseases of sebaceous glands satisfied only two of the three criteria (Text S1). Fanconi anemia, the fourth disease on the list, satisfied all three in that (i) Fanconi anemia shows high relative risk with HPV; (ii) *FANCC*, a gene in the disease network mutated in Fanconi anemia, is up-regulated in the E6 exogenous expression IMR90 cell data, and (iii) *FANCC* is significantly up-regulated in low density bone marrow cells of Fanconi anemia patients (GEO dataset: GSE16334; two-tailed t-test; *P* = 0.00069) [38]. In addition, HPV16 E7 was hypothesized to induce expression of *FANCD2* through an E2F-dependent pathway [39], [40], a finding that is also supported by our analysis (Text S1). Our analysis predicts a novel potential link between HPV and Fanconi anemia, through the E6→TP53→*FANCC* pathway, which had not been previously established. FANCC has been reported to be expressed at higher levels upon activation of TP53 [41] but since E6 targets TP53 for degradation it is unlikely that the observed upregulation of FANCC expression in E6 expressing cells is solely modulated by p53. An additional connection to Fanconi anemia may be through interaction with BRCA1 [42] (Figure 2F). In addition to a physical interaction of E6 and E7 with BRCA1, BRCA1 expression is upregulated in E6 expressing cell lines (Table S14 in Text S1). BRCA1 has been shown to have a potential role in Fanconi anemia through its role in the colocalization of FANCD2 protein [43], [44].

The clinical connection between Fanconi anemia and HPV associated tumors has been subject to debate. Not debatable is that FA patients have a much-increased risk in developing squamous cell carcinomas (SCCs) at anatomical sites infected by HPVs. Our analysis does not necessarily mean that SCCs in Fanconi patients are caused by HPV, but that they arise by similar molecular mechanisms. The well-documented interplay between E7 and FA and our discovery of a possible connection between E6, FANCC and BRCA1 support this hypothesis. Moreover, we observe a relative risk of 3.7 among female HPV patients (mostly cervical cancer patients) toward Fanconi anemia using the US-wide Medicare data, which further supports the identified molecular level relationship between Fanconi anemia and HPV (Methods and Text S1).

## Discussion

Given the large number of functional interactions present in human cells and the many possible paths among cellular components, uncovering the precise impact of a virus upon the host interactome is an enormously complicated task. Here we provide evidence that a large proportion of the effect of a virus can be accounted for locally in the network space, which allowed us to develop and test a general methodology designed to elucidate the consequences of viral impacts on the host interactome network, and to prioritize candidate diseases for potential viral implications.

A predictive methodology should ideally take into account cell tropism. Tissue-specific gene expression data can be merged with our analysis (Text S1). We used tissue-specific expression data from BIOGPS [45] to narrow down the number of genes and their associated diseases from the diseasome map. If a gene in the neighborhood of the viral targets is not expressed or is not present in the tissue of interest, we removed the gene from the network. In this way, we obtain a tissue-specific viral network. By applying tissue specificity, the number of associated diseases for EBV was reduced from 128 to 89, and for HPV from 141 to 105, without losing any of the virally-implicated diseases (Text S1 for analysis details; Tables S15, S16 in Text S1 for the list of genes and diseases).

The strategy developed here is not unique to EBV and HPV16. Although the strategy should work better for carcinogenic pathogens, given how well-studied proteins involved in cancer are, it is equally applicable to any pathogen for which protein interactions between the pathogen and the host proteome have been mapped. While still limited by the incompleteness of genome- and proteome-scale datasets [19], the usefulness of the method is likely to grow alongside the ongoing expansion of high-throughput functional genomics databases and gene-disease associations.

## Methods

### Virus-host protein-protein interactions

Yeast two-hybrid screens (Y2H) between EBV and HPV16 viral proteins and ∼12,200 human proteins encoded by a library of full length human open reading frame (ORFs) clones in Human ORFeome v3.1 [46], [47] encompassing ∼10,200 human genes were carried out as before [17], [48]. The EBV-human library Y2H screen tested 86 out of 89 EBV proteins as fusions to the DNA binding domain of Gal4 (Gal4-DB) against ORFeome v3.1 proteins fused to the activation domain of Gal4 (Gal4-AD), while the HPV-human library Y2H screen testing HPV16 proteins E4, E5, E6 and E7 was carried out in reciprocal fashion with HPV proteins as both Gal4-DB and Gal4-AD fusions against the corresponding human Gal4-AD and Gal4-DB fusions, respectively.

### Differentially expressed genes in virally implicated disease tissues

Raw data of the gene expression datasets used (GSE2350, GSE2392 and GSE15156) was obtained from Gene Expression Omnibus (GEO) [49], normalized and log-transformed by RMA algorithm [50], and expression changes were calculated as the ratio of expression levels between virus-infected tissues and normal tissues.

### Exogenous expression of HPV viral proteins in human cell lines

To obtain the disease associated genes that are differentially expressed in viral protein induced cell populations, HPV16 E6 and E7 oncogenes were independently transfected into primary human fibroblast (IMR90) and keratinocyte (HFK) cell populations. Affymetrix Human Gene1.0 ST and Human Genome U133 Plus 2.0 arrays, respectively, were used to measure gene expression profiles for five or more replicate samples in each of the cell types. Array data were normalized by RMA, batch effects were removed using ComBat, and the limma package in R/Bioconductor was used to identify differential expression.

### Comorbidity analysis and relative risk calculation

Relative risk (RR) was calculated as the ratio between the observed co-occurrence and probabilistically-inferred (assuming independence) co-occurrence of two diseases, based on the patient medical history data from United States (U.S.) Medicare, which contains the clinical diagnosis record of each hospital visit (in ICD-9 codes) of 13 million U.S. patients at age 65 or older [33]. Patients with viral infections were defined with the following diagnostic codes in U.S. Medicare database: 200 (B cell lymphoma) or 147 (nasopharyngeal carcinoma) for EBV infections; 078.1, 079.4, 180 or 795.0 for HPV infections.

### Statistical tests

The statistical significance of the average shortest path between viral targets and genes associated with a given virally implicated disease was calculated by randomly sampling human diseases from OMIM (full table of disease in Dataset S2). The number of virally implicated diseases associated with the proteins in the neighborhoods of random host targets was calculated by picking random proteins from the interactome space (n = 7,832). For both measurements, *P* values were calculated based on empirical data with 10,000 random configurations. For the analysis of GEO microarray data we used two-tailed t-test statistics.

## Supporting Information

Dataset S1 Full list of interactions and gene-disease associations in viral disease networks, including the sources of data. (**Sheet 1**) EBV disease network, (**Sheet 2**) HPV16 disease network. VH-PPI: virus-host protein-protein interaction, PPI: host protein-protein interaction, PDI: host protein-DNA interaction, MCI: metabolic enzyme-coupled interactions calculated using KEGG, or BIGG databases, or flux coupling analysis.
(XLS)

Dataset S2 OMIM genes and diseases and their corresponding ICD-9 codes.
(XLS)

Dataset S3 Relative risk analysis. **(Sheet 1, 3)** relative risk between EBV- and HPV-implicated diseases and candidate diseases in the disease network. **(Sheet 2, 4**) relative risk between EBV- and HPV-implicated diseases and all mappable diseases which constitute the random control.
(XLS)

Dataset S4 Full list of diseases prioritized by the flow algorithm for (**Sheet 1**) EBV (**Sheet 2**) HPV. Diseases are sorted according to maximum scores.
(XLS)

Text S1 Supporting information, tables and figures are provided in this document.
(PDF)

## References

[1] IARC Monographs (2005). Human Papillomaviruses.

[2] Kutok JL, Wang F (2006). Spectrum of Epstein-Barr virus-associated diseases.. Annu Rev Pathol.

[3] Cadwell K, Patel KK, Maloney NS, Liu T-C, Ng ACY (2010). Virus-plus-susceptibility gene interaction determines Crohn's Disease gene *Atg16L1* phenotypes in intestine.. Cell.

[4] Navratil V, de Chassey B, Combe CR, Lotteau V (2011). When the human viral infectome and diseasome networks collide: towards a systems biology platform for the aetiology of human diseases.. BMC Syst Biol.

[5] Foxman EF, Iwasaki A (2011). Genome-virome interactions: examining the role of common viral infections in complex disease.. Nat Rev Microbiol.

[6] Barabási AL, Oltvai ZN (2004). Network biology: understanding the cell's functional organization.. Nat Rev Genet.

[7] Goh KI, Cusick ME, Valle D, Childs B, Vidal M (2007). The human disease network.. Proc Natl Acad Sci U S A.

[8] Zhong Q, Simonis N, Li QR, Charloteaux B, Heuze F (2009). Edgetic perturbation models of human inherited disorders.. Mol Syst Biol.

[9] McLaughlin-Drubin ME, Munger K (2009). Oncogenic activities of human papillomaviruses.. Virus Res.

[10] Young LS, Rickinson AB (2004). Epstein-Barr virus: 40 years on.. Nat Rev Cancer.

[11] Amberger J, Bocchini CA, Scott AF, Hamosh A (2009). McKusick's Online Mendelian Inheritance in Man (OMIM).. Nucleic Acids Res.

[12] Calderwood MA, Venkatesan K, Xing L, Chase MR, Vazquez A (2007). Epstein-Barr virus and virus human protein interaction maps.. Proc Natl Acad Sci U S A.

[13] Chatr-aryamontri A, Ceol A, Peluso D, Nardozza A, Panni S (2009). VirusMINT: a viral protein interaction database.. Nucleic Acids Res.

[14] Huh KW, DeMasi J, Ogawa H, Nakatani Y, Howley PM (2005). Association of the human papillomavirus type 16 E7 oncoprotein with the 600-kDa retinoblastoma protein-associated factor, p600.. Proc Natl Acad Sci U S A.

[15] Mauser A, Saito S, Appella E, Anderson CW, Seaman WT (2002). The Epstein-Barr virus immediate-early protein BZLF1 regulates p53 function through multiple mechanisms.. J Virol.

[16] Rowe M, Peng-Pilon M, Huen DS, Hardy R, Croom-Carter D (1994). Upregulation of *bcl-2* by the Epstein-Barr virus latent membrane protein LMP1: a B-cell-specific response that is delayed relative to NF-κB activation and to induction of cell surface markers.. J Virol.

[17] Rual JF, Venkatesan K, Hao T, Hirozane-Kishikawa T, Dricot A (2005). Towards a proteome-scale map of the human protein-protein interaction network.. Nature.

[18] Stelzl U, Worm U, Lalowski M, Haenig C, Brembeck FH (2005). A human protein-protein interaction network: a resource for annotating the proteome.. Cell.

[19] Venkatesan K, Rual JF, Vazquez A, Stelzl U, Lemmens I (2009). An empirical framework for binary interactome mapping.. Nat Methods.

[20] Aranda B, Achuthan P, Alam-Faruque Y, Armean I, Bridge A (2010). The IntAct molecular interaction database in 2010.. Nucleic Acids Res.

[21] Ceol A, Chatr Aryamontri A, Licata L, Peluso D, Briganti L (2010). MINT, the molecular interaction database: 2009 update.. Nucleic Acids Res.

[22] Matys V, Fricke E, Geffers R, Gossling E, Haubrock M (2003). TRANSFAC: transcriptional regulation, from patterns to profiles.. Nucleic Acids Res.

[23] Mani KM, Lefebvre C, Wang K, Lim WK, Basso K (2008). A systems biology approach to prediction of oncogenes and molecular perturbation targets in B-cell lymphomas.. Mol Syst Biol.

[24] Lee DS, Park J, Kay KA, Christakis NA, Oltvai ZN (2008). The implications of human metabolic network topology for disease comorbidity.. Proc Natl Acad Sci U S A.

[25] Yu H, Braun P, Yildirim MA, Lemmons I, Venkatesan K (2008). High quality binary protein interaction map of the yeast interactome network.. Science.

[26] Basso K, Margolin AA, Stolovitzky G, Klein U, Dalla-Favera R (2005). Reverse engineering of regulatory networks in human B cells.. Nat Genet.

[27] Kravchenko-Balasha N, Mizrachy-Schwartz S, Klein S, Levitzki A (2009). Shift from apoptotic to necrotic cell death during human papillomavirus-induced transformation of keratinocytes.. J Biol Chem.

[28] Slebos RJ, Yi Y, Ely K, Carter J, Evjen A (2006). Gene expression differences associated with human papillomavirus status in head and neck squamous cell carcinoma.. Clin Cancer Res.

[29] Oswald F, Liptay S, Adler G, Schmid RM (1998). NF-κB2 is a putative target gene of activated Notch-1 via RBP-Jκ.. Mol Cell Biol.

[30] Gillison ML, Koch WM, Capone RB, Spafford M, Westra WH (2000). Evidence for a causal association between human papillomavirus and a subset of head and neck cancers.. J Natl Cancer Inst.

[31] Vanunu O, Magger O, Ruppin E, Shlomi T, Sharan R (2010). Associating genes and protein complexes with disease via network propagation.. PLoS Comput Biol.

[32] Rzhetsky A, Wajngurt D, Park N, Zheng T (2007). Probing genetic overlap among complex human phenotypes.. Proc Natl Acad Sci U S A.

[33] Hidalgo CA, Blumm N, Barabasi AL, Christakis NA (2009). A dynamic network approach for the study of human phenotypes.. PLoS Comput Biol.

[34] Park J, Lee DS, Christakis NA, Barabasi AL (2009). The impact of cellular networks on disease comorbidity.. Mol Syst Biol.

[35] Shimakage M, Kawahara K, Sasagawa T, Inoue H, Yutsudo M (2003). Expression of Epstein-Barr virus in thyroid carcinoma correlates with tumor progression.. Hum Pathol.

[36] Mohan A, Venkatesan N, Kandalam M, Pasricha G, Acharya P (2009). Detection of human papillomavirus DNA in retinoblastoma samples: a preliminary study.. J Pediatr Hematol Oncol.

[37] Anand B, Ramesh C, Appaji L, Kumari BS, Shenoy AM (2011). Prevalence of high-risk human papillomavirus genotypes in retinoblastoma.. Br J Ophthalmol.

[38] Barrett T, Troup DB, Wilhite SE, Ledoux P, Evangelista C (2010). NCBI GEO: archive for functional genomics data sets–10 years on.. Nucleic Acids Res.

[39] Spardy N, Duensing A, Charles D, Haines N, Nakahara T (2007). The human papillomavirus type 16 E7 oncoprotein activates the Fanconi anemia (FA) pathway and causes accelerated chromosomal instability in FA cells.. J Virol.

[40] Tategu M, Arauchi T, Tanaka R, Nakagawa H, Yoshida K (2007). Systems biology-based identification of crosstalk between E2F transcription factors and the Fanconi anemia pathway.. Gene Regul Syst Bio.

[41] Gatz SA, Wiesmuller L (2006). p53 in recombination and repair.. Cell Death Differ.

[42] Zhang Y, Fan S, Meng Q, Ma Y, Katiyar P (2005). BRCA1 interaction with human papillomavirus oncoproteins.. J Biol Chem.

[43] Garcia-Higuera I, Taniguchi T, Ganesan S, Meyn MS, Timmers C (2001). Interaction of the Fanconi anemia proteins and BRCA1 in a common pathway.. Mol Cell.

[44] D'Andrea AD, Grompe M (2003). The Fanconi anaemia/BRCA pathway.. Nat Rev Cancer.

[45] Wu C, Orozco C, Boyer J, Leglise M, Goodale J (2009). BioGPS: an extensible and customizable portal for querying and organizing gene annotation resources.. Genome Biol.

[46] Lamesch P, Li N, Milstein S, Fan C, Hao T (2007). hORFeome v3.1: a resource of human open reading frames representing over 10,000 human genes.. Genomics.

[47] Rual JF, Hirozane-Kishikawa T, Hao T, Bertin N, Li S (2004). Human ORFeome version 1.1: a platform for reverse proteomics.. Genome Res.

[48] Dreze M, Monachello D, Lurin C, Cusick ME, Hill DE (2009). High-quality binary interactome mapping.. Methods Enzymol.

[49] Barrett T, Troup DB, Wilhite SE, Ledoux P, Rudnev D (2009). NCBI GEO: archive for high-throughput functional genomic data.. Nucleic Acids Res.

[50] Gentleman RC, Carey VJ, Bates DM, Bolstad B, Dettling M (2004). Bioconductor: open software development for computational biology and bioinformatics.. Genome Biol.

