# Supplementary material for: Viral Perturbations of Host Networks Reflect Disease Etiology
Source: PLoS Comput Biol. 2012 Jun 28;8(6):e1002531. doi: 10.1371/journal.pcbi.1002531 (PMC3386155; doi:10.1371/journal.pcbi.1002531)
Supplement: Text S1 — Supporting information, tables and figures are provided in this document. (PDF) [file pcbi.1002531.s005.pdf]

Viral perturbations of host networks reflect disease etiology

Supplementary Information

Table of Contents

Datasets 1
1) Virus-host PPI screens........ 1
1a. EBV-human PPIs........ 2
1b. HPV16-human PPIs. 2
2) Host Interactome........ 3
a) Protein-protein interactions (PPIs)........ 3
b) Protein-DNA interactions (PDIs). 3
c) Metabolic enzyme-coupled interactions (MCIs)........ 4
3) Gene-disease associations. 5
All simple paths between viral targets and disease associated genes 6
Systematic determination of best network configuration 6
Expression analysis 8
Overlooked virally implicated diseases........ 10
Random disease networks 11
Clinical data and relative risk analysis 12
a. HPV patients........ 13
b. EBV patients........ 14
c. Average relative risk between candidate diseases and virally implicated diseases.... 14
Prioritizing candidate diseases for viral implication 15
Tissue specificity 20
EBV presence in disease tissues........ 21
Topological properties of viral targets and viral disease networks 22
Giant component size of the disease network........ 22
Disease gene properties. 22
List of supplementary files 23
Supplementary figures and tables 24

Datasets

To systematically analyze the viral perturbations of host networks, we collected three categories of biological connections: 1) virus-host interactions; 2) host-host interactions; and 3) human gene-disease associations.

1) Virus-host PPI screens. Experimentally-derived PPI data for EBV-Human and HPV16-Human interactions is from Rozenblatt-Rosen et al (*Nature*, in press) and obtained as follows. Yeast two-hybrid screens (Y2H) between EBV and HPV16 viral proteins and ~12,200 human proteins encoded by a library of full length human open reading frame (ORFs) clones in Human ORFeome v3.1 collection [1,2], encompassing ~10,200 human genes, were carried out as before [3,4]. The EBV-human library Y2H screen tested 86 out of 89 EBV proteins as fusions to the DNA binding domain of Gal4 (Gal4-DB) against ORFeome v3.1 proteins fused to the activation domain of Gal4 (Gal4-AD), while the HPV-human library Y2H screen testing HPV16 proteins E4, E5, E6 and E7 was carried out matrix-style in reciprocal fashion with HPV proteins as both Gal4-DB and Gal4-Ad fusions against the corresponding human Gal4-AD and Gal4-DB fusions, respectively. The primary EBV and HPV Y2H screens against our human library of ~12,200 proteins were each performed twice, and any Y2H positives that passed secondary phenotyping and autoactivator controls in either screen [3,4] were then retested by mating fresh glycerol stocks of the relevant haploid yeast cells of opposite mating-type containing virus or human DB-X or AD-Y expression plasmids to confirm reproducible Y2H phenotypes. Retesting of all EBV-human positive pairs was carried out twice and an EBV-human pair was considered confirmed if

both retests were positive with no latent autoactivators appearing in any retest. HPV-human retesting was performed four times in both orientations (DB-HPV vs AD-Hu and DB-Hu vs AD-HPV) and an HPV-human pair was considered confirmed if at least three retests out of four for either orientation were positive with no latent autoactivators appearing in any of the four orientation-specific retests.

1a. EBV-human PPIs. The EBV-host interaction dataset used herein is compiled from previously published EBV-human protein-protein yeast two-hybrid interaction data [5], newly generated yeast two-hybrid data (Rozenblatt-Rosen, et al), literature-curated interactions from the VirusMINT database [6], and an additional set of interactions we curated from the primary literature (Supplementary Table S1). In total the compiled dataset contains 460 PPIs among 49 viral and 336 human proteins. The EBV *comprehensive dataset* is provided in Supplementary Table S8. To mitigate inspection bias all of the analyses we report were also repeated with high-throughput datasets only, excluding interactions coming from literature-curated and small-scale datasets. The high-throughput EBV-human PPIs include previous Y2H data [5] and the Y2H dataset from Rozenblatt-Rosen, et al, , which combined have 287 interactions among 49 viral and 177 human proteins.

1b. HPV16-human PPIs. The HPV16-host interactions combines newly generated Y2H virus-human protein-protein interaction data from Rozenblatt-Rosen, et al, published tandem affinity purification (TAP) co-complex membership data [7], and literature-curated virus-human protein-protein interactions reported in VirusMINT [6] which we re-curated to minimize

curation errors (Supplementary Table S2). The HPV16 comprehensive dataset includes 205 interactions among 8 viral proteins and 189 human proteins where most of the interactions involve three HPV16 proteins, E7 (147 interactions) and E6 (31 interactions) and E2 (19 interactions) (Supplementary Table S9). For the high-throughput dataset, we used the Y2H and the tandem affinity purification co-complex membership datasets and excluded the curated interactions. This high-throughput dataset includes 124 interactions among two viral proteins (E6 and E7) and 123 human proteins.

2) Host Interactome. The host interactome includes human protein-protein interactions (PPIs), protein-DNA interactions (PDIs), and metabolic enzyme-coupled interactions (MCIs) (Supplementary File S1).

a) Protein-protein interactions (PPIs). For human PPIs, we collected binary interactions reported in IntAct [8] and MINT [9] databases, combining these with three Y2H-based high-throughput datasets [3,8,9,10,11]. For the high-throughput dataset, we removed literature-curated PPIs and only used the three Y2H-based datasets. Host PPIs were used in the P^2, P^2T and P^2TM configurations (see *Systematic determination of best network configuration*).

b) Protein-DNA interactions (PDIs). For protein-DNA interactions, we used version 2008.2 of TRANSFAC database [12] for both viruses. For EBV, we also included the literature-curated set of B cell interactome [13] given that EBV is mainly present in B cells. The TRANSFAC database contains mostly literature-curated, plus some predicted PDI data based on the experimentally-

proven binding sites of transcription factors and the regulated genes [12]. TRANSFAC 2008.2 contains 271 human transcription factors and 564 regulated genes. Quality control information of TRANSFAC is available at http://www.gene-regulation.com/info/qm.html. Within TRANSFAC and B cell interactome datasets, there are 19 transcription factors targeted by EBV, and another 122 genes are regulated by these virus-targeted transcription factors, for a total of 138 PDIs. For HPV16 there are 17 transcription factors targeted by the virus and 129 genes regulated by these virus-targeted transcription factors, for a total of 176 PDIs. Host PDIs were used in the PT, PTM, P^2T, P^2TM configurations (see *Systematic determination of best network configuration*).

c) Metabolic enzyme-coupled interactions (MCIs). Metabolic enzyme-coupled interactions were defined by metabolic coupling analysis [14] which measures substrate sharing through adjacent reactions and flux coupling.

Substrate sharing through adjacent reactions: If the same substrate is shared between two metabolic reactions, the scarcity or abundance of that substrate may affect the fluxes of both reactions, potentially coupling their activity. Following precedent [14], we considered two metabolic reactions and hence their catalyzing enzyme to be linked if they process a common metabolite, *i.e.* if they are adjacent to each other in a metabolic reaction map. A threshold is introduced where only the metabolites that are involved in no more than four reactions are considered. We used the KEGG [15] and BIGG [16] databases as sources for metabolic reactions, with a total of 1,729 enzymes.

Flux coupling: By using the flux coupling finder methodology (FCA) [17], we identified two types of coupling between pairs of reactions *i* and *j*: (*i*) directional coupling ($i \rightarrow j$), if a nonzero flux for *i* implies a nonzero flux for *j* but not necessarily the reverse; or (*ii*) full coupling ($i \rightarrow j$), if a nonzero flux for *i* implies not only a nonzero but also a fixed flux for *j* and vice versa. Details of the flux coupling analysis were previously discussed [14]. We applied FCA to the human metabolic network construction, *Homo sapiens* Recon 1, to obtain flux coupling information [18].

A metabolic coupling interaction exists between two enzymes if they share adjacent reactions in the KEGG database or in the BIGG database, or if they are flux-coupled via FCA. Of the 336 human proteins with which EBV viral proteins interact, 21 proteins are metabolic enzymes. An additional 303 enzymes were coupled to these virus-targeted enzymes by metabolic coupling. For HPV, of the 189 targeted human proteins, 16 are metabolic enzymes. An additional 354 enzymes were found to be coupled to these virus-targeted enzymes by metabolic coupling analysis. The defined MCIs were used in the PTM, P^2M and P^2TM configurations (Section *Systematic determination of best network configuration*). The full list of interactions is provided (Supplementary File S1).

3) Gene-disease associations. We gathered information on human genes associated with diseases from the Online Inheritance in Man (OMIM) database, a comprehensive compendium of human genes and genetic phenotypes [19]. Specifically we used the OMIM Morbid Map repository (Feb 18, 2009), which lists genes and diseases in a machine parse-able format,

and also presents the cytogenetic map location of disease genes. To map the OMIM disease names to International Statistical Classification of Diseases and Related Health Problems version 9 (ICD-9) codes, we used the manually curated list of human inherited diseases [20] as a baseline and updated it as necessary. The gene-disease associations list is provided (Supplementary File S2).

All simple paths between viral targets and disease associated genes

In the main text we mentioned that the number of potential pathways linking viral targets to genes (or gene products) associated with virally implicated diseases is greater than 10^{200}. In essence the problem is to count the number of all simple paths[1] between a pair of nodes on a connected graph. In graph theory this well-discussed problem was shown to be NP-complete in 1979 [21]. Using a recently published Monte Carlo approach [22], we estimated the total number of non-shortest paths to be greater than 10^{200} (P = 0.001), which is practically infinity for our investigation.

Systematic determination of best network configuration

Given the small-world property of current interactome models [23,24], most of the host interactome would be covered in the 3- or 4-hop neighborhood of viral targets. Hence, thresholds need to be applied to avoid reaching too many irrelevant proteins, a concept

[1] **Simple path**: A path with no repeated vertices.

commonly called *network pruning* in graph theory [25]. We define such a threshold as a *network configuration*, which restricts the maximum number of hops allowed for each type of interactions (PPI, PDI and MCI) (Supplementary Figure S1). The simplest configuration "P" includes only viral targets, while the "PT" configuration includes viral targets and host genes regulated by them via PDI, and the "PM" configuration includes viral targets and host enzymes coupled to them via MCI. The more extended configurations, PTM, P^2T, P^2M, P^2TM, capture increasing maximum number of hops along the links of the PPI, PDI and MCI networks, reaching systematically deeper into the interactome, and involving an increasing number of proteins.

We determined the optimal configuration by calculating the p-values and odds ratios for the enrichment of virally implicated diseases associated with host genes in the neighborhood, with optimal configuration defined by Fisher's exact test. The sets of virally implicated diseases (13 for EBV and 9 for HPV16) and number of diseases (696) in OMIM-ICD9 space (Supplementary File S2) were used in the calculation. Supplementary Tables S3 and S4 show the p-values and odds ratios with and without the literature-curated virus-host interactions and host PPIs for each configuration.

	Virally implicated	Not virally implicated
Mapped in the configuration	9	119
Not mapped in the configuration	4	(696-119-9-4) = 564

Fisher's exact contingency table of the PT configuration for EBV using the comprehensive dataset. "Mapped" refers to diseases being associated with genes in the neighborhood defined by the PT configuration.

The optimal network configuration depended on the nature of the virus-host interaction datasets. For both EBV and HPV16 the "PT" configuration was the optimal configuration with the lowest p-value and highest odds ratio, with high-throughput and literature curated virus-host interactions combined (Supplementary Figure S4). When we limited the datasets to high-throughput datasets only (hence excluding literature-curated interactions), P^2 (EBV) and PM (HPV16) were the optimal configurations (Supplementary Figure S4).

Expression analysis

After normalizing and log-transforming raw expression data by the RMA algorithm [26], expression changes were calculated as the ratio of expression levels between virus-infected tissues and normal tissues. To identify differentially expressed genes the steps were: (1) obtain log-transformed gene expression levels with and without viral infection, L_w and L_{wo} respectively, (2) calculate the expression-fold changes by using the ratio $R=2^{(L_w-L_{wo})}$, (3) find the genes with significant R on the microarray (alpha=0.05, two-tails), (4) determine how many of the genes that show significant expression change are hit by the virus (regulated by virus-targeted transcription factors) or hit by the transcription factors among randomly sampled genes, (5)

calculate the empirical p-value of the observed (virus-targeted) number of "differentially expressed genes" against random sampling. Randomized sampling was done 10,000 times within a pool of genes that are regulated by at least one transcription factor in the TRANSFAC database and that have non-zero expression on the microarray in normal tissues (above lowest 5% expression level). Within TRANSFAC and B cell interactome datasets, which form the transcriptome space for this study, there are 564 and 1299 genes regulated by transcription factors. The transcription factors targeted by EBV regulate 122 genes. Similarly for HPV16 129 genes are regulated by HPV16-targeted transcription factors. Hence we chose 122 and 129 genes from the transcriptome space to construct our random control.

The GEO datasets [27] used in the expression analysis are: GSE15156 (Cervical carcinoma), GSE3292 (Head and neck squamous carcinoma); GSE2350 (Burkitt's lymphoma and B cell lymphoma). Of the 861 genes differentially expressed in Burkitt's lymphoma tissues, the 25 that were shared with the EBV disease network was significantly higher than the expected 10.9 shared genes in a randomized control (P = 8.3×10^{-5}) (Supplementary Figs. S5, S6, and Supplementary Table S5). For B cell lymphoma tissues, the determined value of 21 differentially expressed genes in the EBV disease network was highly significant (P = 0.00367). For HPV16, we analyzed cervical carcinoma and head and neck squamous carcinoma tissues. In the cervical carcinoma tissues, 1229 genes were differentially expressed, of which 12.3 were expected to be differentially expressed in random control. Hence, the 26 genes shared in the HPV16 viral disease network were highly significant (P = 0.00023). Of the 1721 genes differentially expressed in head and neck squamous carcinoma tissues, the value of 24 genes in HPV16 viral

disease network was again significantly more than 12.7 shared genes in random control (P = 0.0026). The genes that show significant expression change in Burkitt's lymphoma, B cell lymphoma, cervical cancer, and head and neck squamous cell carcinoma are listed (Supplementary Table S10).

We also tested two different cutoffs for determining the differentially expressed genes by using the top/bottom 2% and 10% scoring genes (alpha=0.02 and 0.10, two-tails). For the 2% cutoff, we observed 12 (P = 0.0015), 11 (P = 0.0046), 12 (P = 0.0039), and 13 (P = 0.0018) genes regulated by viral targets to be differentially expressed in Burkitt's lymphoma, B cell lymphoma, cervical carcinoma and head and neck squamous carcinoma tissues, respectively (Supplementary Table S5). For the 10% cutoff, we observed 36 (P = 0.00176), 40 ($P = 6.77 \times 10^{-5}$), 41 (P = 0.0006), and 31 (P = 0.224) genes regulated by viral targets to be differentially expressed in Burkitt's lymphoma, B cell lymphoma, cervical carcinoma and head and neck squamous carcinoma tissues, respectively (Supplementary Table S5). The results remained significant except for head and neck squamous carcinoma tissues with the 10% cutoff.

Overlooked virally implicated diseases

We were unable to link two and four of the HPV- and EBV-implicated diseases respectively (Table 1a, b). Since our method is based on the concept that cellular genes targeted by viruses causes their inactivation (or activation) similar to a genetic mutation that would be catalogued in OMIM [19], we will miss those diseases that are universally caused by viruses. Infectious mononucleosis, for example, a disease clearly linked to EBV infection, lacks any known susceptibility genes and is not detected (Table 1a). Over 99% of all cervical carcinoma are

caused by HPV infections and hence the cellular genes targeted by HPV oncoproteins will not be mutated and hence are not listed in OMIM. This has been impressively validated for the p53 and retinoblastoma tumor suppressors [28,29], the major cellular targets of the HPV16 E6 and E7 oncoproteins, respectively, which were found mutated in HPV negative cervical cancers but were not mutated in the HPV positive cervical cancer lines [30]. The two putative susceptibility genes for cervical carcinoma listed in OMIM for cervical cancer (FGFR3 and ST-3), would presumably occur in HPV positive tumors and hence contribute to the carcinogenic process independent of viral infection and, hence, will not be detected by our method. Adding the p53-cervical cancer link to the Morbid Map would increase the number of HPV-related virally implicated diseases to 8 out of 9. The number of virally implicated diseases in the neighborhood of HPV targets was significantly higher than randomly expected when p53-cervical cancer association was included in the OMIM Morbid Map ($P = 5 \times 10^{-5}$; Supplementary Figure S7).

Another likely reason to be unable to link some diseases is because of the incompleteness inherent to human interactome data [11] and in OMIM. As future information is assimilated, connections to these diseases may eventually appear. One noteworthy disease missing from the HPV16 viral disease network is carcinoma of cervix uteri.

Random disease networks

EBV and HPV16 disease networks are highly connected, where the size of the giant component (largest connected component) is on the order of the number of nodes in the network. To test

if the observed viral disease network could emerge by chance we generated random viral targets and measured the size of the giant component in the neighborhood of these random targets. The giant components in random disease networks are on average much smaller (P = 0.0175 for EBV; P = 0.00936 for HPV) compared to the real disease network (size = 253 for EBV and size=220 for HPV) (Supplementary Figure S8A,B), indicating that the observed viral disease network is unlikely to emerge by chance, and instead reflects the functional adaptation of the virus to the host interactome.

Clinical data and relative risk analysis

Relative risk (RR) is the ratio between the observed co-occurrence and probabilistically-inferred (assuming independence) co-occurrence of two diseases given by:

$$RR = C_{12} / C_{12}^*,$$

where C_{12}^* is the expected number of patients to have disease 1 and 2, and C_{12} is the observed number of patients that have disease 1 and 2. If diseases occurred independently, the number of patients affected by both diseases would be given by:

$$C_{12}^* = (P_1 \times P_2) / N,$$

where P_1 is the number of patients who had disease 1, P_2 is the number of patients who had disease 2, and N is the total number of patients.

The relative risks of diseases are calculated using U.S. Medicare patient medical history data [14,31]. The MedPAR records include 13,039,018 patients who were 65 years or older, and the

dates and reasons for all their hospitalizations during 1990-1993, reported in ICD-9-CM format. Each record consists of the date of visit, a primary diagnosis and up to nine secondary diagnoses, all specified by ICD-9 codes of up to five digits. The first three digits specify the main disease category, while the last two provide additional information about the disease. The ICD-9-CM classification consists of 657 different categories at the 3-digit level and 16,459 categories at 5 digits [32].

To calculate the relative risk within the 99% confidence intervals, we calculated the lower and upper bounds of confidence interval according to the Katz *et al*. method [33].

$$LB = RR \times exp\ (-2.576 \times \sigma_{12})$$

$$UB = RR \times exp\ (2.576 \times \sigma_{12})$$

where σ_{12} is given by

$$\sigma_{12} = sqrt\ (1 / C_{12} + 1 / (P_1 \times P_2) - 1 / N - 1 / (N \times N)).$$

The relative risk is taken to be significant within the 99% confidence interval if the lowerbound value is larger than 1 when RR is larger than 1, and if the upperbound is smaller than 1 when RR is smaller than 1. Otherwise we consider the relative risk to be 1, that is, random.

a. HPV patients. Four ICD-9 codes describe HPV-related diseases in the ICD-9 database: viral warts due to human papillomavirus (078.1), human papillomavirus (079.4), malignant neoplasm of cervix uteri (180.0), and abnormal Papanicolaou smear of cervix and cervical HPV (795.0). A total of 13,796 female and 1,242 male patients in the Medicare database had been diagnosed with these HPV-related diseases. ICD-9 code 180 was not relevant to male patients. Raw data

for calculating relative risks of candidate diseases among HPV patients is available in Supplementary Tables S11, S12.

b. EBV patients. The only ICD-9 code in the Medicare database specific to Epstein-Barr virus is 075 for infectious mononucleosis. Mononucleosis is an early adulthood disease and is rare in the elderly population, plus there is some evidence that mononucleosis may not be caused by EBV in elderly patients [34]. As EBV is a latent virus carried by 90% of the population, gaining the same insights as for HPV would require access to the disease history of a younger population. We could still identify the set of patients that had been diagnosed either with B cell lymphoma (ICD-9 code: 200) or nasopharyngeal carcinoma (ICD-9 code: 147), diseases well-accepted as associated with EBV. We call these 24,974 patients as "EBV patients" though the determination is through the associated diseases rather than a viral diagnosis directly. Raw data for calculating relative risks of candidate diseases among EBV patients is available in Supplementary Table S13.

c. Average relative risk between candidate diseases and virally implicated diseases. The average relative risk between 141 HPV candidate diseases and 14 HPV-implicated diseases (Table 1b) was ‹RR› = 1.985 ± 0.101, significantly higher than the observed relative risk between random OMIM diseases and the 14 HPV-implicated diseases, ‹RR› = 1.77 ± 0.06 (Mann-Whitney u-test, P = 0.035). For EBV the average relative risk between the 128 candidate diseases and the 17 EBV-implicated diseases (Table 1a) is ‹RR› = 2.802 ± 0.155, versus ‹RR› = 2.391 ± 0.17 for the random control (Mann-Whitney u-test, $P < 3.96\times10^{-5}$). These differences suggest that many

diseases in the viral disease network are not only linked by plausible molecular paths to the viral targets, but also show a clinical association to the virally implicated diseases.

Calculating the averages with the logarithm of relative risk did not change the results. For HPV, ‹log RR› = 0.172 ± 0.018, was significantly higher than the observed relative risk between random OMIM diseases and the virally implicated diseases, ‹log RR› = 0.125 ± 0.02 (unpaired t-test, $P = 0.0186$). For EBV the average relative risk between the candidate diseases and the virally implicated diseases (Table 1a) was ‹log RR› = 0.451 ± 0.02, whereas the random control yielded ‹log RR› = 0.317 ± 0.02 (unpaired t-test, $P < 0.0001$). We provide the lists of pairs of diseases with their corresponding relative risks (Supplementary File S3).

Prioritizing candidate diseases for viral implication

Disease gene prediction algorithms have been developed motivated by the observation that genes causing the same or similar diseases tend to lie close to one another in a network of protein-protein or functional interactions [35]. A promising algorithm is based on diffusion on the interactome [36]. We adapted their algorithm to prioritize the diseases in the viral disease network. This diffusion-based flow algorithm assigns a prioritization score to each node in the host interactome,

$$F(v) = \alpha \left[\sum_{u \in N(v)} F(u) w'(v,u) \right] + (1-\alpha) Y(v)$$

where w' is a normalized weight function representing the reliability of each edge in the network,

$$W'_{ij} = W_{ij} / \sqrt{D(i,i)D(j,j)}$$

The weight normalization takes into account the probability of observing a random edge between two proteins. That is, the weight of an edge is normalized by the degrees of its end-point, which is the probability of observing an edge between two nodes in a random network with the same node degrees. $Y(\nu)$ stores the prior information known about node ν; α, the only parameter in the algorithm, determines the relative importance of these constraints with respect to one another. For our data, the viral targets had their prior score set to 1, and W_{ij} for all edges equals 1.

The algorithm prioritizes genes in the host interactome. By turning to gene-disease associations we could prioritize the candidate diseases. If a disease has multiple known genes, as is often true, then each disease is assigned the highest score among the scores of its associated genes. To measure the precision-recall performance of the prioritization, we set the positive reference set to be the virally implicated diseases (Table 1). For a given cutoff score, precision was the number of true positives divided by all positives and recall was the number of true positives divided by all true positives. Setting α=0.7, or 0.8 or 0.9 all yielded similar results (Supplementary Figure S9A,B). We also calculated the relative risk of diseases above a certain cutoff score using the HPV and EBV patient data. We show results for when we included and excluded the positive reference set of virally implicated diseases from the averaging. Setting α=0.7 for EBV and α=0.8 for HPV yielded the best results (Supplementary Figure S9C,D). Lists of

diseases sorted according to the prediction score are given (Supplementary File S4). When we alternatively use the logarithm of relative risk, higher-ranked diseases in the prioritization are again more often associated with viral patients (Supplementary Figure S9E).

We measured the correlation between the relative risk of a disease and the rank of its associated gene (based on the average score obtained by the flow algorithm) using Pearson correlation coefficient (PCC) in Supplementary File S4. For EBV, PCC = 0.45 over the entire set of diseases (a total of 487 diseases). The top 100 diseases have a slightly higher correlation value of PCC=0.5. For HPV, the entire set appears not be correlated due to some outlying diseases with high relative risk with low scores. The top 100 diseases had a reasonable correlation value of PCC=0.30 and the top 25 have a PCC of 0.41. For both viruses, there are quite a few diseases whose genes are ranked low, but they have high relative risk. We believe this trend is likely due to genes that are rather disconnected from the interactome, but potentially may be associated with HPV. As interactome gets more complete, these effects may become less pronounced.

We also measured the precision-recall performance with random viral targets for control purposes (Supplementary Figure S10A,B). The curve we obtained is flat indicative of the poor performance of random targets in ranking virally implicated diseases highly. The relative risk of diseases above a certain cutoff score (Supplementary Figure S10C,D) is also flat with RR ~ 1, again indicating the poor performance of random targets in identifying diseases relevant to EBV and HPV patients.

Criteria to select potential virally implicated diseases

To assess if the diseases we identified might be virally implicated, we established three lines of evidence: (i) the disease has significant comorbidity with viral-associated diseases; (ii) the disease-associated genes in the disease network are differentially expressed in viral protein-induced cell populations; (iii) the disease-associated genes are differentially expressed in appropriate diseased tissues.

To obtain the disease associated genes that are differentially expressed in viral protein induced cell populations (criterion ii), HPV16 E6 and E7 oncogenes were independently transduced into primary human fibroblast (IMR90) (data from Rozenblatt-Rosen, et al) and keratinocyte (Ker) cell populations (this work). Affymetrix Human Gene1.0 ST and Human Genome U133 Plus 2.0 arrays, respectively, were used to measure gene expression profiles for five or more replicate samples in each of the cell types. Array data were normalized by RMA, batch effects were removed using ComBat, and the limma package in R/Bioconductor was used to identify differential expression. We find that of the 104 human genes that are regulated by 15 human protein targets of E6 and E7, 22 disease-associated genes were differentially expressed in the IMR90 (reproduced from Rozenblatt-Rosen, et al and provided herein for clarity in Supplementary Table S14) and keratinocyte cell lines (Supplementary Table S14). Of these 22 genes, 15 of them (*CDK4, CHEK1, CYC1, DDB2, DUSP1, FOSL1, IFITM1, ISG15, IVL, KRT16, MMP1, MMP2, NDRG1, ODC1, WEE1*) overlap with the genes differentially expressed in cervical carcinoma tissues (Supplementary Table S10c). There are 2,888 differentially expressed genes in the presence of E6 or E7 in IMR90 and keratinocyte cell lines out of 21,500 genes on the

microarray. The significance of finding 22 out 104 indirect viral targets differentially expressed in the presence of E6 or E7 is 0.0083 calculated by the hypergeometric distribution.

These 22 genes, differentially expressed in E6 or E7 induced IMR90 and/or HFK cell populations, have been linked to 39 diseases in OMIM, among which only six belong to known HPV-related diseases (Table 1B). We therefore asked if any of the remaining 33 diseases might be virally implicated (Figure 3F). 7 diseases out of 39 have high relative risk among HPV patients (four of which are previously unknown). To calculate the significance of having 7 out 39 to have high relative risk among HPV patients, we first calculated the number of diseases in the OMIM disease space that has significant prevalence among HPV patients. There are 626 OMIM diseases that have known genes associated with them as previously provided in Supplementary File S2. Of the 626 diseases, 38 of them have significant relative risk among HPV patients (Supplementary Table 17). Hence the significance of having 7 diseases among 39 calculated by hypergeometric distribution (MS Excel; =HYPGEOMDIST(7,39,38,626)) is 0.0049.

Of the four diseases that satisfy criteria (i) and (ii) above, for the three of them we could not validate criterion (iii), either due to lack of expression data on disease tissues (neoplasm of peritoneum), or lack of differential expression of the disease gene in the corresponding diseased tissue (benign neoplasm of skin) or the expression pattern induced by E6/E7 expression was the opposite from the one seen in the disease (diseases of sebaceous glands). The fourth disease on the list, Fanconi anemia, satisfied all three criteria. Previous studies have shown that HPV16 E7 induces expression of the FANCD2 gene through an E2F dependent

pathway. As this regulatory link from E2F1 to FANCD2 is absent in the literature-curated protein-DNA interaction list we used, this path did not emerge in our initial analysis. Once we make the E2F1→FANCD2 link explicit, we can confirm this path, as E7 interacts with E2F1 (Figure 3F) and in HPV16 E7 expressing IMR90 cells FANCD2 is differentially expressed along with other members of the nuclear multiprotein core complex (FANCA, FANCE, FANCG, FANCI, and FANCM) necessary for the monoubiquitination of FANCD2 [37].

Tissue specificity

To make our predictions more specific to the tissue types the viruses infect, we used tissue specific expression data from http://biogps.org to narrow down the number of genes and their associated diseases from the disease network. That is, if a gene in the neighborhood of the viral targets is not expressed in the tissue of interest, this gene is removed from the neighborhood. A number of considerations had to be made here about which tissue to choose and what cutoff to introduce as we decide whether a gene is expressed in that particular tissue. We used the criterion similar to the one used previously [38] by Su et al, where an average difference (AD) value of 200 was defined as a conservative threshold to call a gene ‘‘expressed’’ or present. Of the available tissues, we chose testis and ovary tissues for HPV and Burkitt’s lymphoma (Raji cells) and B lymphoblast tissues for EBV. With the tissue specificity consideration, the number of associated diseases for EBV went down from 128 to 89, and for HPV from 141 to 105, without losing any of the virally-implicated diseases (Supplementary Tables S15, S16). One can

include protein expression or better tissue expression data as it becomes available to make the predictions specific to the cell tropism of the virus as described here.

EBV presence in disease tissues

Despite a very strong correlation between EBV and Burkitt's lymphoma (BL), the contribution that EBV proteins make to BL remains to be fully elucidated. There has been some evidence for the detection of EBNA2 by immunohistology in endemic BL tumor cells [39]. Another paper looked at an unusually heterogenous BL sample [40] and reported finding cells in three different types of EBV latency, one of which expressed EBNA2 (but not LMP1). The EBNA2 positive cells were more resistant to apoptosis than cells in latency I "classical BL". Given this sparse evidence, EBNA2 may not make a significant contribution to ongoing BL. It is more likely that EBNA2 targeting of *MYC* may be an important precursor to BL. EBV most likely provides an anti-apoptotic rather than growth transforming effect.

When we refer to B-cell lymphoma, we do not consider post-transplant lymphoproliferative disease due to the fact that this disease occurs mainly due to immune deficiency hence we have no known genes associated with it. Mutations on *BCL2* and *BCL3* gene have been associated with many types of lymphomas, such as Hodgkin's lymphoma and follicular lymphoma [41,42]. EBV is present in many lymphoma tissues including Hodgkin's and non-Hodgkin lymphomas [43]. EBNA2 targeting RBPJ (which regulates *BCL3*) is fairly well understood and is a likely precursor to B-cell lymphoma [44].

There is some evidence of EBV being present in HCC cells [45,46], but the reproducibility of these findings has been questioned. Similarly lymphoepithelioma-like carcinoma, a form of lung cancer is strongly associated with EBV infection in Asian patients, but there is controversy over whether an association exists in patients from Western countries [47,48]. Our method aims to highlight potential molecular relationships between EBV and these diseases, but not necessarily directly causal relationships.

Topological properties of viral targets and viral disease networks

Viral proteins of HIV and EBV tend to target hub proteins [5,49]. This tendency held true for EBV and HPV16 datasets when either literature-curated interactions were included or omitted (Supplementary Figure S11).

Giant component size of the disease network. In addition to measuring the size of the giant component in the viral disease networks in Figure 3 B,D for random viral targets, we measured the size with randomized viral targets with degree-control. As shown in Supp Fig 8c and d, when the degree of the viral targets is preserved, the mean size of the largest connected component in viral disease network is larger than the non-controlled case. Meanwhile the true size of the EBV and HPV is significantly larger than the degree-controlled case (EBV $P = 0.00156$; HPV $P < 0.0001$).

Disease gene properties. In the main text, we calculated the shortest path lengths, defined as the minimum number of “hops” along the links of the host interactome from viral targets to

genes associated with a given virally implicated disease (Figure 1B). For either EBV or HPV the average shortest path (averaged over the number of virally implicated diseases) is significantly shorter than when virally implicated diseases were replaced with randomly sampled human diseases in OMIM. To test whether this result could be due to the properties of the genes associated with virally implicated diseases, we randomized the gene-disease associations by preserving their degree in the interactome. While this randomization will preserve the degree of disease genes, it will alter the gene that associated with virally implicated disease, hence altering the property of the original virally-implicated disease gene. This in turn would make the shortest path from viral targets to virally associated diseases much longer. For EBV, the distribution of random shortest paths shifted towards slightly longer shortest paths indicating that the genes associated with EBV-implicated diseases do have some central role (mean shortest path increased from 1.7 to 2.2; Supplementary Figure S12A). For HPV, the distribution of shortest paths remained the same as Figure 1D (mean shortest path remained 2.0; Supplementary Figure S12B).

List of supplementary files

Supplementary File S1. Full list of interactions and gene-disease associations in viral disease networks, including the sources of data. (**Sheet 1**) EBV disease network, (**Sheet 2**) HPV16 disease network. VH-PPI: virus-host protein-protein interaction, PPI: host protein-protein interaction, PDI: host protein-DNA interaction, MCI: metabolic enzyme-coupled interactions calculated using KEGG, or BIGG databases, or flux coupling analysis.

Supplementary File S2. OMIM genes and diseases and their corresponding ICD-9 codes.

Supplementary File S3. Relative risk analysis. **(Sheet 1, 3)** relative risk between EBV- and HPV-implicated diseases and candidate diseases in the disease network. **(Sheet 2, 4**) relative risk between EBV- and HPV-implicated diseases and all mappable diseases which constitute the random control.

Supplementary File S4. Full list of diseases prioritized by the flow algorithm for (**Sheet 1**) EBV (**Sheet 2**) HPV. Diseases are sorted according to maximum scores.

Supplementary figures and tables

Supplementary Table S1. EBV-human protein-protein and protein-DNA interactions curated from an authoritative Herpesvirus source [50], VirusMINT and previous reports.

Supplementary Table S2. HPV16-human protein-protein interactions curated from VirusMINT.

Supplementary Figure S1. Defining the neighborhood of viral targets. P, PT, PM, P^2 represent different possible network configurations.

Supplementary Figure S2. EBV average shortest path with **A**, literature-curated PPIs and PDIs are included **B,** Literature-curated PPIs removed from the virus-host interactions and host interactome.

Supplementary Figure S3. HPV average shortest path with **A,** literature-curated PPIs and PDIs are included **B**, Literature-curated PPIs removed from the virus-host interactions and host interactome.

Supplementary Figure S4. Analysis of viral disease network configurations. **A,B,** with both high-throughput and literature-curated virus-host interaction datasets and host PPIs. **C,D** without the literature-curated virus-host interactions and PPIs.

Supplementary Table S3. Configuration analysis for the EBV disease network, **A,** with literature-curated virus-host interactions and literature-curated host PPIs included (values are plotted in Supplementary Figure S4A), **B,** without literature-curated virus-host interactions and literature-curated host PPIs (Supplementary Figure S4C).

Supplementary Table S4. Configuration analysis for the HPV disease network. **A,** literature-curated virus-host interactions and literature-curated host PPIs included (values are plotted in Supplementary Figure S4B); **B,** without literature-curated virus-host interactions and literature-curated host PPIs (Supplementary Figure S4D).

Supplementary Figure S5. Transcriptional enrichment in disease tissues. **A,** B cell lymphoma cells [13] (P = 0.0018). **B,** head and neck squamous cell carcinoma cells [51] (P = 0.0037).

Supplementary Figure S6. Transcriptional enrichment in the viral disease network measured via gene expression analysis in tissues of selected EBV- and HPV-implicated diseases. M: number of differentially expressed genes in the disease tissue, V: number of genes regulated by virus-targeted transcription factors. The overlap in the Venn diagram indicates the observed number of differentially expressed genes in the viral disease network (the expected number is shown in parenthesis; * indicates significance; BL $P = 6.36\times10^{-5}$, BCL P = 0.00317, CC P = 0.000205, HNSCC P = 0.0025.

Supplementary Table S5. Number of differentially expressed genes in tissues of selected EBV and HPV virally implicated diseases and random control.

Supplementary Figure S7. Virally implicated diseases associated with genes in the neighborhoods of viral targets for HPV16 when p53-cervical cancer association is included in the OMIM Morbid Map.

Supplementary Table S6. Prioritized EBV candidate diseases.

Supplementary Table S7. Prioritized HPV candidate diseases.

Supplementary Figure S8. Size of giant component in random disease networks. **A,** EBV. **B,** HPV.

Supplementary Figure S9. Prioritization of predicted diseases in the EBV and HPV disease networks. **A,** EBV and **B**, HPV precision versus recall graphs at various values of alpha. **C,** EBV and **D,** HPV average relative risk of diseases as a function of rank. The averages are also calculated by omitting the virally implicated diseases. **E,** Average of the logarithm of relative risk for EBV (alpha = 0.7) and HPV (alpha = 0.8) as a function of rank.

Supplementary Figure S10. Prioritization obtained from random viral targets. Precision versus recall graphs obtained by random **A,** EBV and **B,** HPV targets. The average relative risk of diseases predicted via random **C,** EBV and **D**, HPV targets as a function of rank.

Supplementary Table S8. Comprehensive EBV-human protein-protein interaction dataset, including the source of data. Sources: CCSB (data from Rozenblatt-Rosen, et al, provided herein for clarity), Calderwood *et a l*[5], VirusMINT [6] and other.

Supplementary Table S9. Comprehensive HPV-human protein-protein interaction dataset, including the source of data. Sources: CCSB (data from Rozenblatt-Rosen, et al, provided herein for clarity), Huh *et al* [7], and VirusMINT [6].

Supplementary Table S10. List of genes that show significant expression change in **A**, Burkitt's lymphoma, **B**, B cell lymphoma, **C,** cervical cancer, and **D**, head and neck squamous cell carcinoma.

Supplementary Table S11. Raw data for calculating relative risks of candidate diseases in each viral disease network (PT configuration) among female HPV patients.

Supplementary Table S12. Raw data for calculating relative risks of candidate diseases in each viral disease network (PT configuration) among male HPV patients.

Supplementary Table S13. Raw data for calculating relative risks of candidate diseases in each viral disease network (PT configuration) among EBV patients.

Supplementary Table S14. Genes in the network vicinity of viral targets that are differentially expressed in gene expression profiles of normal human diploid fibroblast (IMR90 data from Rozenblatt-Rosen, et al, provided herein for clarity) and primary human keratinocyte (Ker) populations with stable expression of HPV16 E6 or E7 oncoproteins. Known HPV-implicated diseases are highlighted in red.

Supplementary Table S15. Tissue specificity analysis of EBV. Genes present and expressed in Burkitt's lymphoma (Raji cells) and B lymphocyte tissues are shown based on the GNF1H dataset in http://biogps.org with their expression values and their associated diseases.

Supplementary Table S16. Tissue specificity analysis of HPV16. Genes present and expressed in ovary and testis tissues are shown based on the GNF1H dataset in http://biogps.org with their expression values and their associated diseases.

Supplementary Table S17. OMIM diseases that have significant prevalence among HPV patients. Diseases in bold are in the HPV disease network (Figure 3C).

Supplementary Figures and Tables

Supplementary Table S1. EBV-human protein-protein and protein-DNA interactions curated from an authoritative Herpesvirus source [50], VirusMINT and previous reports.

Source	Viral protein	Human protein	Type
**VirusMINT**	EBNA1	CSNK2A1	PPI
**VirusMINT**	EBNA1	CSNK2A2	PPI
**VirusMINT**	EBNA1	EBNA1BP2	PPI
**VirusMINT**	EBNA1	KPNA1	PPI
**VirusMINT**	EBNA1	NAP1L1	PPI
**VirusMINT**	EBNA1	ORC2L	PPI
**VirusMINT**	EBNA1	SFRS1	PPI
**VirusMINT**	EBNA1	TERF2	PPI
**VirusMINT|ISBN:0521827140**	EBNA1	USP7	PPI
**VirusMINT|ISBN:0521827140**	LMP1	TRADD	PPI
**VirusMINT|ISBN:0521827140**	LMP1	ZMYND11	PPI
**VirusMINT**	LMP1	RABAC1	PPI
**ISBN:0521827140**	EBNA1	RPA1	PPI
**ISBN:0521827140**	EBNA1	TNPO1	PPI
**ISBN:0521827140**	EBNA2	DDX20	PPI
**ISBN:0521827140**	EBNA2	EP300	PPI
**ISBN:0521827140**	EBNA2	GTF2B	PPI
**ISBN:0521827140**	EBNA2	GTF2E1	PPI
**ISBN:0521827140**	EBNA2	GTF2E2	PPI
**ISBN:0521827140**	EBNA2	GTF2H4	PPI
**ISBN:0521827140**	EBNA2	MYB	PPI
**ISBN:0521827140**	EBNA2	SMARCB1	PPI
**ISBN:0521827140**	EBNA2	SND1	PPI
**ISBN:0521827140**	EBNA2	SPI1	PPI
**ISBN:0521827140**	EBNA2	SPIB	PPI
**ISBN:0521827140**	EBNA2	TBP	PPI
**ISBN:0521827140**	EBNA3	AHR	PPI
**ISBN:0521827140**	EBNA3	AIP	PPI
**ISBN:0521827140**	EBNA3	CCT5	PPI
**ISBN:0521827140**	EBNA3	CTBP1	PPI
**ISBN:0521827140**	EBNA3	UPRT	PPI
**ISBN:0521827140**	EBNA6	DDX20	PPI
**ISBN:0521827140**	EBNA6	NME1	PPI
**ISBN:0521827140**	EBNA6	PTMA	PPI
**ISBN:0521827140**	EBNA6	RBPJ	PPI
**ISBN:0521827140**	EBNA6	SMN1	PPI

**ISBN:0521827140**	EBNALP	AKAP8L	PPI
**ISBN:0521827140**	EBNALP	CDKN2A	PPI
**ISBN:0521827140**	EBNALP	HAX1	PPI
**ISBN:0521827140**	EBNALP	P4HA1	PPI
**ISBN:0521827140**	EBNALP	RPS3A	PPI
**ISBN:0521827140**	LMP1	TRAF1	PPI
**ISBN:0521827140**	LMP1	TRAF2	PPI
**ISBN:0521827140**	LMP1	TRAF3	PPI
**ISBN:0521827140**	LMP2A	ITCH	PPI
**ISBN:0521827140**	LMP2A	LCK	PPI
**ISBN:0521827140**	LMP2A	LYN	PPI
**ISBN:0521827140**	LMP2A	NEDD4	PPI
**ISBN:0521827140**	LMP2A	SRC	PPI
**ISBN:0521827140**	LMP2A	SYK	PPI
**ISBN:0521827140**	LMP2A	ZAP70	PPI
**PMID:18039120|ISBN:0521827140**	EBNA2	RBPJ	PPI
**VirusMINT|ISBN:0521827140**	EBNA1	USP7	PPI
**VirusMINT|ISBN:0521827140**	LMP1	TRADD	PPI
**VirusMINT|ISBN:0521827140**	LMP1	ZMYND11	PPI
**PMID:10725330**	EB1/BZLF1	UBN1	PPI
**PMID:12195020**	EBNA2	NR4A1	PPI
**PMID:7784177**	EB1/BZLF1	RARA	PPI
**PMID:7784177**	EB1/BZLF1	RXRA	PPI
**PMID:8114725**	EB1/BZLF1	NFKB1	PPI
**PMID:8390666**	EBNALP	RB1	PPI
**PMID:8390666**	EBNALP	TP53	PPI
**PMID:12438576|PMID:8114724**	EB1/BZLF1	TP53	PDI & PPI
**PMID:18039120**	EBNA2	CR2	PDI
**PMID:18039120**	EBNA2	FCER2	PDI
**PMID:18039120**	EBNA2	FGR	PDI
**PMID:18039120**	EBNA2	MYC	PDI
**PMID:7520093**	LMP1	BCL2	PDI

Supplementary Table S2. HPV16-human protein-protein curated from VirusMINT.

Viral Protein	Host Protein	Source of interaction
**E1_HPV16**	TRIP13	VirusMINT
**E1_HPV16**	UBE2I	VirusMINT
**E2_HPV16**	BRD4	VirusMINT
**E2_HPV16**	CEBPA	VirusMINT
**E2_HPV16**	CEBPB	VirusMINT
**E2_HPV16**	CITED1	VirusMINT
**E2_HPV16**	GTF2B	VirusMINT
**E2_HPV16**	KAT2B	VirusMINT
**E2_HPV16**	POMP	VirusMINT
**E2_HPV16**	SMN2	VirusMINT
**E2_HPV16**	TBP	VirusMINT
**E2_HPV16**	TOPBP1	VirusMINT
**E2_HPV16**	TP53	VirusMINT
**E4_HPV16**	SRPK1	VirusMINT
**E5_HPV16**	HLA-A	VirusMINT
**E6_HPV16**	BAK1	VirusMINT
**E6_HPV16**	BRCA1	VirusMINT
**E6_HPV16**	CREBBP	VirusMINT
**E6_HPV16**	DLG1	VirusMINT
**E6_HPV16**	EP300	VirusMINT
**E6_HPV16**	FADD	VirusMINT
**E6_HPV16**	FBLN1	VirusMINT
**E6_HPV16**	GPS2	VirusMINT
**E6_HPV16**	IRF3	VirusMINT
**E6_HPV16**	KPNA2	VirusMINT
**E6_HPV16**	KPNB1	VirusMINT
**E6_HPV16**	MAGI1	VirusMINT
**E6_HPV16**	MCM7	VirusMINT
**E6_HPV16**	MYC	VirusMINT
**E6_HPV16**	PTPN3	VirusMINT
**E6_HPV16**	RCN2	VirusMINT
**E6_HPV16**	SCRIB	VirusMINT
**E6_HPV16**	SERTAD1	VirusMINT
**E6_HPV16**	SIPA1L1	VirusMINT
**E6_HPV16**	TAX1BP3	VirusMINT
**E6_HPV16**	TNFRSF1A	VirusMINT
**E6_HPV16**	TNPO1	VirusMINT
**E6_HPV16**	TP53	VirusMINT
**E6_HPV16**	TP73	VirusMINT
**E6_HPV16**	TSC2	VirusMINT

**E6_HPV16**	UBE3A	VirusMINT
**E6_HPV16**	XRCC1	VirusMINT
**E7_HPV16**	AKAP8	Huh et al.
**E7_HPV16**	AKAP8L	Huh et al.
**E7_HPV16**	ALPL	Huh et al.
**E7_HPV16**	BRCA1	VirusMINT
**E7_HPV16**	CAD	Huh et al.
**E7_HPV16**	CBX4	Huh et al.
**E7_HPV16**	CCNA2	VirusMINT/Huh et al.
**E7_HPV16**	CCNE2	VirusMINT/Huh et al.
**E7_HPV16**	CCT2	Huh et al.
**E7_HPV16**	CCT4	Huh et al.
**E7_HPV16**	CCT8	Huh et al.
**E7_HPV16**	CDC2	VirusMINT/Huh et al.
**E7_HPV16**	CDK2	VirusMINT/Huh et al.
**E7_HPV16**	CDKN1A	VirusMINT
**E7_HPV16**	CHD4	VirusMINT
**E7_HPV16**	COPA	Huh et al.
**E7_HPV16**	CREBBP	VirusMINT
**E7_HPV16**	CTTN	Huh et al.
**E7_HPV16**	CUL2	Huh et al.
**E7_HPV16**	DDX17	Huh et al.
**E7_HPV16**	DNAJA1	Huh et al.
**E7_HPV16**	DNAJA3	VirusMINT
**E7_HPV16**	E2F1	Huh et al.|VirusMINT
**E7_HPV16**	E2F2	Huh et al.|VirusMINT
**E7_HPV16**	E2F3	Huh et al.|VirusMINT
**E7_HPV16**	E2F4	Huh et al.|VirusMINT
**E7_HPV16**	E2F5	Huh et al.
**E7_HPV16**	E2F6	Huh et al.
**E7_HPV16**	E7_HPV16	Huh et al.
**E7_HPV16**	EIF3F	Huh et al.
**E7_HPV16**	EIF3I	Huh et al.
**E7_HPV16**	EIF4A1	Huh et al.
**E7_HPV16**	EIF4B	Huh et al.
**E7_HPV16**	EP300	VirusMINT
**E7_HPV16**	FLNA	Huh et al.
**E7_HPV16**	FOS	VirusMINT
**E7_HPV16**	FOXM1	VirusMINT
**E7_HPV16**	FUS	Huh et al.
**E7_HPV16**	GATAD2B	Huh et al.
**E7_HPV16**	HDAC1	VirusMINT
**E7_HPV16**	HDAC2	VirusMINT
**E7_HPV16**	HNRNPC	Huh et al.

**E7_HPV16**	HNRNPE2	Huh et al.
**E7_HPV16**	HNRNPF	Huh et al.
**E7_HPV16**	HNRNPG	Huh et al.
**E7_HPV16**	HNRNPH1	Huh et al.
**E7_HPV16**	HNRNPK	Huh et al.
**E7_HPV16**	HNRNPM	Huh et al.
**E7_HPV16**	HSPA5	Huh et al.
**E7_HPV16**	HSPA8	Huh et al.
**E7_HPV16**	IQGAP1	Huh et al.
**E7_HPV16**	IRF1	VirusMINT
**E7_HPV16**	JUN	VirusMINT
**E7_HPV16**	JUNB	VirusMINT
**E7_HPV16**	JUND	VirusMINT
**E7_HPV16**	KAT2B	VirusMINT
**E7_HPV16**	KCMF1	Huh et al.
**E7_HPV16**	KIAA1949	Huh et al.
**E7_HPV16**	KIAA1967	Huh et al.
**E7_HPV16**	L3MBTL2	Huh et al.
**E7_HPV16**	MAP7	Huh et al.
**E7_HPV16**	MATR3	Huh et al.
**E7_HPV16**	MGA	Huh et al.
**E7_HPV16**	MTA2	Huh et al.
**E7_HPV16**	MYC	VirusMINT
**E7_HPV16**	NCOA1	Huh et al.
**E7_HPV16**	NFIL3	Huh et al.
**E7_HPV16**	NOLC1	VirusMINT
**E7_HPV16**	NUMA1	Huh et al.
**E7_HPV16**	PCGF6	Huh et al.
**E7_HPV16**	PFKL	Huh et al.
**E7_HPV16**	PHC3	Huh et al.
**E7_HPV16**	PHGDH	Huh et al.
**E7_HPV16**	PKM2	VirusMINT
**E7_HPV16**	PLCB3	Huh et al.
**E7_HPV16**	POU5F1	VirusMINT
**E7_HPV16**	PPP1CA	Huh et al.
**E7_HPV16**	PPP2CA	VirusMINT
**E7_HPV16**	PPP2R1A	VirusMINT
**E7_HPV16**	PRMT5	Huh et al.
**E7_HPV16**	PRPH	Huh et al.
**E7_HPV16**	PSMC1	VirusMINT
**E7_HPV16**	RAN	VirusMINT
**E7_HPV16**	RB1	Huh et al.|VirusMINT
**E7_HPV16**	RBBP7	Huh et al.
**E7_HPV16**	RBL1	VirusMINT/Huh et al.

**E7_HPV16**	RBL2	Huh et al.|VirusMINT
**E7_HPV16**	RCOR1	Huh et al.
**E7_HPV16**	RFC2	Huh et al.
**E7_HPV16**	RIF1	Huh et al.
**E7_HPV16**	RING1	Huh et al.
**E7_HPV16**	RNF2	Huh et al.
**E7_HPV16**	RPS25	VirusMINT
**E7_HPV16**	RUVBL2	Huh et al.
**E7_HPV16**	SAP130	Huh et al.
**E7_HPV16**	SAP155	Huh et al.
**E7_HPV16**	SIVA1	VirusMINT
**E7_HPV16**	SNW1	VirusMINT
**E7_HPV16**	SQSTM1	Huh et al.
**E7_HPV16**	STK38	Huh et al.
**E7_HPV16**	TAF15	Huh et al.
**E7_HPV16**	TARDBP	Huh et al.
**E7_HPV16**	TBL1XR1	Huh et al.
**E7_HPV16**	TBL1Y	Huh et al.
**E7_HPV16**	TBP	VirusMINT
**E7_HPV16**	TFDP1	VirusMINT/Huh et al.
**E7_HPV16**	TFDP2	VirusMINT/Huh et al.
**E7_HPV16**	TRIP13	Huh et al.
**E7_HPV16**	TUBA1C	Huh et al.
**E7_HPV16**	TUBA3C	Huh et al.
**E7_HPV16**	TUBA4A	Huh et al.
**E7_HPV16**	TUBB2A	Huh et al.
**E7_HPV16**	UBR4	Huh et al.
**E7_HPV16**	UBR5	Huh et al.
**E7_HPV16**	VIM	Huh et al.
**E7_HPV16**	WDR5	Huh et al.
**E7_HPV16**	WDR77	Huh et al.
**E7_HPV16**	ZER1	Huh et al.
**L1_HPV16**	IPO5	VirusMINT
**L1_HPV16**	KPNA2	VirusMINT
**L1_HPV16**	KPNB1	VirusMINT
**L1_HPV16**	TNPO1	VirusMINT
**L2_HPV16**	GADD45GIP1	VirusMINT
**L2_HPV16**	IPO5	VirusMINT
**L2_HPV16**	KPNA2	VirusMINT
**L2_HPV16**	KPNB1	VirusMINT
**L2_HPV16**	MAN2B1	VirusMINT
**L2_HPV16**	PATZ1	VirusMINT
**L2_HPV16**	TINAGL1	VirusMINT
**L2_HPV16**	TNPO1	VirusMINT

Figure S1. **Defining the neighborhood of viral targets**. P, PT, PM, P^2 represent different possible network configurations.

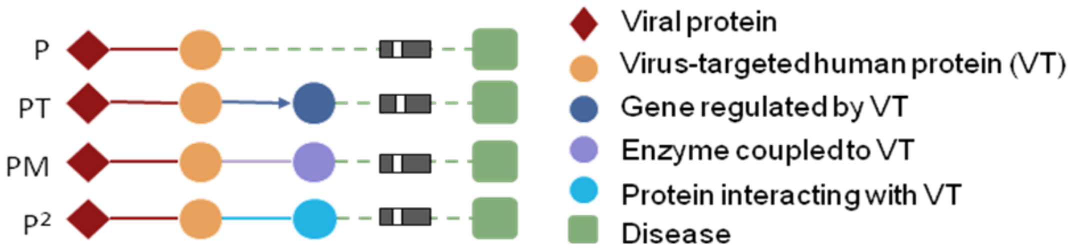

Supplementary Figure S2. EBV average shortest path. **A**, literature-curated PPIs and PDIs are included in the host interactome, and **B,** Literature-curated PPIs are removed, but PDIs are included.

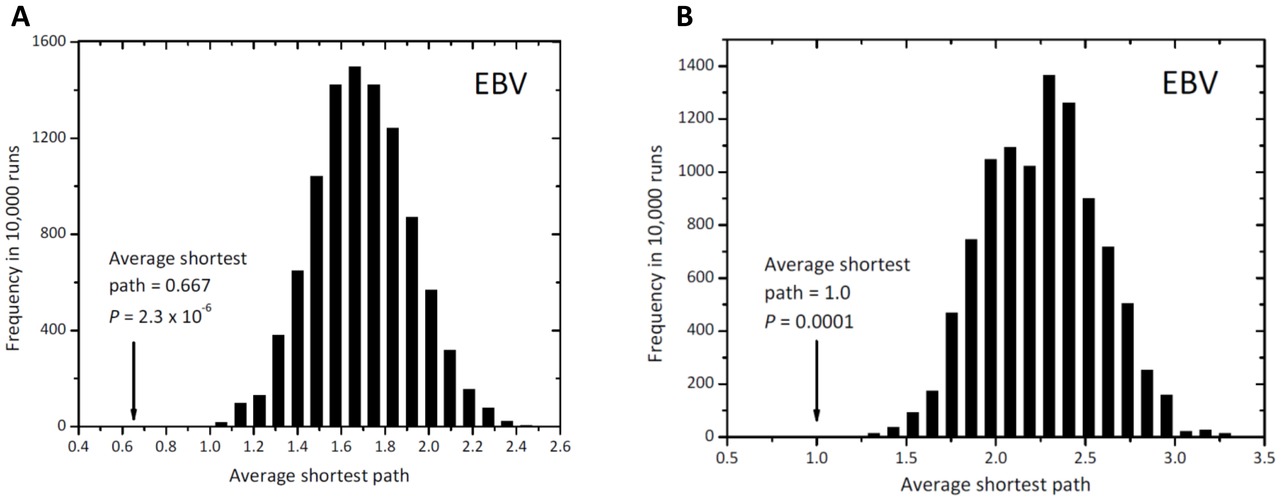

Supplementary Figure S3. HPV16 average shortest path. A, Literature-curated PPIs and PDIs are included in the host interactome, and **B,** Literature-curated PPIs are removed, but PDIs are included.

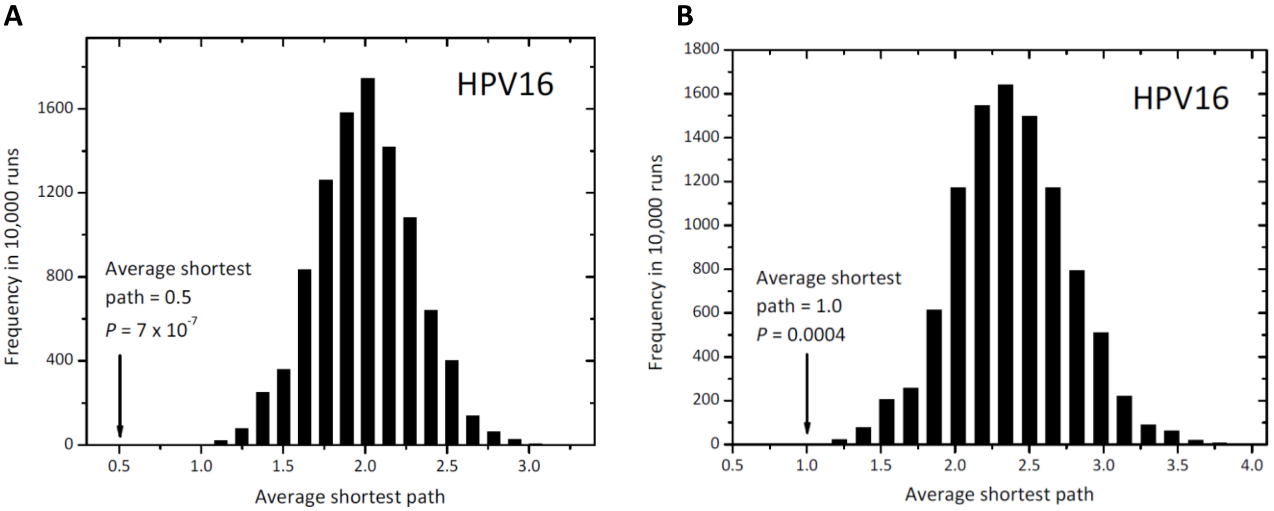

Supplementary Figure S4. Analysis of viral disease network configurations. **A,B,** with both high-throughput and literature-curated virus-host interaction datasets and host PPIs. **C,D,** without the literature-curated virus-host interactions and PPIs.

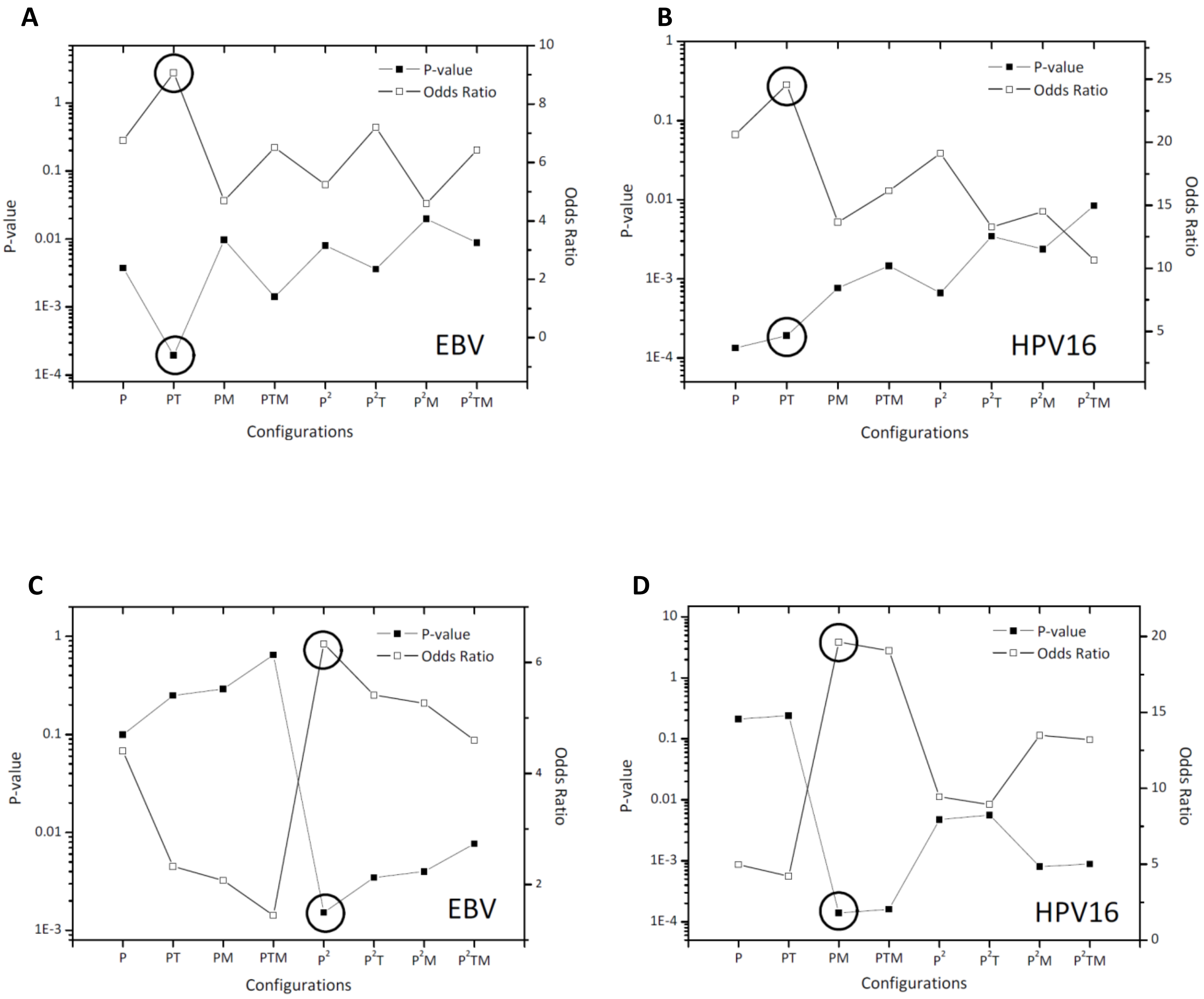

Supplementary Table S3. Configuration analysis for the EBV viral disease network **A**, with literature curated virus-host interactions (Supplementary Figure S4A). **B** without literature-curated virus-host interactions and literature-curated PPI (values are plotted in Supplementary Figure S4C).

A

Virus	Configurations	Virally implicated diseases	All diseases	P-value	Odds ratio
**EBV**	P	5	56	3.75E-03	6.75
**EBV**	PT	9	128	1.94E-04	9.068
**EBV**	PM	6	99	0.0097	4.692
**EBV**	PTM	9	163	0.0014	6.507
**EBV**	P^2	10	243	0.0080	5.237
**EBV**	P^2T	11	270	0.0036	7.199
**EBV**	P^2M	10	262	0.0197	4.592
**EBV**	P^2TM	11	287	0.0087	6.42

B

Virus	Configurations	Virally implicated diseases	All diseases	P-value	Odds ratio
**EBV**	P	2	26	0.099	4.404
**EBV**	PT	2	46	0.2494	2.326
**EBV**	PM	2	51	0.2899	2.071
**EBV**	PTM	2	70	0.6491	1.443
**EBV**	P^2	8	129	0.0015	6.334
**EBV**	P^2T	8	145	0.0035	5.411
**EBV**	P^2M	8	148	0.004	5.262
**EBV**	P^2TM	8	163	0.0077	4.6

Supplementary Table S4. Configuration analysis for the HPV16 viral disease network. **A,** with literature-curated virus-host interactions (Supplementary Figure S4B). **B,** without literature-curated virus-host interactions (values are plotted in Supplementary Figure S4D).

A

Virus	Configurations	Virally implicated diseases	All diseases	P-value	Odds ratio
**HPV16**	P	5	50	1.34E-04	20.62
**HPV16**	PT	7	141	0.00019	24.56
**HPV16**	PM	6	115	0.00076	13.65
**HPV16**	PTM	7	190	0.00146	16.15
**HPV16**	P^2	7	169	0.00066	19.13
**HPV16**	P^2T	7	216	0.00346	13.28
**HPV16**	P^2M	7	204	0.00236	14.51
**HPV16**	P^2TM	7	247	0.00838	10.67

B

Virus	Configurations	Virally implicated diseases	All diseases	P-value	Odds ratio
**HPV16**	P	1	18	0.2107	4.981
**HPV16**	PT	1	21	0.2418	4.216
**HPV16**	PM	6	86	0.00014	19.63
**HPV16**	PTM	6	88	0.00016	19.08
**HPV16**	P^2	4	62	0.00474	9.447
**HPV16**	P^2T	4	65	0.00566	8.937
**HPV16**	P^2M	6	116	0.00080	13.5
**HPV16**	P^2TM	6	118	0.00088	13.21

Supplementary Figure S5. Transcriptional enrichment in disease tissues.

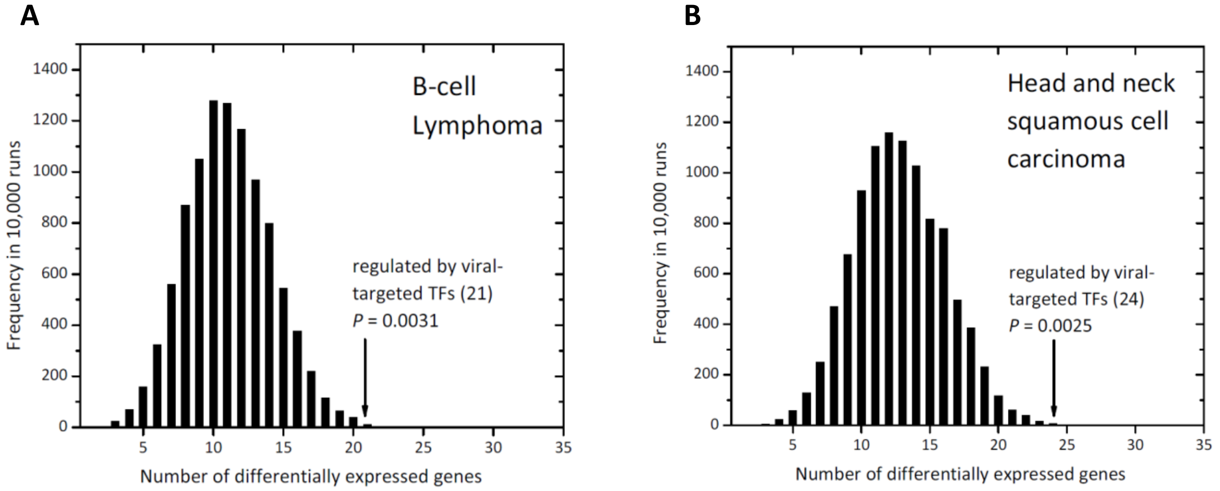

Supplementary Figure S6. Transcriptional enrichment in the viral disease network measured via gene expression analysis in tissues of selected EBV- and HPV- implicated diseases. M: number of differentially expressed genes in the disease tissue, V: number of genes regulated by virus-targeted transcription factors. The overlap in the Venn diagram indicates the observed number of differentially expressed genes in the viral disease network (the expected number is shown in parenthesis; * indicates significance; p-values reported in the text).

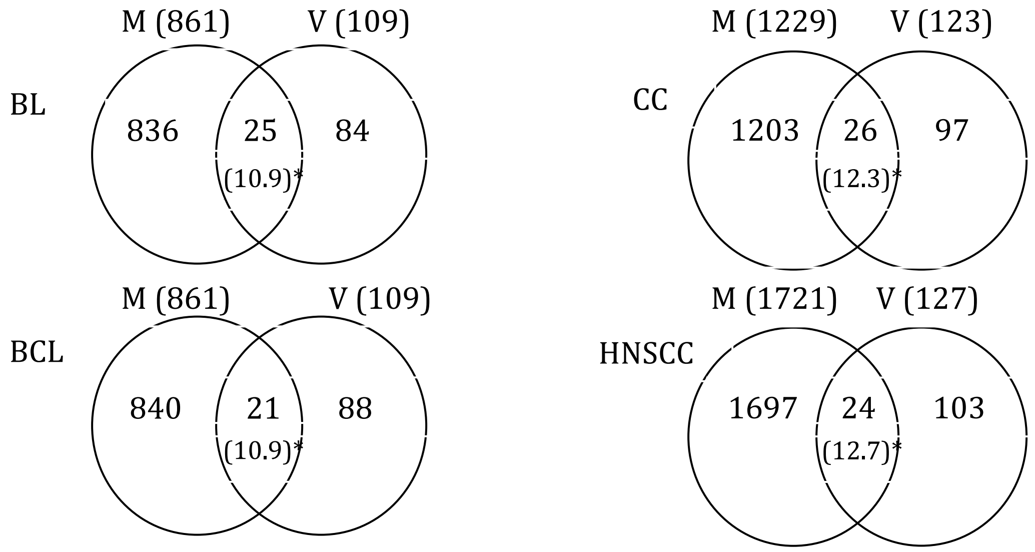

Supplementary Table S5: Number of differentially expressed genes in tissues of selected EBV and HPV virally implicated diseases and random control by choosing different cutoffs in gene expression analysis.

Viral disease network	Genes regulate d by VTs	Diseases	Cutof f	Genes regulated by VTs and significantly up/down-regulated (Overlap)	Overlap in random viral disease network (average)
EBV	109	Burkitt's lymphoma	2%<br>5%<br>10%	12<br>25<br>36	4.36<br>10.9<br>21.8
		B cell lymphoma	2%<br>5%<br>10%	11<br>21<br>40	4.36<br>10.9<br>21.8
HPV16	123	Cervical Cancer	2%<br>5%<br>10%	12<br>26<br>41	4.92<br>12.3<br>24.6
		HNSCC	2%<br>5%<br>10%	13<br>24<br>31	4.92<br>12.3<br>24.6

Supplementary Figure S7. Distribution of number of virally implicated diseases hit by random HPV neighborhoods when p53-cervical cancer association is included in the OMIM Morbid Map.

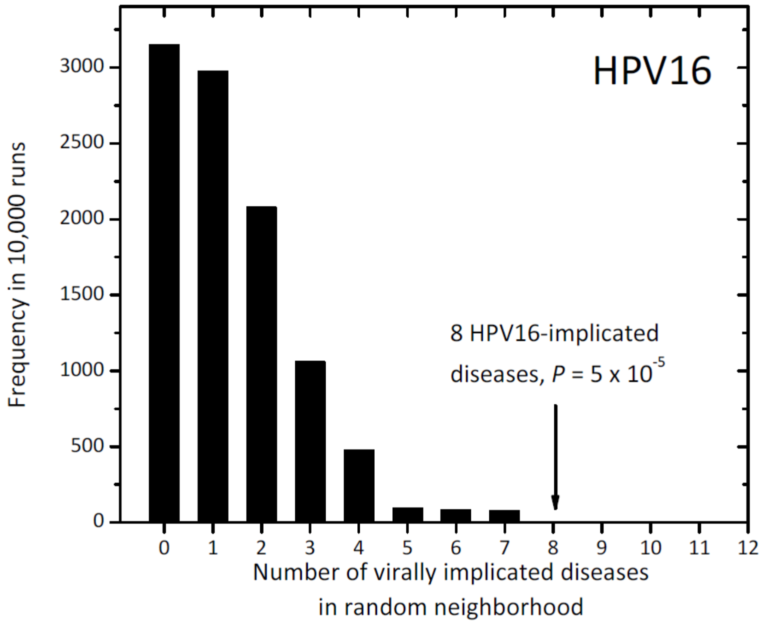

Supplementary Figure S8. Size of the giant component in the random viral disease networks **A**, EBV and **B**, HPV. The arrows indicate the real viral disease network size (EBV size = 253, HPV size =220)

A

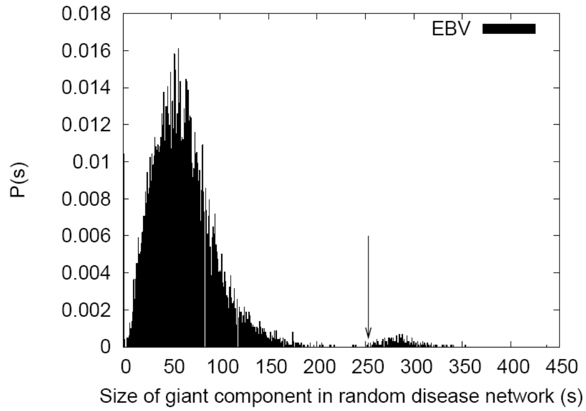

B

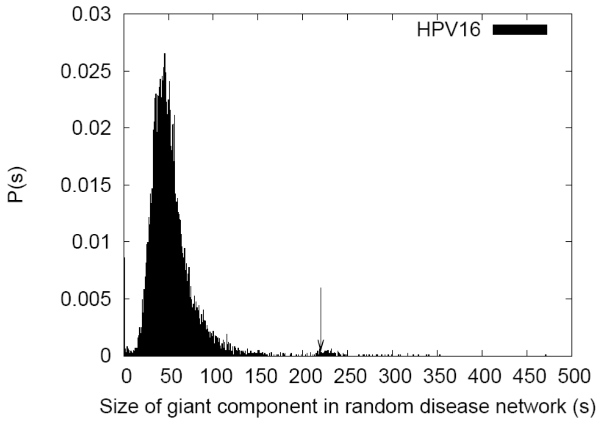

C

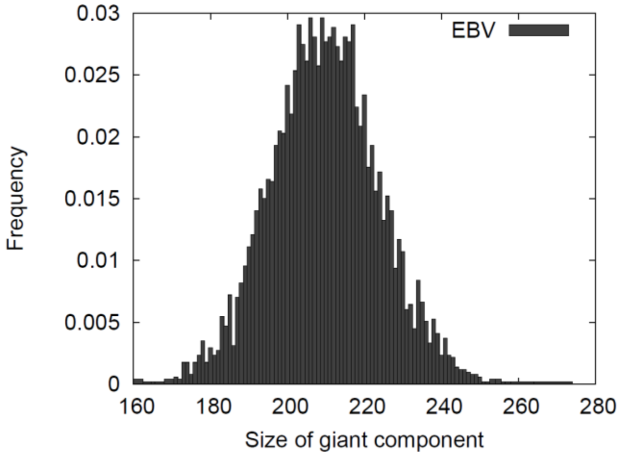

D

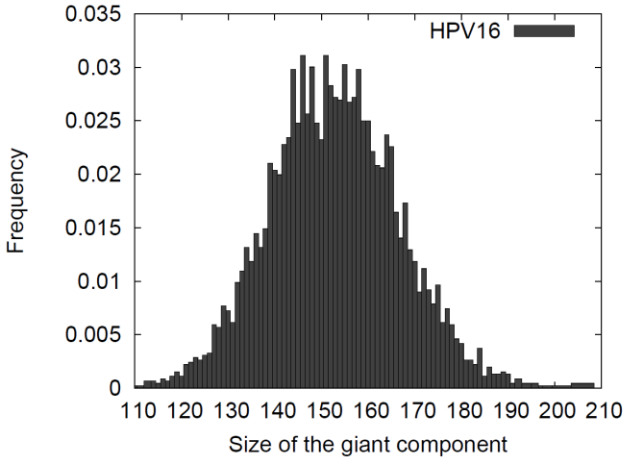
HPV16
Frequency
0.035
0.03
0.025
0.02
0.015
0.01
0.005
0
110 120 130 140 150 160 170 180 190 200 210
Size of the giant component

Supplementary Figure S9. Prioritization of candidate diseases in the EBV and HPV disease networks. Precision versus recall for various values of alpha for **A**, EBV and **B**, HPV. Average relative risk of diseases as a function of rank are shown for **C,** EBV and **D**, HPV. The averages are also calculated by omitting the virally implicated diseases (GSPs in the prioritization algorithm). **E**, Average of the logarithm of relative risk is shown for EBV (alpha=0.7) and HPV (alpha=0.8) as a function of rank.

A

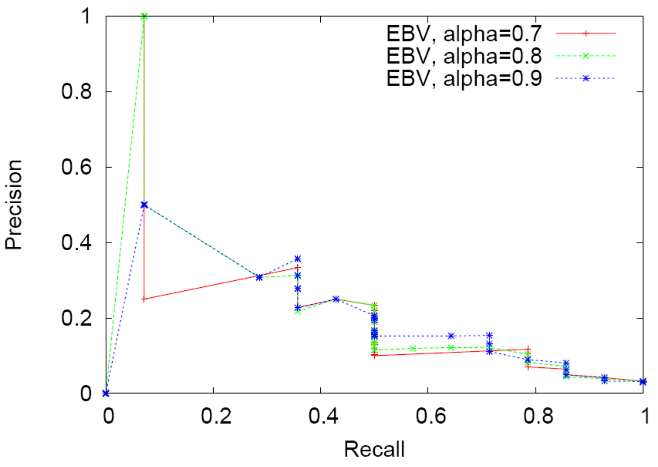

B

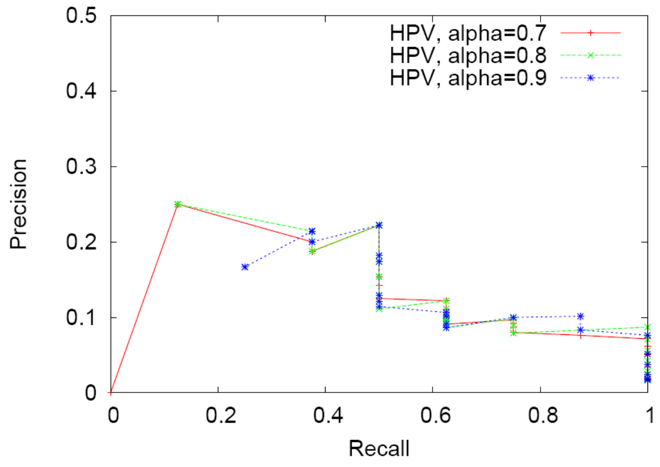

C

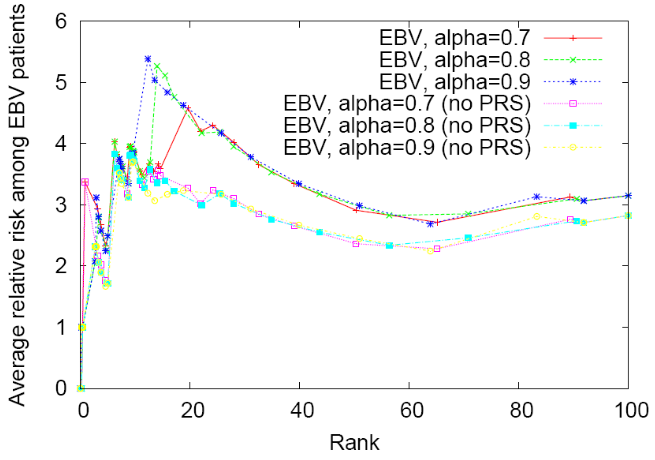

D

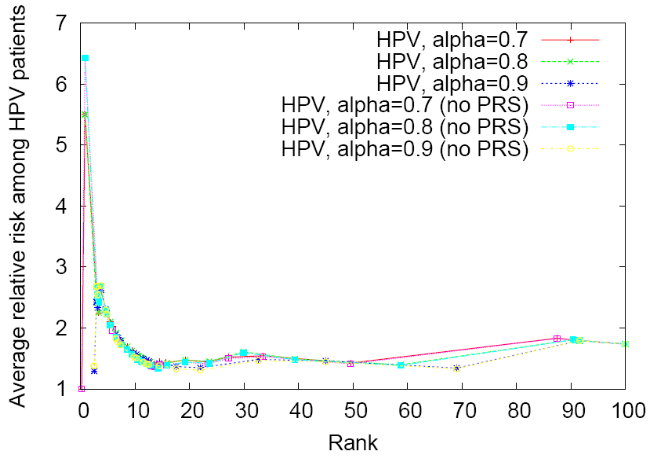

E

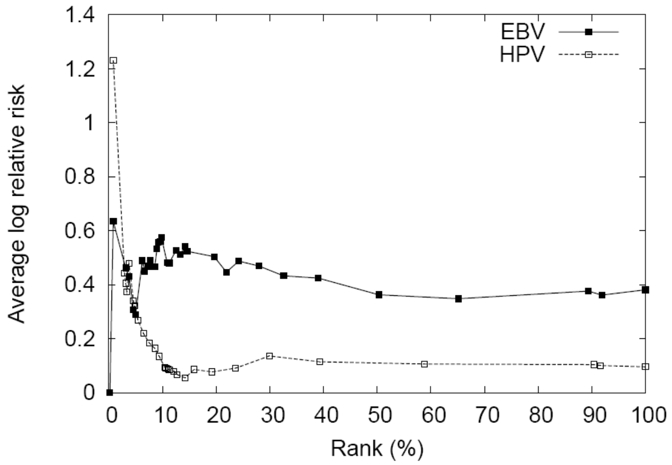

Supplementary Figure S10. Prioritization obtained from random viral targets.

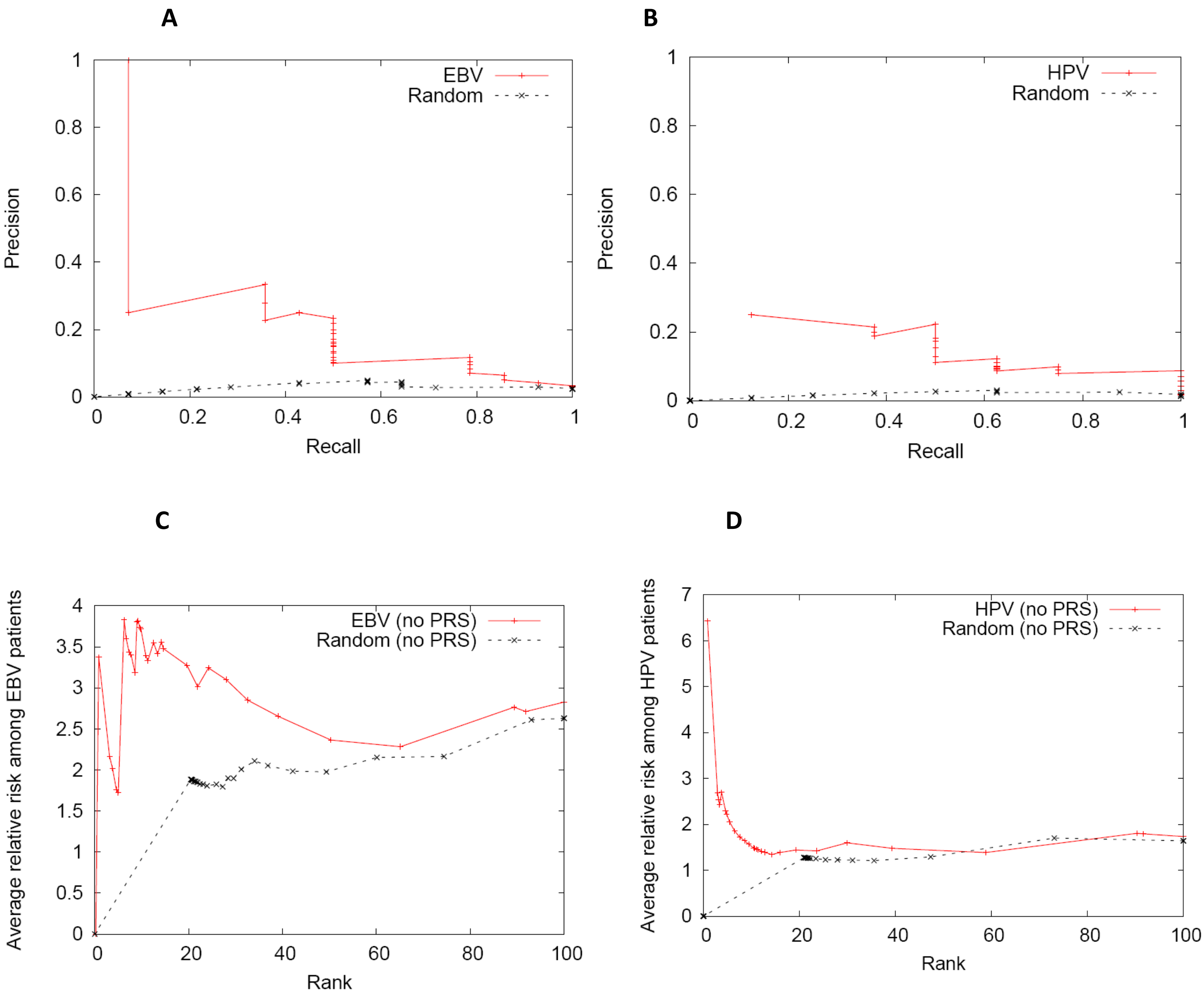

Supplementary Table S6. Prioritized EBV disease candidates according to prioritization score derived from flow algorithm. Marked diseases (*) are virally implicated diseases.

ICD-9 code	RR in EBV patients	ICD-9 description	Max score
200.2*	522.10	Burkitt's tumor or lymphoma	0.597
190.5	1.00	Malignant neoplasm of eye, Retina	0.497
170.9	8.32	Malignant neoplasm of bone and articular cartilage	0.497
188.9	0.81	Malignant neoplasm of bladder	0.497
208.9*	14.58	Leukemia/lymphoma	0.467
147.9*	522.10	Malignant neoplasm of nasopharynx	0.465
216.9	1.94	Benign neoplasm of skin	0.465
259.2	1.00	Other endocrine disorders, Carcinoid syndrome	0.465
V84.01	1.00	Genetic susceptibility to malignant neoplasm of breast	0.465
157.8	1.00	Malignant neoplasm of pancreas	0.465
194	2.80	Malignant neoplasm of other endocrine glands and related structures	0.465
211.5*	1.00	Benign neoplasm of liver	0.465
239.3*	1.00	Neoplasms of unspecified nature, Breast	0.465
193	2.80	Malignant neoplasm of thyroid gland	0.465
153	1.00	Malignant neoplasm of colon	0.465
569.89	2.54	Other specified disorders of intestine	0.430
356.1	2.61	Peroneal muscular atrophy	0.416
286.4	1.00	von Willebrand's disease	0.414
272.6	1.00	Lipodystrophy	0.412
333.4	1.00	Huntington's chorea	0.412
334.3	1.00	Spinocerebellar disease	0.412
332	0.69	Parkinson's disease	0.412

Supplementary Table S7. Prioritized HPV disease candidates according to prioritization score derived from flow algorithm. Marked diseases (*) are virally implicated diseases.

ICD-9 code	RR in female HPV patients	ICD-9 description	Max score
277.3	1.000	Amyloidosis	0.508
190.5	15.738	Malignant neoplasm of eye, Retina	0.373
188.9*	2.724	Malignant neoplasm of bladder	0.373
170.9	2.550	Malignant neoplasm of bone and articular cartilage	0.373
V84.01	1.000	Genetic susceptibility to malignant neoplasm of breast	0.356
259.2	1.000	Other endocrine disorders, Carcinoid syndrome	0.356
239.3*	1.000	Neoplasms of unspecified nature, Breast	0.356
216.9	3.216	Benign neoplasm of skin	0.356
211.5	1.000	Benign neoplasm of liver and biliary passages	0.356
194	1.000	Malignant neoplasm of other endocrine glands	0.356
193	1.000	Malignant neoplasm of thyroid gland	0.356
157.8	1.000	Malignant neoplasm of pancreas	0.356
153*	0.754	Malignant neoplasm of colon	0.356
147.9	1.000	Malignant neoplasm of nasopharynx	0.356
759.89	1.000	Other and unspecified congenital anomalies	0.327
183*	5.003	Malignant neoplasm of ovary and other uterine adnexa	0.301
158.8	6.217	Malignant neoplasm of retroperitoneum and peritoneum	0.301
368.5	1.000	Visual disturbances	0.279
208.9	1.000	Leukemia/lymphoma	0.279
334.3	1.000	Spinocerebellar disease	0.276
333.4	1.000	Huntington's chorea	0.276
332	0.565	Parkinson's disease	0.276

Supplementary Table S8. Comprehensive EBV-human protein-protein interaction dataset, including the source of data. Sources: CCSB (data from Rozenblatt-Rosen, et al, provided herein for clarity), Calderwood *et al* [5], VirusMINT [6] and other.

Viral Protein	Host Protein	Source of interaction
BALF1	CEP76	CCSB
BALF1	FHL3	CCSB
BALF2	GOLGA2	CCSB
BALF2	NEFL	CCSB
BALF2	PNMA1	CCSB
BALF2	ZYX	CCSB
BBRF2	C1orf50	CCSB
BBRF2	C6orf182	CCSB
BBRF2	CEP76	CCSB
BBRF2	COPB1	CCSB
BBRF2	DZIP3	CCSB
BBRF2	GOLGA2	CCSB
BBRF2	GSTA4	CCSB
BBRF2	MCM6	CCSB
BBRF2	MIPOL1	CCSB
BBRF2	NEFL	CCSB
BBRF2	OPTN	CCSB
BBRF2	PNMA1	CCSB
BBRF2	PTBP1	CCSB
BBRF2	RINT1	CCSB
BBRF2	TFCP2	CCSB
BDLF2	CEP76	CCSB
BDLF3	AGR2	CCSB
BDLF3	OGT	CCSB
BFLF2	GOLGA2	CCSB
BFLF2	GPRASP2	CCSB
BFLF2	KIAA0774	CCSB
BFLF2	KIFC3	CCSB
BFLF2	LZTS2	CCSB
BFLF2	MDFI	CCSB
BFLF2	PDE4DIP	CCSB
BFLF2	PNMA1	CCSB
BFLF2	TFIP11	CCSB
BFLF2	TRAF1	CCSB
BFLF2	TRIM27	CCSB
BFRF1	CRTAC1	CCSB
BFRF1	ZBED1	CCSB
BHRF1	BIK	CCSB
BHRF1	ERLIN1	CCSB

BHRF1	EXOC5	CCSB
BHRF1	FATE1	CCSB
BHRF1	MARCO	CCSB
BHRF1	NAALADL2	CCSB
BLRF2	DVL2	CCSB
BNLF2A	CREB3	CCSB
BNLF2A	RAB3IP	CCSB
BNLF2A	UBE2I	CCSB
BNLF2B	CCNDBP1	CCSB
BNLF2B	SPAG5	CCSB
BPLF1	AP2B1	CCSB
BSLF2;BMLF1	BEND7	CCSB
BSLF2;BMLF1	COIL	CCSB
BSLF2;BMLF1	KHDRBS2	CCSB
BSLF2;BMLF1	PSME3	CCSB
BSLF2;BMLF1	RBMX	CCSB
BSLF2;BMLF1	RBMY1F	CCSB
BSLF2;BMLF1	RBPMS	CCSB
BSLF2;BMLF1	TFCP2	CCSB
BSLF2;BMLF1	UBE2I	CCSB
BZLF2	CREB3	CCSB
DUT	TAX1BP1	CCSB
DUT	UBE2I	CCSB
EB1/BZLF1	C1orf94	CCSB
EB1/BZLF1	HSFY1	CCSB
EB1/BZLF1	PEX5	CCSB
EB1/BZLF1	POGZ	CCSB
EB1/BZLF1	RBPMS	CCSB
EB1/BZLF1	TFG	CCSB
EBNA1	KPNA3	CCSB
EBNA1	KPNA6	CCSB
EBNA1	LNX1	CCSB
EBNA1	SRPK2	CCSB
EBNA3	RFX6	CCSB
EBNA4	BANP	CCSB
EBNA4	ESRRG	CCSB
EBNA4	GMCL1	CCSB
EBNA4	GOLGA2	CCSB
EBNA4	PSME3	CCSB
EBNA4	RBPMS	CCSB
EBNA4	RIMBP3	CCSB
EBNA4	TFCP2	CCSB
EBNA4	TRAF4	CCSB
EBNA6	MAGED1	CCSB

EBNA6	RBPMS	CCSB
EBNALP	CCDC67	CCSB
EBNALP	ESRRG	CCSB
EBNALP	MAGEA1	CCSB
EBNALP	SCYL1	CCSB
EBNALP	SP100	CCSB
GB	CCNDBP1	CCSB
GB	DVL2	CCSB
GB	ESRRG	CCSB
GB	GOLGA2	CCSB
GB	MID2	CCSB
GB	MKRN3	CCSB
GB	PNMA1	CCSB
GB	RIMBP3	CCSB
GB	TFIP11	CCSB
GB	TRIM27	CCSB
GM	CCDC33	CCSB
GN	CREB3	CCSB
GN	TMEM159	CCSB
LF2	JAKMIP2	CCSB
LF2	LNX1	CCSB
LF2	NDC80	CCSB
LMP1	UBQLN1	CCSB
LMP2A	LNX1	CCSB
TK	TFCP2	CCSB
UNG	GSTA4	CCSB
BDLF1	CCDC14	CCSB|Calderwood
BDLF2	PSMA3	CCSB|Calderwood
BFRF1	HOMER3	CCSB|Calderwood
EBNA4	PSMA3	CCSB|Calderwood
GB	LZTS2	CCSB|Calderwood
EBNA3	RBPJ	CCSB|ISBN:0521827140
EBNA4	RBPJ	CCSB|ISBN:0521827140
BALF2	ELF2	Calderwood
BALF2	FBLN5	Calderwood
BARF1	ACTN1	Calderwood
BARF1	PKM2	Calderwood
BARF1	SERTAD1	Calderwood
BARF1	TMEM66	Calderwood
BBRF2	ARHGEF10L	Calderwood
BBRF2	PICK1	Calderwood
BDLF1	CTSC	Calderwood
BDLF1	MAPK7	Calderwood
BDLF2	APOL3	Calderwood

BDLF2	LTBP4	Calderwood
BDLF2	PSME3	Calderwood
BDLF2	TES	Calderwood
BDLF3	HOMER3	Calderwood
BDLF3.5	SERTAD1	Calderwood
BDLF4	MFSD1	Calderwood
BDLF4	PIGS	Calderwood
BFLF2	ACTN1	Calderwood
BFLF2	BAT3	Calderwood
BFLF2	CASKIN2	Calderwood
BFLF2	DKK3	Calderwood
BFLF2	EFEMP1	Calderwood
BFLF2	EFEMP2	Calderwood
BFLF2	EGFL7	Calderwood
BFLF2	FBLN2	Calderwood
BFLF2	FBLN5	Calderwood
BFLF2	GRN	Calderwood
BFLF2	HOMER3	Calderwood
BFLF2	LAMB2	Calderwood
BFLF2	LTBP4	Calderwood
BFLF2	MARCO	Calderwood
BFLF2	NUCB1	Calderwood
BFLF2	PLSCR1	Calderwood
BFLF2	RBPMS	Calderwood
BFLF2	TRAF3IP3	Calderwood
BFLF2	TSNARE1	Calderwood
BFLF2	TXNDC11	Calderwood
BFLF2	UBE2I	Calderwood
BFRF1	GRN	Calderwood
BFRF1	PRMT1	Calderwood
BFRF1	SLIT3	Calderwood
BFRF1	VWF	Calderwood
BGLF2	HOMER3	Calderwood
BGLF2	OPTN	Calderwood
BGLF2	RBCK1	Calderwood
BGLF2	SHROOM3	Calderwood
BGLF2	TUBA1A	Calderwood
BGLF4	IQGAP2	Calderwood
BGRF1/BDRF1	ENOX2	Calderwood
BGRF1/BDRF1	GPRASP1	Calderwood
BGRF1/BDRF1	GPRASP2	Calderwood
BGRF1/BDRF1	TSC22D4	Calderwood
BHRF1	ACTN1	Calderwood
BHRF1	FN1	Calderwood

BHRF1	GRN	Calderwood
BHRF1	LTBP4	Calderwood
BHRF1	TNXB	Calderwood
BHRF1	TRAF1	Calderwood
BHRF1	VWF	Calderwood
BILF1	GRN	Calderwood
BILF1	HOMER3	Calderwood
BILF1	LMNB1	Calderwood
BILF1	SLIT2	Calderwood
BILF1	VWF	Calderwood
BLLF1	HOMER3	Calderwood
BLLF2	GRN	Calderwood
BNLF2B	GRN	Calderwood
BOLF1	PSMA3	Calderwood
BOLF1	SERTAD1	Calderwood
BPLF1	CCHCR1	Calderwood
BPLF1	CD74	Calderwood
BPLF1	CIR	Calderwood
BPLF1	CLK1	Calderwood
BPLF1	GAPDH	Calderwood
BPLF1	GRN	Calderwood
BPLF1	HBB	Calderwood
BPLF1	HLA-A	Calderwood
BPLF1	HLA-B	Calderwood
BPLF1	IGLL1	Calderwood
BPLF1	LASS4	Calderwood
BPLF1	NUCB2	Calderwood
BPLF1	RPL3	Calderwood
BRRF1	EFEMP1	Calderwood
BRRF1	FBLN5	Calderwood
BRRF1	LZTS2	Calderwood
BRRF1	NTN4	Calderwood
BRRF1	PARP4	Calderwood
BRRF1	PHYHD1	Calderwood
BRRF1	PLSCR1	Calderwood
BRRF1	SEPP1	Calderwood
BRRF1	SH3GLB1	Calderwood
BRRF1	SNX4	Calderwood
BRRF1	TRAF2	Calderwood
BSLF1	NFKB1	Calderwood
BTRF1	PPP1CA	Calderwood
BVRF1	ACTN1	Calderwood
BVRF1	EFEMP1	Calderwood
BVRF1	EFEMP2	Calderwood

BVRF1	IMMT	Calderwood
BVRF1	MMRN2	Calderwood
BVRF1	NCKIPSD	Calderwood
BVRF1	PKM2	Calderwood
BVRF1	PSMA3	Calderwood
BVRF1	PSME3	Calderwood
BVRF1	RNF31	Calderwood
BVRF1	TSG101	Calderwood
BXRF1	FBLN5	Calderwood
BZLF2	GRN	Calderwood
EB1/BZLF1	ENOX2	Calderwood
EB1/BZLF1	GPRASP1	Calderwood
EBNA1	MDFI	Calderwood
EBNA1	PICK1	Calderwood
EBNA1	TSG101	Calderwood
EBNA2	AES	Calderwood
EBNA2	BLNK	Calderwood
EBNA2	CBX3	Calderwood
EBNA2	CD46	Calderwood
EBNA2	CD5L	Calderwood
EBNA2	DYNLL1	Calderwood
EBNA2	EFEMP2	Calderwood
EBNA2	FBLN5	Calderwood
EBNA2	FCHSD1	Calderwood
EBNA2	GCA	Calderwood
EBNA2	KRT36	Calderwood
EBNA2	PSMA3	Calderwood
EBNA2	RBP1	Calderwood
EBNA2	RCBTB1	Calderwood
EBNA2	SH3GLB1	Calderwood
EBNA2	SRI	Calderwood
EBNA3	NR4A1	Calderwood
EBNA3	RPL4	Calderwood
EBNA4	ACTN1	Calderwood
EBNA4	ACTN4	Calderwood
EBNA4	SH3GLB1	Calderwood
EBNA4	ZYX	Calderwood
EBNA6	FBLN5	Calderwood
EBNA6	PSMA3	Calderwood
EBNA6	SEPP1	Calderwood
EBNA6	VWF	Calderwood
EBNALP	PSMA3	Calderwood
GB	AP2B1	Calderwood
GB	FAM168B	Calderwood

GB	FBLN5	Calderwood
GB	FN1	Calderwood
GB	GPRASP1	Calderwood
GB	GRN	Calderwood
GB	LAMB1	Calderwood
GB	LTBP4	Calderwood
GB	PDE6G	Calderwood
GB	TRAF2	Calderwood
GB	UBXN11	Calderwood
GB	VIM	Calderwood
GB	VWF	Calderwood
GL	HOMER3	Calderwood
GM	HOMER3	Calderwood
GM	NUCB1	Calderwood
GN	HOMER3	Calderwood
LMP1	EGFL7	Calderwood
LMP2	LGALS3BP	Calderwood
LMP2A	LGALS3BP	Calderwood
SM	MAPRE1	Calderwood
SM	SP100	Calderwood
SM	TRA2B	Calderwood
TK	ENOX2	Calderwood
TK	GPRASP1	Calderwood
TK	GPRASP2	Calderwood
TK	TSC22D4	Calderwood
EBNA2	ZMYND11	Calderwood|VirusMINT
EBNA1	C1QBP	Calderwood|VirusMINT|ISBN:0521827140
EBNA1	KPNA2	Calderwood|VirusMINT|ISBN:0521827140
EBNA1	RPA1	ISBN:0521827140
EBNA1	TNPO1	ISBN:0521827140
EBNA2	DDX20	ISBN:0521827140
EBNA2	EP300	ISBN:0521827140
EBNA2	GTF2B	ISBN:0521827140
EBNA2	GTF2E1	ISBN:0521827140
EBNA2	GTF2E2	ISBN:0521827140
EBNA2	GTF2H4	ISBN:0521827140
EBNA2	MYB	ISBN:0521827140
EBNA2	SMARCB1	ISBN:0521827140
EBNA2	SND1	ISBN:0521827140
EBNA2	SPI1	ISBN:0521827140
EBNA2	SPIB	ISBN:0521827140
EBNA2	TBP	ISBN:0521827140
EBNA3	AHR	ISBN:0521827140
EBNA3	AIP	ISBN:0521827140

EBNA3	CCT5	ISBN:0521827140
EBNA3	CTBP1	ISBN:0521827140
EBNA3	UPRT	ISBN:0521827140
EBNA6	DDX20	ISBN:0521827140
EBNA6	NME1	ISBN:0521827140
EBNA6	PTMA	ISBN:0521827140
EBNA6	RBPJ	ISBN:0521827140
EBNA6	SMN1	ISBN:0521827140
EBNALP	CDKN2A	ISBN:0521827140
EBNALP	P4HA1	ISBN:0521827140
LMP1	TRAF1	ISBN:0521827140
LMP1	TRAF2	ISBN:0521827140
LMP1	TRAF3	ISBN:0521827140
LMP2A	ITCH	ISBN:0521827140
LMP2A	LCK	ISBN:0521827140
LMP2A	LYN	ISBN:0521827140
LMP2A	NEDD4	ISBN:0521827140
LMP2A	SRC	ISBN:0521827140
LMP2A	SYK	ISBN:0521827140
LMP2A	ZAP70	ISBN:0521827140
EB1/BZLF1	UBN1	PMID:10725330
EBNA2	NR4A1	PMID:12195020
EB1/BZLF1	TP53	PMID:12438576|PMID:8114724
EBNA2	RBPJ	PMID:18039120|ISBN:0521827140
EB1/BZLF1	RARA	PMID:7784177
EB1/BZLF1	RXRA	PMID:7784177
EB1/BZLF1	NFKB1	PMID:8114725
EBNALP	RB1	PMID:8390666
EBNALP	TP53	PMID:8390666
EBNA1	CSNK2A1	VirusMINT
EBNA1	CSNK2A2	VirusMINT
EBNA1	EBNA1BP2	VirusMINT
EBNA1	KPNA1	VirusMINT
EBNA1	NAP1L1	VirusMINT
EBNA1	ORC2L	VirusMINT
EBNA1	SFRS1	VirusMINT
EBNA1	TERF2	VirusMINT
EBNA6	CTBP1	VirusMINT
EBNALP	BAG2	VirusMINT
EBNALP	CAD	VirusMINT
EBNALP	CAPZA1	VirusMINT
EBNALP	CDC2	VirusMINT
EBNALP	CLPX	VirusMINT
EBNALP	CLTC	VirusMINT

EBNALP	CSE1L	VirusMINT
EBNALP	DARS	VirusMINT
EBNALP	DDX17	VirusMINT
EBNALP	DDX5	VirusMINT
EBNALP	DHX9	VirusMINT
EBNALP	DNAJB1	VirusMINT
EBNALP	DNAJB5	VirusMINT
EBNALP	DNAJB6	VirusMINT
EBNALP	EIF3B	VirusMINT
EBNALP	EPRS	VirusMINT
EBNALP	FLNC	VirusMINT
EBNALP	GBAS	VirusMINT
EBNALP	GCN1L1	VirusMINT
EBNALP	GLUD1	VirusMINT
EBNALP	GRPEL1	VirusMINT
EBNALP	H1FX	VirusMINT
EBNALP	HIST1H1D	VirusMINT
EBNALP	HIST1H2BI	VirusMINT
EBNALP	HNRNPA0	VirusMINT
EBNALP	HNRNPA1	VirusMINT
EBNALP	HNRNPA2B1	VirusMINT
EBNALP	HNRNPAB	VirusMINT
EBNALP	HNRNPCL1	VirusMINT
EBNALP	HNRNPD	VirusMINT
EBNALP	HNRNPK	VirusMINT
EBNALP	HNRNPM	VirusMINT
EBNALP	HNRNPR	VirusMINT
EBNALP	HNRNPU	VirusMINT
EBNALP	HSPA1B	VirusMINT
EBNALP	HSPA5	VirusMINT
EBNALP	HSPA8	VirusMINT
EBNALP	HSPB1	VirusMINT
EBNALP	HSPD1	VirusMINT
EBNALP	IARS	VirusMINT
EBNALP	ILF3	VirusMINT
EBNALP	IQGAP2	VirusMINT
EBNALP	KHDRBS1	VirusMINT
EBNALP	LDHB	VirusMINT
EBNALP	LRPPRC	VirusMINT
EBNALP	MAGED1	VirusMINT
EBNALP	MLF2	VirusMINT
EBNALP	NCL	VirusMINT
EBNALP	NIPSNAP1	VirusMINT
EBNALP	NONO	VirusMINT

EBNALP	NUP93	VirusMINT
EBNALP	P4HA2	VirusMINT
EBNALP	PCBP2	VirusMINT
EBNALP	PCCA	VirusMINT
EBNALP	PCCB	VirusMINT
EBNALP	PRPF8	VirusMINT
EBNALP	PSMB6	VirusMINT
EBNALP	PSMC2	VirusMINT
EBNALP	PSMD2	VirusMINT
EBNALP	PTBP1	VirusMINT
EBNALP	QARS	VirusMINT
EBNALP	RAD50	VirusMINT
EBNALP	RCN1	VirusMINT
EBNALP	RCN2	VirusMINT
EBNALP	RFC3	VirusMINT
EBNALP	RPL10	VirusMINT
EBNALP	RPL12	VirusMINT
EBNALP	RPL13	VirusMINT
EBNALP	RPL17	VirusMINT
EBNALP	RPL18	VirusMINT
EBNALP	RPL22	VirusMINT
EBNALP	RPL23	VirusMINT
EBNALP	RPL31	VirusMINT
EBNALP	RPL7	VirusMINT
EBNALP	RPL7A	VirusMINT
EBNALP	RPL8	VirusMINT
EBNALP	RPS13	VirusMINT
EBNALP	RPS16	VirusMINT
EBNALP	RPS17	VirusMINT
EBNALP	RPS18	VirusMINT
EBNALP	RPS2	VirusMINT
EBNALP	RPS23	VirusMINT
EBNALP	RPS6	VirusMINT
EBNALP	RPS8	VirusMINT
EBNALP	RPS9	VirusMINT
EBNALP	RSL1D1	VirusMINT
EBNALP	RUVBL1	VirusMINT
EBNALP	RUVBL2	VirusMINT
EBNALP	SEC13	VirusMINT
EBNALP	SEC16A	VirusMINT
EBNALP	SERBP1	VirusMINT
EBNALP	SET	VirusMINT
EBNALP	SF3B1	VirusMINT
EBNALP	SF3B2	VirusMINT

EBNALP	SFPQ	VirusMINT
EBNALP	SFRS1	VirusMINT
EBNALP	SLC25A6	VirusMINT
EBNALP	SNRNP200	VirusMINT
EBNALP	SNRPA	VirusMINT
EBNALP	SNRPB2	VirusMINT
EBNALP	SNRPD2	VirusMINT
EBNALP	SSBP1	VirusMINT
EBNALP	SYNCRIP	VirusMINT
EBNALP	TIMM50	VirusMINT
EBNALP	TNNT3	VirusMINT
EBNALP	TUBA1B	VirusMINT
EBNALP	TUBB	VirusMINT
EBNALP	UBC	VirusMINT
EBNALP	UBR5	VirusMINT
EBNALP	WDR77	VirusMINT
EBNALP	XPOT	VirusMINT
EBNALP	XRCC5	VirusMINT
LMP1	RABAC1	VirusMINT
EBNA1	USP7	VirusMINT|ISBN:0521827140
EBNALP	AKAP8L	VirusMINT|ISBN:0521827140
EBNALP	HAX1	VirusMINT|ISBN:0521827140
EBNALP	RPS3A	VirusMINT|ISBN:0521827140
LMP1	TRADD	VirusMINT|ISBN:0521827140
LMP1	ZMYND11	VirusMINT|ISBN:0521827140

Supplementary Table S9. Comprehensive HPV-human protein-protein interaction dataset, including the source of data. Sources: HPV16-human interactions. Sources: CCSB (data from Rozenblatt-Rosen, et al, provided herein for clarity), Huh *et a l*[7], and VirusMINT [6].

Viral Protein	Host Protein	Source of interaction
E6_HPV16	C9orf97	CCSB
E6_HPV16	QARS	CCSB
E6_HPV16	SIPA1L2	CCSB
E6_HPV16	ZNF417	CCSB
E7_HPV16	ATP5O	CCSB
E7_HPV16	BEND7	CCSB
E7_HPV16	BYSL	CCSB
E7_HPV16	C5orf55	CCSB

E7_HPV16	CCDC94	CCSB
E7_HPV16	CLK2	CCSB
E7_HPV16	FAM164C	CCSB
E7_HPV16	FAM90A1	CCSB
E7_HPV16	FARS2	CCSB
E7_HPV16	FUBP3	CCSB
E7_HPV16	INO80B	CCSB
E7_HPV16	LGALS14	CCSB
E7_HPV16	LMO2	CCSB
E7_HPV16	LMO4	CCSB
E7_HPV16	PARD6B	CCSB
E7_HPV16	PIN4	CCSB
E7_HPV16	POLR1C	CCSB
E7_HPV16	PRKAA2	CCSB
E7_HPV16	RBX1	CCSB
E7_HPV16	RPL9	CCSB
E7_HPV16	SCNM1	CCSB
E7_HPV16	SRP19	CCSB
E7_HPV16	SYT16	CCSB
E7_HPV16	TEAD4	CCSB
E7_HPV16	TXNDC5	CCSB
E7_HPV16	ZBTB25	CCSB
E7_HPV16	ZGPAT	CCSB
E7_HPV16	ZNF408	CCSB
E7_HPV16	ZNF417	CCSB
E7_HPV16	GATAD2B	CCSB|Huh et al.
E7_HPV16	AKAP8	Huh et al.
E7_HPV16	AKAP8L	Huh et al.
E7_HPV16	ALPL	Huh et al.
E7_HPV16	CAD	Huh et al.
E7_HPV16	CBX4	Huh et al.
E7_HPV16	CCT2	Huh et al.
E7_HPV16	CCT4	Huh et al.
E7_HPV16	CCT8	Huh et al.
E7_HPV16	COPA	Huh et al.
E7_HPV16	CTTN	Huh et al.
E7_HPV16	CUL2	Huh et al.
E7_HPV16	DDX17	Huh et al.
E7_HPV16	DNAJA1	Huh et al.
E7_HPV16	E2F5	Huh et al.
E7_HPV16	E2F6	Huh et al.
E7_HPV16	EIF3F	Huh et al.
E7_HPV16	EIF3I	Huh et al.
E7_HPV16	EIF4A1	Huh et al.

E7_HPV16	EIF4B	Huh et al.
E7_HPV16	FLNA	Huh et al.
E7_HPV16	FUS	Huh et al.
E7_HPV16	HNRNPC	Huh et al.
E7_HPV16	HNRNPF	Huh et al.
E7_HPV16	HNRNPH1	Huh et al.
E7_HPV16	HNRNPK	Huh et al.
E7_HPV16	HNRNPM	Huh et al.
E7_HPV16	HSPA5	Huh et al.
E7_HPV16	HSPA8	Huh et al.
E7_HPV16	IQGAP1	Huh et al.
E7_HPV16	KCMF1	Huh et al.
E7_HPV16	KIAA1949	Huh et al.
E7_HPV16	KIAA1967	Huh et al.
E7_HPV16	L3MBTL2	Huh et al.
E7_HPV16	MAP7	Huh et al.
E7_HPV16	MATR3	Huh et al.
E7_HPV16	MGA	Huh et al.
E7_HPV16	MTA2	Huh et al.
E7_HPV16	NCOA1	Huh et al.
E7_HPV16	NFIL3	Huh et al.
E7_HPV16	NUMA1	Huh et al.
E7_HPV16	PCBP2	Huh et al.
E7_HPV16	PCGF6	Huh et al.
E7_HPV16	PFKL	Huh et al.
E7_HPV16	PHC3	Huh et al.
E7_HPV16	PHGDH	Huh et al.
E7_HPV16	PLCB3	Huh et al.
E7_HPV16	PPP1CA	Huh et al.
E7_HPV16	PRMT5	Huh et al.
E7_HPV16	PRPH	Huh et al.
E7_HPV16	PYGM	Huh et al.
E7_HPV16	RBBP7	Huh et al.
E7_HPV16	RBMXL2	Huh et al.
E7_HPV16	RCOR1	Huh et al.
E7_HPV16	RFC2	Huh et al.
E7_HPV16	RIF1	Huh et al.
E7_HPV16	RING1	Huh et al.
E7_HPV16	RNF2	Huh et al.
E7_HPV16	RUVBL2	Huh et al.
E7_HPV16	SAP130	Huh et al.
E7_HPV16	SF3B1	Huh et al.
E7_HPV16	SQSTM1	Huh et al.
E7_HPV16	STK38	Huh et al.

E7_HPV16	TAF15	Huh et al.
E7_HPV16	TARDBP	Huh et al.
E7_HPV16	TBL1XR1	Huh et al.
E7_HPV16	TBL1Y	Huh et al.
E7_HPV16	TRIP13	Huh et al.
E7_HPV16	TUBA1C	Huh et al.
E7_HPV16	TUBA3C	Huh et al.
E7_HPV16	TUBA4A	Huh et al.
E7_HPV16	TUBB2A	Huh et al.
E7_HPV16	UBR4	Huh et al.
E7_HPV16	UBR5	Huh et al.
E7_HPV16	VIM	Huh et al.
E7_HPV16	WDR5	Huh et al.
E7_HPV16	WDR77	Huh et al.
E7_HPV16	ZER1	Huh et al.
E1_HPV16	TRIP13	VirusMINT
E1_HPV16	UBE2I	VirusMINT
E2_HPV16	BRD4	VirusMINT
E2_HPV16	CEBPA	VirusMINT
E2_HPV16	CEBPB	VirusMINT
E2_HPV16	CITED1	VirusMINT
E2_HPV16	GTF2B	VirusMINT
E2_HPV16	KAT2B	VirusMINT
E2_HPV16	POMP	VirusMINT
E2_HPV16	SMN2	VirusMINT
E2_HPV16	TBP	VirusMINT
E2_HPV16	TOPBP1	VirusMINT
E2_HPV16	TP53	VirusMINT
E4_HPV16	SRPK1	VirusMINT
E5_HPV16	HLA-A	VirusMINT
E6_HPV16	BAK1	VirusMINT
E6_HPV16	BRCA1	VirusMINT
E6_HPV16	CREBBP	VirusMINT
E6_HPV16	DLG1	VirusMINT
E6_HPV16	EP300	VirusMINT
E6_HPV16	FADD	VirusMINT
E6_HPV16	FBLN1	VirusMINT
E6_HPV16	GPS2	VirusMINT
E6_HPV16	IRF3	VirusMINT
E6_HPV16	KPNA2	VirusMINT
E6_HPV16	KPNB1	VirusMINT
E6_HPV16	MAGI1	VirusMINT
E6_HPV16	MCM7	VirusMINT
E6_HPV16	MYC	VirusMINT

E6_HPV16	PTPN3	VirusMINT
E6_HPV16	RCN2	VirusMINT
E6_HPV16	SCRIB	VirusMINT
E6_HPV16	SERTAD1	VirusMINT
E6_HPV16	SIPA1L1	VirusMINT
E6_HPV16	TAX1BP3	VirusMINT
E6_HPV16	TNFRSF1A	VirusMINT
E6_HPV16	TNPO1	VirusMINT
E6_HPV16	TP53	VirusMINT
E6_HPV16	TP73	VirusMINT
E6_HPV16	TSC2	VirusMINT
E6_HPV16	UBE3A	VirusMINT
E6_HPV16	XRCC1	VirusMINT
E7_HPV16	BRCA1	VirusMINT
E7_HPV16	CDKN1A	VirusMINT
E7_HPV16	CHD4	VirusMINT
E7_HPV16	CREBBP	VirusMINT
E7_HPV16	DNAJA3	VirusMINT
E7_HPV16	EP300	VirusMINT
E7_HPV16	FOS	VirusMINT
E7_HPV16	FOXM1	VirusMINT
E7_HPV16	HDAC1	VirusMINT
E7_HPV16	HDAC2	VirusMINT
E7_HPV16	IRF1	VirusMINT
E7_HPV16	JUN	VirusMINT
E7_HPV16	JUNB	VirusMINT
E7_HPV16	JUND	VirusMINT
E7_HPV16	KAT2B	VirusMINT
E7_HPV16	MYC	VirusMINT
E7_HPV16	NOLC1	VirusMINT
E7_HPV16	PKM2	VirusMINT
E7_HPV16	POU5F1	VirusMINT
E7_HPV16	PPP2CA	VirusMINT
E7_HPV16	PPP2R1A	VirusMINT
E7_HPV16	PSMC1	VirusMINT
E7_HPV16	RAN	VirusMINT
E7_HPV16	RPS25	VirusMINT
E7_HPV16	SIVA1	VirusMINT
E7_HPV16	SNW1	VirusMINT
E7_HPV16	TBP	VirusMINT
L1_HPV16	IPO5	VirusMINT
L1_HPV16	KPNA2	VirusMINT
L1_HPV16	KPNB1	VirusMINT
L1_HPV16	TNPO1	VirusMINT

L2_HPV16	GADD45GIP1	VirusMINT
L2_HPV16	IPO5	VirusMINT
L2_HPV16	KPNA2	VirusMINT
L2_HPV16	KPNB1	VirusMINT
L2_HPV16	MAN2B1	VirusMINT
L2_HPV16	PATZ1	VirusMINT
L2_HPV16	TINAGL1	VirusMINT
L2_HPV16	TNPO1	VirusMINT
E7_HPV16	CCNA2	VirusMINT/Huh et al.
E7_HPV16	CCNE2	VirusMINT/Huh et al.
E7_HPV16	CDC2	VirusMINT/Huh et al.
E7_HPV16	CDK2	VirusMINT/Huh et al.
E7_HPV16	RBL1	VirusMINT/Huh et al.
E7_HPV16	TFDP1	VirusMINT/Huh et al.
E7_HPV16	TFDP2	VirusMINT/Huh et al.
E7_HPV16	E2F1	VirusMINT|Huh et al.
E7_HPV16	E2F2	VirusMINT|Huh et al.
E7_HPV16	E2F3	VirusMINT|Huh et al.
E7_HPV16	E2F4	VirusMINT|Huh et al.
E7_HPV16	RB1	VirusMINT|Huh et al.
E7_HPV16	RBL2	VirusMINT|Huh et al.

Supplementary Table S10. List of genes that show significant expression change in **A**, Burkitt's lymphoma, **B,** B cell lymphoma, **C,** cervical cancer, and **D,** head and neck squamous cell carcinoma.

A

**Gene symbol**	**Ranking**
HES1	-4.26%
CD1D	-2.50%
TRIM22	-2.24%
ITGB2	-1.25%
PTK2	-0.92%
BIRC3	-0.33%
IL6	-0.03%
HSPB1	0.12%
GADD45A	0.22%
HBB	0.24%

FOS	0.52%
VEGFA	0.93%
ACTA2	1.07%
PCNA	1.75%
PTTG1	1.96%
YWHAE	2.15%
SERPINA1	2.47%
MYC	2.48%
CDC2	2.76%
GMPS	3.33%
DHFR	3.47%
ISG15	4.25%
DDB2	4.27%
CHEK1	4.68%
DUSP1	4.96%

B

Gene symbol	Ranking
CASP1	-2.23%
TRIM22	-1.78%
ITGB2	-1.06%
CD1D	-0.24%
IL6	-0.23%
FOS	0.02%
DUSP1	0.08%
CAV1	0.13%
ADA	0.60%
GADD45A	0.81%
ACTA2	1.54%
ATF3	1.58%
TNFRSF10B	2.09%
PCNA	2.27%
GMPS	2.33%
HSPB1	2.34%
TK1	3.13%
CXCL10	3.57%
TP63	3.70%
DDB2	4.64%
PLTP	4.68%

C

Gene symbol	Ranking
SLPI	-4.39%
ISG15	-3.96%
MMP2	-3.42%
DKK1	-1.63%
IVL	-1.24%
IFITM1	-1.20%
KRT16	-0.54%
BCL2A1	0.33%
MMP1	0.50%
PSMB9	0.98%
ATF3	1.05%
ADA	1.40%
CHEK1	1.68%
ICAM1	1.80%
WEE1	1.84%
ODC1	2.41%
IL8	2.45%
TLR3	2.95%
CDK4	3.18%
NDRG1	3.29%
DDB2	3.45%
TGM1	3.96%
FOSL1	4.05%
DUSP1	4.50%
DHFR	4.61%
CYC1	4.87%

D

Gene symbol	Ranking
FOSL1	-4.85%
ITGAX	-4.84%
IL6	-4.20%
FN1	-4.13%
TNFAIP6	-2.07%
IL1B	-1.08%
BCL2A1	-1.02%
DKK1	-0.83%

ODC1	-0.81%
IL8	-0.63%
MMP1	-0.29%
SELE	-0.19%
TLR3	0.38%
DDB2	1.28%
DHFR	1.38%
CDC25A	1.73%
PCNA	1.75%
PTTG1	2.10%
CDC2	2.23%
WEE1	2.36%
PADI3	2.78%
PSMB9	2.87%
MSH2	3.47%
S100A9	4.39%

Supplementary Table S11. Raw data for calculating relative risks of candidate diseases in each viral disease network (PT configuration) among female HPV patients.

ICD-9 code of disease	ICD-9 description	Patients with disease among female HPV patients	Patients with disease among female patients	RR	LB	UB	RR (%99 CI)
1905	Malignant neoplasm of eye, Retina	1	35	15.74	1.20	206.86	15.74
2814	Protein-deficiency anemia	4	273	8.07	2.23	29.26	8.07
1588	Malignant neoplasm of retroperitoneum and peritoneum	15	1329	6.22	3.20	12.09	6.22
183	Malignant neoplasm of ovary and other uterine adnexa	275	30275	5.00	4.28	5.84	5.00
171	Malignant neoplasm of connective and other soft tissue	32	4661	3.78	2.40	5.96	3.78
284	Aplastic anemia (Fanconi anemia)	330	49062	3.70	3.22	4.27	3.70
216	Benign neoplasm of skin	32	5480	3.22	2.04	5.07	3.22
7011	Keratoderma	22	3934	3.08	1.78	5.33	3.08
2880	Diseases of white blood cells, Neutropenia	302	60248	2.76	2.38	3.20	2.76
188	Malignant neoplasm of bladder	137	27706	2.72	2.19	3.39	2.72

170	Malignant neoplasm of bone and articular cartilage	13	2808	2.55	1.25	5.21	2.55
5698	Other disorders of intestine	232	57630	2.22	1.87	2.63	2.22
173	Other malignant neoplasm of skin	81	20316	2.20	1.65	2.92	2.20
7062	Diseases of sebaceous glands, Sebaceous cyst	22	5884	2.06	1.19	3.57	2.06
2114	Benign neoplasm of other parts of digestive system, Rectum and anal canal	24	7684	1.72	1.02	2.91	1.72
709	Other disorders of skin and subcutaneous tissue	40	12947	1.70	1.13	2.56	1.70
2762	Disorders of fluid, electrolyte, and acid-base balance, Acidosis	245	82798	1.63	1.38	1.92	1.63
285	Other and unspecified anemias	3551	1243992	1.57	1.51	1.64	1.57
2738	Disorders of plasma protein metabolism	95	33847	1.55	1.19	2.01	1.55
286	Coagulation defects	151	59988	1.39	1.12	1.71	1.39
278	Overweight, obesity and other hyperalimentation	502	231282	1.20	1.07	1.34	1.20
3343	Spinocerebellar disease, Other cerebellar ataxia	1	2575	0.21	0.02	2.81	1.00
193	Malignant neoplasm of thyroid gland	5	6042	0.46	0.14	1.44	1.00
3334	Other extrapyramidal disease and abnormal movement disorders, Huntington's chorea	2	818	1.35	0.22	8.32	1.00
2592	Other endocrine disorders, Carcinoid syndrome	2	504	2.19	0.35	13.51	1.00
1578	Malignant neoplasm of pancreas	2	2689	0.41	0.07	2.53	1.00
3627	Hereditary retinal dystrophies	2	1008	1.09	0.18	6.76	1.00
3352	Anterior horn cell disease, Motor neuron disease	7	5076	0.76	0.29	2.01	1.00
36274	Other retinal disorders, Pigmentary retinal dystrophy	2	941	1.17	0.19	7.24	1.00
756	Other congenital musculoskeletal anomalies	19	14299	0.73	0.41	1.32	1.00
43491		33	26565	0.68	0.44	1.07	1.00
515	Postinflammatory pulmonary fibrosis	182	106803	0.94	0.78	1.14	1.00
2518	Other disorders of pancreatic internal secretion	15	7743	1.07	0.55	2.08	1.00
147	Malignant neoplasm of nasopharynx	2	672	1.64	0.27	10.13	1.00

2115	Benign neoplasm of liver and biliary passages	3	2293	0.72	0.16	3.19	1.00
36216	Other retinal disorders, Retinal neovascularization	1	487	1.13	0.09	14.87	1.00
2725	Lipoprotein deficiencies	2	1906	0.58	0.09	3.57	1.00
5778	Diseases of pancreas	9	5912	0.84	0.36	1.98	1.00
176	Kaposi's sarcoma	1	358	1.54	0.12	20.22	1.00
295	Schizophrenic disorders	115	54759	1.16	0.91	1.47	1.00
2555	Disorders of adrenal glands	3	756	2.19	0.49	9.67	1.00
281	Other deficiency anemias	226	110959	1.12	0.95	1.33	1.00
7425	Other specified anomalies of spinal cord	1	966	0.57	0.04	7.50	1.00
555	Regional enteritis	36	16592	1.20	0.78	1.84	1.00
720	Ankylosing spondylitis	7	6453	0.60	0.23	1.58	1.00
6951	Erythema multiforme	6	3019	1.09	0.38	3.13	1.00
359	Muscular dystrophies	7	8105	0.48	0.18	1.26	1.00
5188	Other diseases of lung	523	308303	0.93	0.83	1.05	1.00
172	Malignant melanoma of skin	14	5427	1.42	0.71	2.83	1.00
2722	Disorders of lipoid metabolism, Mixed hyperlipidemia	20	10873	1.01	0.57	1.80	1.00
3314	Other cerebral degenerations, Obstructive hydrocephalus	24	15999	0.83	0.49	1.40	1.00
2872	Purpura and other hemorrhagic conditions	1	1082	0.51	0.04	6.69	1.00
151	Malignant neoplasm of stomach	15	12184	0.68	0.35	1.32	1.00
3561	Hereditary and idiopathic peripheral neuropathy, Peroneal muscular atrophy	1	925	0.60	0.05	7.83	1.00
250	Diabetes mellitus	2243	1267230	0.97	0.92	1.03	1.00
2773	Amyloidosis	4	1765	1.25	0.34	4.53	1.00
9952	Certain adverse effects not elsewhere classified	95	64700	0.81	0.62	1.05	1.00
2794	Autoimmune disease	1	1088	0.51	0.04	6.65	1.00
205	Myeloid leukemia	15	6822	1.21	0.62	2.36	1.00
2113	Benign neoplasm of other parts of digestive system, Colon	179	95181	1.04	0.85	1.26	1.00
7819	Symptoms involving nervous and musculoskeletal systems	5	4739	0.58	0.18	1.84	1.00
2753	Disorders of phosphorus metabolism	31	10916	1.56	0.98	2.48	1.00
191	Malignant neoplasm of brain	9	6774	0.73	0.31	1.73	1.00
7090	Other disorders of skin and subcutaneous tissue, Dyschromia	3	1341	1.23	0.28	5.45	1.00
2377	Neurofibromatosis	4	963	2.29	0.63	8.29	1.00

496	Chronic airway obstruction	1714	893108	1.06	0.99	1.12	1.00
162	Malignant neoplasm of trachea, bronchus, and lung	157	83337	1.04	0.84	1.27	1.00
731	Osteitis deformans and osteopathies associated with other disorders	17	15587	0.60	0.32	1.12	1.00
340	Multiple sclerosis	14	11692	0.66	0.33	1.31	1.00
2028	Other malignant neoplasms of lymphoid and histiocytic tissue	46	26534	0.95	0.65	1.40	1.00
345	Epilepsy and recurrent seizures	89	59545	0.82	0.63	1.08	1.00
2393	Neoplasms of unspecified nature, Breast	2	1512	0.73	0.12	4.50	1.00
283	Acquired hemolytic anemias	29	11554	1.38	0.86	2.23	1.00
208	Leukemia of unspecified cell type	11	3809	1.59	0.73	3.46	1.00
7573	Congenital anomalies of the integument	1	453	1.22	0.09	15.98	1.00
389	Hearing loss	69	42907	0.89	0.65	1.21	1.00
7598	Other and unspecified congenital anomalies	1	343	1.61	0.12	21.11	1.00
204	Lymphoid leukemia	30	21405	0.77	0.48	1.24	1.00
260	Kwashiorkor	21	6784	1.71	0.97	2.99	1.00
5718	Chronic liver disease and cirrhosis	22	8948	1.35	0.78	2.35	1.00
2948	Persistent mental disorders due to conditions classified elsewhere	12	10157	0.65	0.31	1.37	1.00
333	Other extrapyramidal disease and abnormal movement disorders	51	39712	0.71	0.49	1.01	1.00
710	Diffuse diseases of connective tissue	53	37027	0.79	0.55	1.12	1.00
7578	Congenital anomalies of the integument	0		1.00			1.00
75731	Congenital anomalies of the integument	0		1.00			1.00
75567	Other congenital anomalies of limbs, Anomalies of foot	0		1.00			1.00
2572	Testicular dysfunction	0		1.00			1.00
2533	Disorders of the pituitary gland and its hypothalamic control, Pituitary dwarfism	0		1.00			1.00
185	Malignant neoplasm of prostate	0		1.00			1.00
99685	Complications peculiar to certain specified procedures, Bone	0		1.00			1.00

	marrow			
9747	Poisoning by water, mineral, and uric acid metabolism drugs	0	1.00	1.00
2534	Disorders of the pituitary gland and its hypothalamic control	0	1.00	1.00
2002	Burkitt's lymphoma	0	1.00	1.00
7467	Other congenital anomalies of heart, Hypoplastic left heart syndrome	0	1.00	1.00
37446	Other disorders of eyelids, Blepharophimosis	0	1.00	1.00
7554	Other congenital anomalies of limbs, Reduction deformities, unspecified limb	0	1.00	1.00
7551	Other congenital anomalies of limbs, Syndactyly	0	1.00	1.00
5204	Disturbances of tooth formation	0	1.00	1.00
27785	Disorders of fatty acid oxidation	0	1.00	1.00
7595	Other and unspecified congenital anomalies, Tuberous sclerosis	0	1.00	1.00
6425	Severe pre-eclampsia	0	1.00	1.00
7571	Congenital anomalies of the integument, Ichthyosis congenita	0	1.00	1.00
75733	Congenital anomalies of the integument, Congenital pigmentary anomalies of skin	0	1.00	1.00
2840	Aplastic anemia and other bone marrow failure syndromes,	0	1.00	1.00
7575	Congenital anomalies of the integument,	0	1.00	1.00
27731	Familial Mediterranean fever	0	1.00	1.00
194	Malignant neoplasm of other endocrine glands	0	1.00	1.00
3783	Strabismus and other disorders of binocular eye movements,	0	1.00	1.00
99527	Certain adverse effects not elsewhere classified, Other drug allergy	0	1.00	1.00

0846	Malaria	0		1.00			1.00
99591	Certain adverse effects not elsewhere classified, Sepsis	0		1.00			1.00
7555	Other congenital anomalies of limbs, Other anomalies of upper limb	0		1.00			1.00
2772	Other disorders of purine and pyrimidine metabolism	0		1.00			1.00
2792	Combined immunity deficiency	0		1.00			1.00
7422	Other congenital anomalies of nervous system,	0		1.00			1.00
2726	Disorders of lipoid metabolism, Lipodystrophy	0		1.00			1.00
2777	Other and unspecified disorders of metabolism, Dysmetabolic syndrome X	0		1.00			1.00
042	Human immunodeficiency virus [HIV]	0		1.00			1.00
2718	Disorders of carbohydrate transport and metabolism	0		1.00			1.00
3685	Visual disturbances, Color vision deficiencies	0		1.00			1.00
36854	Visual disturbances, Achromatopsia	0		1.00			1.00
440	Atherosclerosis	447	283986	0.87	0.77	0.98	0.87
714	Rheumatoid arthritis and other inflammatory polyarthropathies	218	144625	0.83	0.70	0.99	0.83
733	Other disorders of bone and cartilage	735	500591	0.81	0.74	0.89	0.81
493	Asthma	443	308106	0.79	0.70	0.90	0.79
244	Acquired hypothyroidism	795	577982	0.76	0.69	0.83	0.76
153	Malignant neoplasm of colon	164	119862	0.75	0.62	0.92	0.75
4261	Conduction disorders, Atrioventricular block, other and unspecified	204	150876	0.74	0.62	0.89	0.74
4254	Cardiomyopathy	202	151629	0.73	0.61	0.88	0.73
577	Diseases of pancreas	148	117189	0.70	0.56	0.86	0.70
331	Other cerebral degenerations	266	217698	0.67	0.57	0.79	0.67
272	Disorders of lipoid metabolism	489	447192	0.60	0.54	0.68	0.60
271	Disorders of carbohydrate transport and metabolism	23	22084	0.57	0.34	0.98	0.57

332	Parkinson's disease	122	119034	0.56	0.45	0.71	0.56
410	Acute myocardial infarction	450	452653	0.55	0.48	0.62	0.55
346	Migraine	15	21183	0.39	0.20	0.76	0.39

Supplementary Table S12. Raw data for calculating relative risks of candidate diseases in each viral disease network (PT configuration) among male HPV patients.

ICD-9 code of disease	ICD-9 description	Patients with disease among male HPV patients	Patients with disease among male patients	RR	LB	UB	RR (%99 CI)
216	Benign neoplasm of skin	18	3673	21.47	1.21	381.63	21.47
7062	Diseases of sebaceous glands, Sebaceous cyst	20	5905	14.84	1.50	146.83	14.84
173	Other malignant neoplasm of skin	41	22670	7.92	2.38	26.35	7.92
188	Malignant neoplasm of bladder	39	82204	2.08	1.01	4.29	2.08
5188	Other diseases of lung, Other diseases of lung	91	253255	1.57	1.02	2.43	1.57
496	Chronic airway obstruction, not elsewhere classified	304	1025277	1.30	1.04	1.62	1.30
3343	Spinocerebellar disease, Other cerebellar ataxia	2	2472	1.00	0.07	172.11	1.00
577	Diseases of pancreas	20	74922	1.00	0.50	2.73	1.00
3352	Anterior horn cell disease, Motor neuron disease	2	5345	1.00	0.09	31.60	1.00
5778	Diseases of pancreas, Other specified diseases of pancreas	2	3804	1.00	0.08	63.10	1.00
2518	Other disorders of pancreatic internal secretion, Other specified disorders of pancreatic internal secretion	4	5615	1.00	0.23	42.63	1.00
332	Parkinson's disease	35	109084	1.00	0.72	2.76	1.00
3561	Hereditary and idiopathic peripheral neuropathy, Peroneal muscular atrophy	1	1076	1.00	0.01	1345.84	1.00
271	Disorders of carbohydrate transport and metabolism	4	14184	1.00	0.18	8.47	1.00
170	Malignant neoplasm of bone and articular cartilage	2	2466	1.00	0.07	173.16	1.00

193	Malignant neoplasm of thyroid gland	3	2547	1.00	0.13	206.84	1.00
710	Diffuse diseases of connective tissue	1	9114	1.00	0.02	11.04	1.00
272	Disorders of lipoid metabolism	53	258825	1.00	0.55	1.46	1.00
286	Coagulation defects	24	54151	1.00	0.79	4.78	1.00
733	Other disorders of bone and cartilage	30	96640	1.00	0.66	2.80	1.00
331	Other cerebral degenerations	33	113963	1.00	0.65	2.49	1.00
5718	Chronic liver disease and cirrhosis, Other chronic nonalcoholic liver disease	2	4333	1.00	0.09	47.96	1.00
333	Other extrapyramidal disease and abnormal movement disorders	11	21297	1.00	0.56	9.20	1.00
284	Aplastic anemia and other bone marrow failure syndromes	12	38203	1.00	0.44	4.33	1.00
515	Postinflammatory pulmonary fibrosis	25	87653	1.00	0.58	2.71	1.00
440	Atherosclerosis	67	247886	1.00	0.74	1.89	1.00
756	Other congenital musculoskeletal anomalies	1	5878	1.00	0.02	22.40	1.00
43491		7	18064	1.00	0.34	8.40	1.00
7011	Other hypertrophic and atrophic conditions of skin, Keratoderma, acquired	9	2832	1.00	0.50	383.83	1.00
281	Other deficiency anemias	19	60729	1.00	0.55	3.40	1.00
7425	Other congenital anomalies of nervous system, Other specified anomalies of spinal cord	1	950	1.00	0.01	2059.75	1.00
555	Regional enteritis	6	9031	1.00	0.36	23.29	1.00
2753	Disorders of mineral metabolism, Disorders of phosphorus metabolism	1	8625	1.00	0.02	12.01	1.00
9952	Certain adverse effects not elsewhere classified	11	30981	1.00	0.45	5.38	1.00
205	Myeloid leukemia	1	7233	1.00	0.02	15.84	1.00
244	Acquired hypothyroidism	38	125031	1.00	0.70	2.52	1.00
720	Ankylosing spondylitis and other inflammatory spondylopathies	3	6124	1.00	0.15	30.01	1.00

359	Muscular dystrophies and other myopathies	2	5763	1.00	0.08	27.40	1.00
295	Schizophrenic disorders	9	20291	1.00	0.45	8.48	1.00
2738	Disorders of plasma protein metabolism, Other disorders of plasma protein metabolism	6	19398	1.00	0.27	6.80	1.00
172	Malignant melanoma of skin	5	5610	1.00	0.30	50.06	1.00
2773	Other and unspecified disorders of metabolism, Amyloidosis	1	1662	1.00	0.02	358.12	1.00
3314	Other cerebral degenerations, Obstructive hydrocephalus	2	13314	1.00	0.06	6.87	1.00
171	Malignant neoplasm of connective and other soft tissue	2	4146	1.00	0.08	52.56	1.00
278	Overweight, obesity and other hyperalimentation	17	86894	1.00	0.37	2.01	1.00
250	Diabetes mellitus	259	910904	1.00	0.98	1.58	1.00
185	Malignant neoplasm of prostate	114	397870	1.00	0.87	1.80	1.00
2113	Benign neoplasm of other parts of digestive system, Colon	23	83746	1.00	0.54	2.67	1.00
7819	Symptoms involving nervous and musculoskeletal systems, Other symptoms involving nervous and musculoskeletal systems	2	3537	1.00	0.08	73.96	1.00
781	Symptoms involving nervous and musculoskeletal systems	7	64101	1.00	0.15	1.56	1.00
151	Malignant neoplasm of stomach	4	15965	1.00	0.17	7.09	1.00
2114	Benign neoplasm of other parts of digestive system, Rectum and anal canal	4	6290	1.00	0.23	34.14	1.00
4254	Cardiomyopathy, Other primary cardiomyopathies	54	160371	1.00	0.85	2.56	1.00
162	Malignant neoplasm of trachea, bronchus, and lung	24	115617	1.00	0.44	1.88	1.00
2028	Other malignant neoplasms of lymphoid and histiocytic tissue, Other lymphomas	7	21125	1.00	0.32	6.67	1.00
285	Other and unspecified anemias	219	800509	1.00	0.93	1.55	1.00
2880	Diseases of white blood cells, Neutropenia	17	38607	1.00	0.66	5.62	1.00

389	Hearing loss	5	33353	1.00	0.15	2.89	1.00
731	Osteitis deformans and osteopathies associated with other disorders classified elsewhere	3	14824	1.00	0.11	6.84	1.00
153	Malignant neoplasm of colon	16	97242	1.00	0.31	1.68	1.00
204	Lymphoid leukemia	9	22077	1.00	0.43	7.49	1.00
042	Human immunodeficiency virus [HIV] disease	3	831	1.00	0.04	7039.68	1.00
340	Multiple sclerosis	4	4630	1.00	0.23	63.32	1.00
345	Epilepsy and recurrent seizures	10	46076	1.00	0.30	2.97	1.00
714	Rheumatoid arthritis and other inflammatory polyarthropathies	11	47669	1.00	0.34	3.04	1.00
2948	Persistent mental disorders due to conditions classified elsewhere	1	6043	1.00	0.02	21.36	1.00
4261	Conduction disorders, Atrioventricular block, other and unspecified	32	159443	1.00	0.47	1.64	1.00
493	Asthma	43	154421	1.00	0.68	2.19	1.00
7573	Congenital anomalies of the integument, Other specified anomalies of skin	1	277	1.00	0.00	611507.37	1.00
208	Leukemia of unspecified cell type	1	3820	1.00	0.03	49.96	1.00
5698	Other disorders of intestine, Other specified disorders of intestine	12	34468	1.00	0.47	4.97	1.00
709	Other disorders of skin and subcutaneous tissue	8	8256	1.00	0.53	34.17	1.00
191	Malignant neoplasm of brain	1	5929	1.00	0.02	22.07	1.00
2762	Disorders of fluid, electrolyte, and acid-base balance, Acidosis	20	59786	1.00	0.59	3.62	1.00
346	Migraine	2	6041	1.00	0.08	25.10	1.00
283	Acquired hemolytic anemias	6	7041	1.00	0.38	36.78	1.00
37446	Other disorders of eyelids, Blepharophimosis	0		1.00			1.00
5204	Disorders of tooth development and eruption, Disturbances of tooth formation	0		1.00			1.00

7578	Congenital anomalies of the integument, Other specified anomalies of the integument	0	1.00	1.00
2002	Lymphosarcoma and reticulosarcoma and other specified malignant tumors of lymphatic tissue, Burkitt's tumor or lymphoma	0	1.00	1.00
2872	Purpura and other hemorrhagic conditions, Other nonthrombocytopenic purpuras	0	1.00	1.00
2533	Disorders of the pituitary gland and its hypothalamic control, Pituitary dwarfism	0	1.00	1.00
2377	Neoplasm of uncertain behavior of endocrine glands and nervous system, Neurofibromatosis	0	1.00	1.00
7467	Other congenital anomalies of heart, Hypoplastic left heart syndrome	0	1.00	1.00
7551	Other congenital anomalies of limbs, Syndactyly	0	1.00	1.00
7598	Other and unspecified congenital anomalies, Other specified anomalies	0	1.00	1.00
2393	Neoplasms of unspecified nature, Breast	0	1.00	1.00
2722	Disorders of lipoid metabolism, Mixed hyperlipidemia	0	1.00	1.00
1588	Malignant neoplasm of retroperitoneum and peritoneum, Specified parts of peritoneum	0	1.00	1.00
7595	Other and unspecified congenital anomalies, Tuberous sclerosis	0	1.00	1.00
99527	Certain adverse effects not elsewhere classified, Other drug allergy	0	1.00	1.00
1905	Malignant neoplasm of eye, Retina	0	1.00	1.00

6951	Erythematous conditions, Erythema multiforme	0		1.00		1.00
75731	Congenital anomalies of the integument, Congenital ectodermal dysplasia	0		1.00		1.00
75567	Other congenital anomalies of limbs, Anomalies of foot, NEC	0		1.00		1.00
7554	Other congenital anomalies of limbs, Reduction deformities, unspecified limb	0		1.00		1.00
2794	Disorders involving the immune mechanism, Autoimmune disease, not elsewhere classified	0		1.00		1.00
2555	Disorders of adrenal glands, Other adrenal hypofunction	0		1.00		1.00
183	Malignant neoplasm of ovary and other uterine adnexa	0		1.00		1.00
7555	Other congenital anomalies of limbs, Other anomalies of upper limb, including shoulder girdle	0		1.00		1.00
176	Kaposi's sarcoma	0		1.00		1.00
2840	Aplastic anemia and other bone marrow failure syndromes, Constitutional aplastic anemia	0		1.00		1.00
7571	Congenital anomalies of the integument, Ichthyosis congenita	0		1.00		1.00
36274	Other retinal disorders, Pigmentary retinal dystrophy	0		1.00		1.00
7575	Congenital anomalies of the integument, Specified anomalies of nails	0		1.00		1.00
3627	Other retinal disorders, Hereditary retinal dystrophies	0		1.00		1.00
2792	Disorders involving the immune mechanism, Combined immunity deficiency	0		1.00		1.00
147	Malignant neoplasm of nasopharynx	0		1.00		1.00

36216	Other retinal disorders, Retinal neovascularization NOS	0	1.00	1.00
2725	Disorders of lipoid metabolism, Lipoprotein deficiencies	0	1.00	1.00
99685	Complications peculiar to certain specified procedures, Bone marrow	0	1.00	1.00
6425	Hypertension complicating pregnancy, childbirth, and the puerperium, Severe pre-eclampsia	0	1.00	1.00
2814	Other deficiency anemias, Protein-deficiency anemia	0	1.00	1.00
7422	Other congenital anomalies of nervous system, Reduction deformities of brain	0	1.00	1.00
27785	Other and unspecified disorders of metabolism, Disorders of fatty acid oxidation	0	1.00	1.00
3783	Strabismus and other disorders of binocular eye movements, Other and unspecified heterotropia	0	1.00	1.00
0846	Malaria, Malaria, unspecified	0	1.00	1.00
260	Kwashiorkor	0	1.00	1.00
2534	Disorders of the pituitary gland and its hypothalamic control, Other anterior pituitary disorders	0	1.00	1.00
36854	Visual disturbances, Achromatopsia	0	1.00	1.00
3685	Visual disturbances, Color vision deficiencies	0	1.00	1.00
9747	Poisoning by water, mineral, and uric acid metabolism drugs, Uric acid metabolism drugs	0	1.00	1.00
2718	Disorders of carbohydrate transport and metabolism, Other specified disorders of	0	1.00	1.00

	carbohydrate transport and metabolism						
2726	Disorders of lipoid metabolism, Lipodystrophy	0		1.00			1.00
2772	Other and unspecified disorders of metabolism, Other disorders of purine and pyrimidine metabolism	0		1.00			1.00
7090	Other disorders of skin and subcutaneous tissue, Dyschromia	0		1.00			1.00
2777	Other and unspecified disorders of metabolism, Dysmetabolic syndrome X	0		1.00			1.00
99591	Certain adverse effects not elsewhere classified, Sepsis	0		1.00			1.00
1578	Malignant neoplasm of pancreas, Other specified sites of pancreas	0		1.00			1.00
194	Malignant neoplasm of other endocrine glands and related structures	0		1.00			1.00
2115	Benign neoplasm of other parts of digestive system, Liver and biliary passages	0		1.00			1.00
2572	Testicular dysfunction, Other testicular hypofunction	0		1.00			1.00
3334	Other extrapyramidal disease and abnormal movement disorders, Huntington's chorea	0		1.00			1.00
27731	Other and unspecified disorders of metabolism, Familial Mediterranean fever	0		1.00			1.00
2592	Other endocrine disorders, Carcinoid syndrome	0		1.00			1.00
75733	Congenital anomalies of the integument, Congenital pigmentary anomalies of skin	0		1.00			1.00
410	Acute myocardial infarction	69	490175	0.62	0.42	0.91	0.62

Supplementary Table S13. Raw data for calculating relative risks of candidate diseases in each viral disease network (PT configuration) among EBV patients.

ICD-9 code of disease	ICD-9 description	Patients with disease among EBV patients	Patients with disease among ALL patients	RR	LB	UB	RR (%99 CI)
147	Malignant neoplasm of nasopharynx	1463	1463	522.10	488.10	558.48	522.10
2002	Burkitt's tumor or lymphoma	217	217	522.10	438.34	621.87	522.10
2078	Other specified leukemia, Other specified leukemia	52	202	134.40	94.03	192.11	134.40
2028	Other malignant neoplasms of lymphoid and histiocytic tissue	10883	47659	119.22	116.32	122.20	119.22
207	Other specified leukemia	54	580	48.61	34.24	69.02	48.61
27900	Hypogammaglobulinemia	121	2278	27.73	21.94	35.05	27.73
204	Lymphoid leukemia	1803	43482	21.65	20.38	23.00	21.65
186	Malignant neoplasm of testis	18	440	21.36	11.64	39.20	21.36
2840	Aplastic anemia and other bone marrow failure syndromes, Constitutional aplastic anemia	11	323	17.78	8.18	38.66	17.78
208	Leukemia of unspecified cell	213	7629	14.58	12.22	17.39	14.58
170	Malignant neoplasm of bone and articular cartilage	84	5274	8.32	6.28	11.01	8.32
2875	Purpura and other hemorrhagic conditions, Thrombocytopenia	1800	113055	8.31	7.82	8.83	8.31
287	Purpura and other hemorrhagic conditions	2561	161730	8.27	7.86	8.70	8.27
2792	Combined immunity deficiency	2	143	7.30	1.18	45.14	7.30
171	Malignant neoplasm of connective and other soft tissue	110	8807	6.52	5.10	8.34	6.52
176	Kaposi's sarcoma	9	835	5.63	2.38	13.28	5.63
7571	Congenital anomalies of the integument, Ichthyosis congenita	5	517	5.05	1.60	15.98	5.05
2826	Hereditary hemolytic anemias, Sickle-cell disease	8	910	4.59	1.85	11.41	4.59

191	Malignant neoplasm of brain	106	12703	4.36	3.39	5.60	4.36
260	Kwashiorkor	85	10905	4.07	3.08	5.38	4.07
2381	Neoplasm of uncertain behavior of other and unspecified sites and tissues, Connective and other soft tissue	6	826	3.79	1.32	10.86	3.79
2554	Disorders of adrenal glands, Corticoadrenal insufficiency	128	19015	3.51	2.80	4.41	3.51
151	Malignant neoplasm of stomach	186	28149	3.45	2.86	4.17	3.45
5234	Gingival and periodontal diseases, Chronic periodontitis	14	2186	3.34	1.68	6.66	3.34
6951	Erythematous conditions, Erythema multiforme	26	4475	3.03	1.83	5.03	3.03
203	Multiple myeloma and immunoproliferative neoplasms	162	28605	2.96	2.42	3.62	2.96
2871	Purpura and other hemorrhagic conditions, Qualitative platelet defects	13	2317	2.93	1.43	5.98	2.93
173	Other malignant neoplasm of skin	236	42986	2.87	2.42	3.39	2.87
161	Malignant neoplasm of larynx	75	13760	2.85	2.11	3.83	2.85
193	Malignant neoplasm of thyroid gland	46	8589	2.80	1.91	4.09	2.80
194	Malignant neoplasm of other endocrine glands and related structures	9	1681	2.80	1.18	6.60	2.80
355	Mononeuritis of lower limb	172	34123	2.63	2.16	3.20	2.63
3561	Hereditary and idiopathic peripheral neuropathy, Peroneal muscular atrophy	10	2001	2.61	1.16	5.89	2.61
2773	Other and unspecified disorders of metabolism, Amyloidosis	17	3427	2.59	1.39	4.84	2.59
5698	Other disorders of intestine, Other specified disorders of intestine	448	92098	2.54	2.25	2.87	2.54
7090	Other disorders of skin and subcutaneous tissue	11	2272	2.53	1.16	5.50	2.53
172	Malignant melanoma of skin	47	11037	2.22	1.53	3.24	2.22

285	Other and unspecified anemias	8353	2044501	2.13	2.07	2.19	2.13
286	Coagulation defects	437	114139	2.00	1.77	2.26	2.00
216	Benign neoplasm of skin	34	9153	1.94	1.25	3.02	1.94
2824	Hereditary hemolytic anemias, Thalassemias	35	9689	1.89	1.22	2.92	1.89
515	Postinflammatory pulmonary fibrosis	644	194456	1.73	1.56	1.91	1.73
370	Keratitis	34	10477	1.69	1.09	2.64	1.69
733	Other disorders of bone and cartilage	1833	597231	1.60	1.51	1.70	1.60
4254	Cardiomyopathy, Other primary cardiomyopathies	759	312000	1.27	1.16	1.39	1.27
244	Acquired hypothyroidism	1695	703013	1.26	1.18	1.34	1.26
2113	Benign neoplasm of other parts of digestive system, Colon	403	178927	1.18	1.03	1.34	1.18
162	Malignant neoplasm of trachea, bronchus, and lung	437	198954	1.15	1.01	1.30	1.15
3334	Other extrapyramidal disease and abnormal movement disorders, Huntington's chorea	1	1331	0.39	0.03	5.16	1.00
28311	Acquired hemolytic anemias, Hemolytic-uremic syndrome	2	176	5.93	0.96	36.67	1.00
3343	Spinocerebellar disease, Other cerebellar ataxia	12	5047	1.24	0.59	2.61	1.00
268	Vitamin D deficiency	5	1904	1.37	0.43	4.34	1.00
720	Ankylosing spondylitis and other inflammatory spondylopathies	25	12577	1.04	0.62	1.74	1.00
2897	Other diseases of blood and blood-forming organs, Methemoglobinemia	1	160	3.26	0.25	42.89	1.00
3774	Disorders of optic nerve and visual pathways, Other disorders of optic nerve	9	2678	1.75	0.74	4.14	1.00
743	Congenital anomalies of eye	2	783	1.33	0.22	8.24	1.00
366	Cataract	185	100132	0.96	0.80	1.17	1.00
2827	Hereditary hemolytic anemias, Other hemoglobinopathies	1	609	0.86	0.07	11.27	1.00
744	Congenital anomalies of ear, face, and neck	3	687	2.28	0.52	10.09	1.00

2114	Benign neoplasm of other parts of digestive system, Rectum and anal canal	39	13974	1.46	0.96	2.20	1.00
2872	Purpura and other hemorrhagic conditions, Other nonthrombocytopenic purpuras	7	1699	2.15	0.81	5.70	1.00
7596	Other and unspecified congenital anomalies, Other hamartoses, NEC	5	1498	1.74	0.55	5.51	1.00
1578	Malignant neoplasm of pancreas, Other specified sites of pancreas	11	4810	1.19	0.55	2.60	1.00
183	Malignant neoplasm of ovary and other uterine adnexa	45	30302	0.78	0.53	1.14	1.00
2377	Neoplasm of uncertain behavior of endocrine glands and nervous system, Neurofibromatosis	7	1757	2.08	0.79	5.51	1.00
153	Malignant neoplasm of colon	426	217104	1.02	0.90	1.16	1.00
2115	Benign neoplasm of other parts of digestive system, Liver and biliary passages	12	3487	1.80	0.85	3.78	1.00
4928	Emphysema, Other emphysema	723	356906	1.06	0.96	1.16	1.00
7062	Diseases of sebaceous glands, Sebaceous cyst	29	11789	1.28	0.80	2.07	1.00
7819	Symptoms involving nervous and musculoskeletal systems, Other symptoms involving nervous and musculoskeletal systems	14	8276	0.88	0.44	1.76	1.00
555	Regional enteritis	56	25623	1.14	0.81	1.61	1.00
3625	Other retinal disorders, Degeneration of macula and posterior pole	88	37575	1.22	0.93	1.61	1.00
2864	Coagulation defects, von Willebrand's disease	7	1463	2.50	0.94	6.61	1.00
2393	Neoplasms of unspecified nature, Breast	3	1540	1.02	0.23	4.50	1.00
365	Glaucoma	430	233225	0.96	0.85	1.09	1.00
3627	Other retinal disorders, Hereditary retinal dystrophies	4	1691	1.24	0.34	4.48	1.00

75567	Other congenital anomalies of limbs, Anomalies of foot, NEC	1	157	3.33	0.25	43.71	1.00
7555	Other congenital anomalies of limbs, Other anomalies of upper limb, including shoulder girdle	1	720	0.73	0.06	9.53	1.00
2725	Disorders of lipoid metabolism, Lipoprotein deficiencies	2	3133	0.33	0.05	2.06	1.00
2881	Diseases of white blood cells, Functional disorders of polymorphonuclear neutrophils	4	809	2.58	0.71	9.36	1.00
587	Renal sclerosis, unspecified	9	3552	1.32	0.56	3.12	1.00
7598	Other and unspecified congenital anomalies, Other specified anomalies	1	651	0.80	0.06	10.54	1.00
2721	Disorders of lipoid metabolism, Pure hyperglyceridemia	59	34104	0.90	0.65	1.26	1.00
3351	Anterior horn cell disease, Spinal muscular atrophy	2	252	4.14	0.67	25.61	1.00
2592	Other endocrine disorders, Carcinoid syndrome	1	862	0.61	0.05	7.96	1.00
3715	Corneal opacity and other disorders of cornea, Hereditary corneal dystrophies	8	4268	0.98	0.39	2.43	1.00
714	Rheumatoid arthritis and other inflammatory polyarthropathies	415	192294	1.13	0.99	1.28	1.00
27785	Other and unspecified disorders of metabolism, Disorders of fatty acid oxidation	0		1.00			1.00
2772	Other and unspecified disorders of metabolism, Other disorders of purine and pyrimidine metabolism	0		1.00			1.00
74344	Congenital anomalies of eye, Specified anomalies of anterior chamber, chamber angle, and related structures	0		1.00			1.00

7431	Congenital anomalies of eye, Microphthalmos	0		1.00			1.00
25513	Disorders of adrenal glands, Bartter's syndrome	0		1.00			1.00
2886	Diseases of white blood cells, Elevated white blood cell count	0		1.00			1.00
7568	Other congenital musculoskeletal anomalies, Other specified anomalies of muscle, tendon, fascia, and connective tissue	0		1.00			1.00
2702	Disorders of amino-acid transport and metabolism, Other disturbances of aromatic amino-acid metabolism	0		1.00			1.00
75564	Other congenital anomalies of limbs, Congenital deformity of knee (joint)	0		1.00			1.00
7554	Other congenital anomalies of limbs, Reduction deformities, unspecified limb	0		1.00			1.00
27786	Other and unspecified disorders of metabolism, Peroxisomal disorders	0		1.00			1.00
7578	Congenital anomalies of the integument, Other specified anomalies of the integument	0		1.00			1.00
2814	Other deficiency anemias, Protein-deficiency anemia	0		1.00			1.00
75731	Congenital anomalies of the integument, Congenital ectodermal dysplasia	0		1.00			1.00
37446	Other disorders of eyelids, Blepharophimosis	0		1.00			1.00
75683	Other congenital musculoskeletal anomalies, Ehlers-Danlos syndrome	0		1.00			1.00
75733	Congenital anomalies of the integument, Congenital pigmentary anomalies of skin	0		1.00			1.00

7434	Congenital anomalies of eye, Coloboma and other anomalies of anterior segment	0		1.00			1.00
1905	Malignant neoplasm of eye, Retina	0		1.00			1.00
99527	Certain adverse effects not elsewhere classified, Other drug allergy	0		1.00			1.00
74345	Congenital anomalies of eye, Aniridia	0		1.00			1.00
74357	Congenital anomalies of eye, Specified anomalies of optic disc	0		1.00			1.00
2884	Diseases of white blood cells, Hemophagocytic syndromes	0		1.00			1.00
75833	Chromosomal anomalies, Other microdeletions	0		1.00			1.00
250	Diabetes mellitus	3943	2178134	0.95	0.91	0.98	0.95
185	Malignant neoplasm of prostate	679	397991	0.89	0.81	0.98	0.89
401	Essential hypertension	7673	4649322	0.86	0.84	0.89	0.86
188	Malignant neoplasm of bladder	170	109910	0.81	0.66	0.98	0.81
332	Parkinson's disease	303	228118	0.69	0.60	0.80	0.69
331	Other cerebral degenerations	311	331661	0.49	0.42	0.57	0.49

Supplementary Table S14. Genes in the network vicinity of the viral targets that differentially expressed in gene expression profiles of normal human diploid fibroblast (IMR90 data from Rozenblatt-Rosen, et al, provided herein for clarity) and primary human keratinocyte (Ker) populations with stable expression of HPV16 E6 or E7 oncoproteins.

Differentially expressed gene	Cell type	ICD-9 code	Disease Name
APP	E7 (Ker)	255.5	Disorders of adrenal glands
APP	E7 (Ker)	277.39	Other and unspecified disorders of metabolism
APP	E7 (Ker)	295.9	Schizophrenic disorders
APP	E7 (Ker)	331	Other cerebral degenerations

**BAX**	E6 (IMR90) E6 (Ker)	**153**	**Malignant neoplasm of colon**
BAX	E6 (IMR90) E6 (Ker)	204	Lymphoid leukemia
BCL2	E7 (IMR90) E7 (Ker)	200	B-cell lymphoma
BRCA1	E6 (IMR90) E7 (IMR90) E7 (Ker)	158.8	Malignant neoplasm of retroperitoneum and peritoneum
**BRCA1**	E6 (IMR90) E7 (IMR90) E7 (Ker)	**183**	**Malignant neoplasm of ovary**
**BRCA1**	E6 (IMR90) E7 (IMR90) E7 (Ker)	**239.3**	**Neoplasms of unspecified nature, Breast**
CASP1	E6 (Ker)	-	
CCL5	E6 (IMR90)	042	Human immunodeficiency virus [HIV] disease
**CD82**	E6 (IMR90)	**185**	**Malignant neoplasm of prostate**
CDC25A	E6 (IMR90) E7 (IMR90) E7 (Ker)	-	
CDK4	E6 (IMR90) E7 (IMR90)	172.9	Malignant melanoma of skin
CDKN1B	E6 (IMR90) E7 (Ker)	-	
CHEK1	E6 (IMR90)	-	
CYC1	E7 (Ker)	-	
DDB2	E6 (IMR90)	757.33	Congenital anomalies of the integument
DUSP1	E6 (IMR90)	-	
E2F1	E7 (Ker)	-	
EDN1	E7 (IMR90)	286.9	Coagulation defects
EIF4E	E7 (Ker)	-	
ERBB2	E7 (Ker)	151.9	Malignant neoplasm of stomach
**ERBB2**	E7 (Ker)	**183**	**Malignant neoplasm of ovary**
ERBB2	E7 (Ker)	191.9	Malignant neoplasm of brain
ERBB2	E7 (Ker)	272.2	Disorders of lipoid metabolism
FANCC	E6 (IMR90)	284.09	Aplastic anemia (Fanconi Anemia)
**FN1**	E7 (Ker)	**183**	**Malignant neoplasm of ovary**
FN1	E7 (Ker)	208.9	Leukemia of unspecified cell type
FN1	E7 (Ker)	569.89	Other disorders of intestine, Other
FOSL1	E7 (Ker)	-	
GADD45A	E6 (IMR90)	-	
GJA1	E7 (Ker)	389.9	Hearing loss
GJA1	E7 (Ker)	426.1	Conduction disorders
GJA1	E7 (Ker)	746.7	Other congenital anomalies of heart
GJA1	E7 (Ker)	755.1	Other congenital anomalies of limbs
GJA1	E7 (Ker)	759.89	Other and unspecified congenital anomalies
HADHA	E7 (Ker)	277.85	Other and unspecified disorders of metabolism
HADHA	E7 (Ker)	571.8	Chronic liver disease and cirrhosis
HADHA	E7 (Ker)	642.5	Hypertension complicating pregnancy
IFITM1	E7 (Ker)	-	
IGFBP3	E6 (IMR90)	-	

ISG15	E7 (Ker)	-	
IVL	E7 (Ker)	-	
KRT16	E7 (Ker)	757.39	Congenital anomalies of the integument
KRT16	E7 (Ker)	757.5	Congenital anomalies of the integument
MDM2	E6 (IMR90)	216.9	Benign neoplasm of skin
MMP1	E7 (Ker)	496	Chronic airway obstruction
MMP1	E7 (Ker)	757.39	Congenital anomalies of the integument
MMP2	E6 (IMR90) E7 (IMR90)	733.9	Other disorders of bone and cartilage
**MSH2**	E6 (IMR90) E7 (IMR90)	**153**	**Malignant neoplasm of colon**
**MSH2**	E6 (IMR90) E7 (IMR90)	**183**	**Malignant neoplasm of ovary**
MSH2	E6 (IMR90) E7 (IMR90)	191.9	Malignant neoplasm of brain
MSH2	E6 (IMR90) E7 (IMR90)	202.8	Other malignant neoplasms of lymphoid and histiocytic tissue
MSH2	E6 (IMR90) E7 (IMR90)	237.7	Neurofibromatosis
MSH2	E6 (IMR90) E7 (IMR90)	706.2	Diseases of sebaceous glands, Sebaceous cyst
MSH2	E6 (IMR90) E7 (IMR90)	709.09	Other disorders of skin and subcutaneous tissue
NDRG1	E6 (IMR90)	356.1	Hereditary and idiopathic peripheral neuropathy
**ODC1**	E6 (IMR90) E7 (IMR90)	**153**	**Malignant neoplasm of colon**
PCNA	E6 (IMR90) E7 (IMR90) E7 (Ker)	-	
PTTG1	E6 (IMR90)	-	
SESN1	E6 (IMR90) E6 (Ker)	-	
**TNFRSF10B**	E6 (IMR90)	**173.9**	**Head and neck squamous carcinoma**
TNFRSF10C	E6 (IMR90)	-	
TP53INP1	E6 (IMR90)	-	
TRIM22	E6 (IMR90)	-	
WEE1	E7 (IMR90) E7 (Ker)	-	

Supplementary Table S15. Tissue specificity analysis of EBV. Genes present and expressed in Burkitt’s lymphoma (Raji cells) and B lymphocyte tissues are shown based on the GNF1H dataset in http://biogps.org with their expression values and their associated diseases.

Gene	Number of probes	Average expression value in Burkitt's lymphoma (Raji	Average expression value in B Lymphocytes	Icd-9 code	Icd-9 description	Is disease among virally-implicated?	Gene expressed in both tissues, single or neither?

		**cells)**					
ACADM	1	2362.00	3038.50	277.85	Other and unspecified disorders of metabolism		both
ACTN4	2	782.75	1091.50	587	Renal sclerosis		both
ADA	3	2392.83	2359.83	277.2	Other disorders of purine and pyrimidine metabolism		both
ADA	3	2392.83	2359.83	279.2	Combined immunity deficiency	*	both
ATP7B	2	990.25	1062.75	275.1	Disorders of copper metabolism		both
BAX	2	244.25	1073.50	153	Malignant neoplasm of colon		both
BAX	2	244.25	1073.50	204	Lymphoid leukemia		both
BCL2	10	328.95	365.45	200	B-cell lymphoma	*	both
BCL3	3	474.67	379.17	200	B-cell lymphoma	*	both
BLNK	1	825.50	5514.00	279	Hypogammaglobulinemia		both
CD46	5	540.40	1038.00	283.11	Hemolytic-uremic syndrome		both
CD82	1	3477.50	1919.00	185	Malignant neoplasm of prostate		both
CDKN2A	3	1443.33	484.00	157.8	Malignant neoplasm of pancreas		both
CDKN2A	3	1443.33	484.00	161.9	Malignant neoplasm of larynx		both
CDKN2A	3	1443.33	484.00	172.9	Malignant melanoma of skin,		both
CDKN2A	3	1443.33	484.00	V84.01	Genetic susceptibility to malignant neoplasm of breast		both
CTSC	1	320.00	10801.00	523.4	Chronic periodontitis		both
CTSC	1	320.00	10801.00	759.89	Other and unspecified congenital anomalies		both
CYP27B1	3	487.33	358.33	268	Vitamin D deficiency		both
DDB2	1	1002.00	3277.50	757.33	Congenital anomalies of the integument		both
DHFR	4	468.75	2149.38	285.9	Other and unspecified anemias		both
EFEMP2	5	530.80	221.60	756.83	Other congenital musculoskeletal anomalies, Ehlers-Danlos syndrome		both
EP300	4	268.88	334.00	153	Malignant neoplasm of colon		both
EP300	4	268.88	334.00	759.89	Other and unspecified congenital anomalies		both
FANCC	2	959.25	533.25	284.09	Fanconi anemia		both
FHL3	1	424.50	216.00	288.4	Diseases of white blood cells, Hemophagocytic syndromes	*	both
GLUD1	2	1593.25	3720.00	251.2	Hypoglycemia, unspecified		both
GLUD1	2	1593.25	3720.00	255.2	Adrenogenital disorders		both
GMPS	1	1779.50	4226.00	208.9	Leukemia of unspecified cell type		both
HBB	3	817.00	515.83	207	Other specified leukemia		both
HBB	3	817.00	515.83	282.49	Hereditary hemolytic anemias, Other thalassemia		both
HBB	3	817.00	515.83	282.6	Hereditary hemolytic anemias, Sickle-cell disease		both
HBB	3	817.00	515.83	282.7	Hereditary hemolytic anemias, Other hemoglobinopathies		both
HBB	3	817.00	515.83	289.7	Other diseases of blood and blood-forming organs,		both

					Methemoglobinemia		
HLA-A	3	22339.83	34010.17	695.1	Erythema multiforme		both
HLA-A	3	22339.83	34010.17	720	Ankylosing spondylitis		both
HLA-A	3	22339.83	34010.17	995.27	Certain adverse effects not elsewhere classified, Other drug allergy		both
HLA-B	4	25292.38	36297.75	695.1	Erythema multiforme		both
HLA-B	4	25292.38	36297.75	720	Ankylosing spondylitis		both
HLA-B	4	25292.38	36297.75	995.27	Certain adverse effects not elsewhere classified, Other drug allergy		both
HRAS	2	628.50	779.00	188.9	Malignant neoplasm of bladder		both
HRAS	2	628.50	779.00	193	Malignant neoplasm of thyroid gland		both
HRAS	2	628.50	779.00	744.9	Congenital anomalies of ear, face, and neck		both
HSPD1	6	4209.25	11484.08	94	Neurosyphilis		both
IL1B	4	617.88	442.38	151.9	Malignant neoplasm of stomach	*	both
IL6	1	486.00	322.50	176.9	Kaposi's sarcoma,		both
IL6	1	486.00	322.50	250	Diabetes mellitus		both
IL6	1	486.00	322.50	555.9	Regional enteritis, Unspecified site		both
IL6	1	486.00	322.50	714	Rheumatoid arthritis and other inflammatory polyarthropathies		both
ITGB2	1	3014.50	4061.00	288.6	Diseases of white blood cells		both
KIT	1	212.00	219.50	186.9	Malignant neoplasm of testis		both
KIT	1	212.00	219.50	207.8	Other specified leukemia		both
KIT	1	212.00	219.50	208.9	Leukemia of unspecified cell type		both
KIT	1	212.00	219.50	238.1	Neoplasm of uncertain behavior of other and unspecified sites and tissues	*	both
KIT	1	212.00	219.50	709.09	Other disorders of skin and subcutaneous tissue		both
KIT	1	212.00	219.50	757.33	Congenital anomalies of the integument		both
LCK	2	310.25	4153.50	279.2	Combined immunity deficiency		both
LDHB	3	11363.17	22011.50	271.3	Disorders of carbohydrate transport and metabolism, Intestinal disaccharidase deficiencies		both
LRPPRC	2	2266.75	4415.75	330.8	Cerebral degenerations usually manifest in childhood		both
MDM2	8	270.25	219.06	216.9	Benign neoplasm of skin		both
MSH2	1	585.50	1684.50	153	Malignant neoplasm of colon		both
MSH2	1	585.50	1684.50	183	Malignant neoplasm of ovary and other uterine adnexa		both
MSH2	1	585.50	1684.50	191.9	Malignant neoplasm of brain,		both
MSH2	1	585.50	1684.50	202.8	Other malignant neoplasms of lymphoid and histiocytic tissue	*	both
MSH2	1	585.50	1684.50	237.7	Neurofibromatosis		both
MSH2	1	585.50	1684.50	706.2	Diseases of sebaceous glands, Sebaceous cyst		both
MSH2	1	585.50	1684.50	709.09	Other disorders of skin and		both

					subcutaneous tissue		
MSH2	1	585.50	1684.50	V84.01	Genetic susceptibility to malignant neoplasm of breast		both
MYC	1	2472.50	5876.50	200.2	Burkitt's lymphoma	*	both
NAP1L1	9	8388.50	8389.72	365.9	Glaucoma		both
NDRG1	1	485.50	279.00	356.1	Peroneal muscular atrophy		both
NFKBIA	1	2194.00	15825.00	757.31	Congenital anomalies of the integument,		both
NME1	1	3645.00	12152.50	194	Malignant neoplasm of other endocrine glands and related structures		both
OPTN	2	297.00	930.25	365.9	Glaucoma		both
PCCB	1	1709.50	3239.00	276.2	Acidosis		both
PEX5	4	1099.88	601.25	277.86	Peroxisomal disorders		both
PLAU	2	581.00	433.75	331	Other cerebral degenerations		both
PRPF8	1	2026.50	6409.50	362.74	Pigmentary retinal dystrophy		both
RB1	3	483.00	552.67	170.9	Malignant neoplasm of bone and articular cartilage		both
RB1	3	483.00	552.67	188.9	Malignant neoplasm of bladder		both
RB1	3	483.00	552.67	190.5	Malignant neoplasm of eye, Retina		both
SERPINA1	8	394.50	1499.38	287.9	Purpura and other hemorrhagic conditions,		both
SERPINA1	8	394.50	1499.38	492.8	Emphysema		both
SFTPB	5	537.60	293.50	515	Postinflammatory pulmonary fibrosis		both
SMARCB1	2	1568.00	1055.75	191.9	Malignant neoplasm of brain		both
SMN1	1	605.00	526.00	335.1	Anterior horn cell disease, Spinal muscular atrophy		both
SRC	8	222.81	316.19	153	Malignant neoplasm of colon		both
TBP	2	628.25	862.50	332	Parkinson's disease		both
TBP	2	628.25	862.50	333.4	Other extrapyramidal disease and abnormal movement disorders, Huntington's chorea		both
TBP	2	628.25	862.50	334.3	Spinocerebellar disease, Other cerebellar ataxia		both
TNFRSF10B	7	505.86	1134.57	173.9	Other malignant neoplasm of skin		both
TNNT3	1	890.50	223.00	728.3	Disorders of muscle, ligament, and fascia		both
TNXB	15	254.57	210.57	756.83	Ehlers-Danlos syndrome		both
TP53	2	574.00	3729.50	147.9	Malignant neoplasm of nasopharynx	*	both
TP53	2	574.00	3729.50	153	Malignant neoplasm of colon		both
TP53	2	574.00	3729.50	157.8	Malignant neoplasm of pancreas		both
TP53	2	574.00	3729.50	170.9	Malignant neoplasm of bone and articular cartilage		both
TP53	2	574.00	3729.50	193	Malignant neoplasm of thyroid gland		both
TP53	2	574.00	3729.50	194	Malignant neoplasm of other endocrine glands		both
TP53	2	574.00	3729.50	211.5	Benign neoplasm of liver and biliary passages	*	both

TP53	2	574.00	3729.50	216.9	Benign neoplasm of skin,		both
TP53	2	574.00	3729.50	239.3	Neoplasms of unspecified nature, Breast	*	both
TP53	2	574.00	3729.50	259.2	Other endocrine disorders, Carcinoid		both
TP53	2	574.00	3729.50	V84.01	Genetic susceptibility to malignant neoplasm of breast		both
TP63	7	559.29	231.64	374.46	Other disorders of eyelids, Blepharophimosis		both
TP63	7	559.29	231.64	755.4	Other congenital anomalies of limbs,		both
TP63	7	559.29	231.64	755.5	Other congenital anomalies of limbs,		both
TP63	7	559.29	231.64	755.67	Other congenital anomalies of limbs,		both
TP63	7	559.29	231.64	757.31	Congenital ectodermal dysplasia		both
TP63	7	559.29	231.64	757.8	Congenital anomalies of the integument		both
TP73	2	342.50	528.25	194	Malignant neoplasm of other endocrine glands		both
TSG101	4	486.50	1033.25	239.3	Neoplasms of unspecified nature, Breast	*	both
TYR	1	774.50	324.50	270.2	Disorders of amino-acid transport and metabolism		both
TYR	1	774.50	324.50	756.89	Other congenital musculoskeletal anomalies		both
VEGFA	7	673.00	1171.43	250	Diabetes mellitus		both
YWHAE	5	630.30	1648.60	758.33	Chromosomal anomalies, Other microdeletions		both
ZAP70	5	857.40	1563.90	757.1	Congenital anomalies of the integument		both
APC	9	189.22	231.61	151.9	Malignant neoplasm of stomach	*	single
APC	9	189.22	231.61	153	Malignant neoplasm of colon		single
APC	9	189.22	231.61	171.9	Malignant neoplasm of connective and other soft tissue		single
APC	9	189.22	231.61	211.3	Benign neoplasm of other parts of digestive system, Colon		single
APC	9	189.22	231.61	211.4	Benign neoplasm of other parts of digestive system, Rectum and anal canal		single
APC	9	189.22	231.61	287.2	Purpura and other hemorrhagic conditions,		single
APC	9	189.22	231.61	425.4	Cardiomyopathy		single
APC	9	189.22	231.61	-	Hepatoblastoma	*	single
APOA1	2	298.75	114.75	272.1	Pure hyperglyceridemia		single
APOA1	2	298.75	114.75	272.5	Disorders of lipoid metabolism		single
APOA1	2	298.75	114.75	277.39	Other amyloidosis		single
APOA1	2	298.75	114.75	281.4	Protein-deficiency anemia		single
APOA1	2	298.75	114.75	371.5	Corneal opacity and other disorders of cornea,		single
EGFR	9	309.83	137.28	162.9	Malignant neoplasm of trachea, bronchus, and lung	*	single
FN1	8	215.13	173.50	183	Malignant neoplasm of ovary and other uterine adnexa		single
FN1	8	215.13	173.50	208.9	Leukemia of unspecified cell type		single
FN1	8	215.13	173.50	569.89	Other disorders of intestine		single

GFAP	6	197.67	201.08	781.99	Symptoms involving nervous and musculoskeletal systems		single
HSPB1	2	49.75	470.50	355.9	Mononeuritis of lower limb		single
HSPB1	2	49.75	470.50	356.1	Peroneal muscular atrophy		single
IGLL1	3	283.67	198.50	279	Disorders involving the immune mechanism		single
ITGA2B	6	211.92	122.92	287.1	Purpura and other hemorrhagic conditions		single
ITGA2B	6	211.92	122.92	287.5	Purpura and other hemorrhagic conditions, Thrombocytopenia		single
NEFL	3	270.83	52.33	356.1	Hereditary and idiopathic peripheral neuropathy		single
PCCA	2	152.75	244.25	276.2	Acidosis		single
PSEN2	6	264.58	189.58	331	Other cerebral degenerations		single
PSEN2	6	264.58	189.58	425.4	Cardiomyopathy		single
RECQL4	1	125.00	380.50	755.64	Other congenital anomalies of limbs		single
RECQL4	1	125.00	380.50	757.33	Congenital anomalies of the integument		single
SLC22A18	2	145.75	321.50	162.9	Malignant neoplasm of trachea, bronchus, and lung	*	single
SLC22A18	2	145.75	321.50	171.9	Malignant neoplasm of connective and other soft tissue		single
SLC22A18	2	145.75	321.50	239.3	Neoplasms of unspecified nature, Breast	*	single
VWF	2	200.50	143.75	286.4	Coagulation defects, von Willebrand's disease		single
ADH1C	1	141.50	59.50				neither
ADIPOQ	1	121.00	43.50				neither
APOH	2	88.00	136.00				neither
CCND1	2	91.75	121.50				neither
CYP11B2	1	124.50	184.50				neither
EFEMP1	2	180.25	133.00				neither
FBLN5	1	110.50	61.00				neither

KLK3	4	155.38	112.63				neither
LAMB1	2	98.00	33.00				neither
MMP2	2	138.75	177.50				neither
PAX6	1	74.50	27.50				neither
PLTP	1	74.00	38.50				neither
TRHR	1	46.00	40.00				neither
NCF1	not on array						neither

Supplementary Table S16. Tissue specificity analysis of HPV16. Genes present and expressed in ovary and testis tissues are shown based on the GNF1H dataset in http://biogps.org with their expression values and their associated diseases.

Gene	Number of probes	Average expression value in ovary	Average expression value in testis	ICD-9 code	ICD-9 description	Is disease among virally-implicated?	Gene expressed in both tissues, single or neither?
ABCB1	1	376.50	307.00	974.7	Poisoning by water, mineral, and uric acid metabolism drugs		both
ADA	3	231.50	307.83	277.2	Other disorders of purine and pyrimidine metabolism		both
ADA	3	231.50	307.83	279.2	Combined immunity deficiency		both
ALOX5AP	1	378.00	560.50	410.9	Acute myocardial infarction		both
ALOX5AP	1	378.00	560.50	434.91			both
ALPL	1	230.00	318.50	275.3	Disorders of phosphorus metabolism		both

ALPL	1	230.00	318.50	520.4	Disorders of tooth development and eruption,		both
APC	9	276.44	248.78	151.9	Malignant neoplasm of stomach		both
APC	9	276.44	248.78	153	Malignant neoplasm of colon		both
APC	9	276.44	248.78	171.9	Malignant neoplasm of connective and other soft tissue		both
APC	9	276.44	248.78	211.3	Benign neoplasm of other parts of digestive system, Colon		both
APC	9	276.44	248.78	211.4	Benign neoplasm of other parts of digestive system, Rectum and anal canal		both
APC	9	276.44	248.78	287.2	Purpura and other hemorrhagic conditions		both
APC	9	276.44	248.78	425.4	Cardiomyopathy		both
APOB	1	289.00	381.00	272	Disorders of lipoid metabolism		both
APOB	1	289.00	381.00	272.5	Lipoprotein deficiencies		both
APP	5	488.90	497.10	255.5	Disorders of adrenal glands		both
APP	5	488.90	497.10	277.39	Other and unspecified disorders of metabolism, Other amyloidosis		both
APP	5	488.90	497.10	295.9	Schizophrenic disorders		both
APP	5	488.90	497.10	331	Other cerebral degenerations		both
BCL2	10	537.85	479.35	200	B-cell lymphoma		both
BRCA1	3	432.67	392.67	158.8	Malignant neoplasm of retroperitoneum and peritoneum		both
BRCA1	3	432.67	392.67	183	Malignant neoplasm of ovary and other uterine adnexa	*	both
BRCA1	3	432.67	392.67	239.3	Neoplasms of unspecified nature, Breast	*	both
CD82	1	379.00	440.50	185	Malignant neoplasm of prostate	*	both
CDK4	5	559.90	492.10	172.9	Malignant melanoma of skin		both
CEBPB	2	1454.25	337.00	253.3	Disorders of the pituitary gland and its hypothalamic control		both
CIITA	3	345.67	334.83	279.2	Combined immunity deficiency		both
CIITA	3	345.67	334.83	340	Multiple sclerosis		both
CIITA	3	345.67	334.83	714	Rheumatoid arthritis and other inflammatory polyarthropathies		both
CREBBP	2	301.00	611.00	368.5	Visual disturbances, Color vision deficiencies		both
CREBBP	2	301.00	611.00	368.54	Visual disturbances, Achromatopsia		both
CREBBP	2	301.00	611.00	759.89	Other and unspecified congenital anomalies		both
CXCR4	5	412.60	332.60	288.09	Diseases of white blood cells, Other neutropenia		both
DDB2	1	320.50	479.00	757.33	Congenital anomalies of the integument		both
EGFR	9	403.39	546.78	162.9	Malignant neoplasm of trachea, bronchus, and lung	*	both
ERBB2	8	306.00	414.38	151.9	Malignant neoplasm of stomach		both
ERBB2	8	306.00	414.38	183	Malignant neoplasm of ovary and other uterine adnexa	*	both
ERBB2	8	306.00	414.38	191.9	Malignant neoplasm of brain		both
ERBB2	8	306.00	414.38	272.2	Disorders of lipoid metabolism, Mixed hyperlipidemia		both
ERBB2	8	306.00	414.38	-	Adenocarcinoma of lung	*	both

FANCC	2	730.50	730.75	284.09	Fanconi anemia		both
FASLG	3	607.00	360.17	710	Diffuse diseases of connective tissue		both
FBLN1	6	579.58	391.50	755.1	Other congenital anomalies of limbs, Syndactyly		both
FLNA	5	1683.40	804.70	378.3	Strabismus and other disorders of binocular eye movements		both
FLNA	5	1683.40	804.70	733.9	Other disorders of bone and cartilage		both
FLNA	5	1683.40	804.70	756	Other congenital musculoskeletal anomalies		both
FLNA	5	1683.40	804.70	757.39	Congenital anomalies of the integument		both
FLNA	5	1683.40	804.70	759.89	Other and unspecified congenital anomalies		both
FN1	8	848.94	912.69	183	Malignant neoplasm of ovary and other uterine adnexa	*	both
FN1	8	848.94	912.69	208.9	Leukemia of unspecified cell type		both
FN1	8	848.94	912.69	569.89	Other disorders of intestine, Other		both
GFAP	6	515.92	302.58	781.99	Symptoms involving nervous and musculoskeletal systems		both
GJA1	1	411.50	374.00	389.9	Hearing loss		both
GJA1	1	411.50	374.00	426.1	Conduction disorders, Atrioventricular block		both
GJA1	1	411.50	374.00	746.7	Hypoplastic left heart syndrome		both
GJA1	1	411.50	374.00	755.1	Syndactyly		both
GJA1	1	411.50	374.00	759.89	Other and unspecified congenital anomalies		both
GSS	7	480.50	694.64	276.2	Disorders of fluid, electrolyte, and acid-base balance, Acidosis		both
GSS	7	480.50	694.64	283.9	Acquired hemolytic anemias,		both
HADHA	5	585.10	507.20	277.85	Disorders of fatty acid oxidation		both
HADHA	5	585.10	507.20	571.8	Chronic liver disease and cirrhosis,		both
HADHA	5	585.10	507.20	642.5	Severe pre-eclampsia		both
HLA-A	3	3113.17	6378.17	695.1	Erythema multiforme		both
HLA-A	3	3113.17	6378.17	720	Ankylosing spondylitis		both
HLA-A	3	3113.17	6378.17	995.27	Certain adverse effects not elsewhere classified, Other drug allergy		both
ICAM1	4	641.88	623.88	84.6	Malaria, Malaria, unspecified		both
IL10	1	204.00	237.00	042	Human immunodeficiency virus [HIV] disease		both
IL10	1	204.00	237.00	714	Rheumatoid arthritis and other inflammatory polyarthropathies		both
IL10	1	204.00	237.00	742.2	Other congenital anomalies of nervous system		both
IL10	1	204.00	237.00	996.85	Complications peculiar to certain specified procedures, Bone marrow		both
IL1B	4	372.25	359.75	151.9	Malignant neoplasm of stomach		both
IL6	1	443.00	299.50	176.9	Kaposi's sarcoma		both
IL6	1	443.00	299.50	250	Diabetes mellitus		both
IL6	1	443.00	299.50	555.9	Regional enteritis, Unspecified site		both
IL6	1	443.00	299.50	714	Rheumatoid arthritis and other inflammatory polyarthropathies		both

IRF1	1	669.50	929.00	151.9	Malignant neoplasm of stomach		both
IRF1	1	669.50	929.00	162.9	Malignant neoplasm of trachea, bronchus, and lung	*	both
IRF1	1	669.50	929.00	205.9	Myeloid leukemia		both
IRF1	1	669.50	929.00	281.9	Other deficiency anemias		both
IRF1	1	669.50	929.00	742.59	Other congenital anomalies of nervous system		both
LMO2	1	408.00	508.50	208.9	Leukemia of unspecified cell type		both
MAN2B1	1	377.50	518.50	271.8	Disorders of carbohydrate transport and metabolism		both
MDM2	8	294.00	289.44	216.9	Benign neoplasm of skin		both
MEFV	4	467.38	274.63	277.31	Familial Mediterranean fever		both
MMP2	2	1146.50	1142.50	733.9	Other disorders of bone and cartilage		both
MYC	1	248.00	306.50	200.2	Burkitt's lymphoma		both
NAT1	1	335.00	318.50	333	Other extrapyramidal disease and abnormal movement disorders		both
NDRG1	1	2543.00	1941.00	356.1	Peroneal muscular atrophy		both
NUMA1	6	249.17	1143.83	208.9	Leukemia of unspecified cell type		both
ODC1	2	851.50	2867.50	153	Malignant neoplasm of colon		both
PLAU	2	223.25	222.25	331	Other cerebral degenerations		both
PRPH	6	362.75	643.92	335.2	Anterior horn cell disease, Motor neuron disease		both
PRPH	6	362.75	643.92	362.16	Other retinal disorders, Retinal neovascularization		both
PRPH	6	362.75	643.92	362.7	Other retinal disorders, Hereditary retinal dystrophies		both
PRPH	6	362.75	643.92	362.74	Other retinal disorders, Pigmentary retinal dystrophy		both
PYGM	3	493.00	705.83	271	Disorders of carbohydrate transport and metabolism		both
SQSTM1	5	421.20	500.90	731	Osteitis deformans and osteopathies		both
TBP	2	782.50	890.25	332	Parkinson's disease		both
TBP	2	782.50	890.25	333.4	Other extrapyramidal disease and abnormal movement disorders, Huntington's chorea		both
TBP	2	782.50	890.25	334.3	Spinocerebellar disease, Other cerebellar ataxia		both
TF	9	312.61	530.83	273.8	Disorders of plasma protein metabolism,		both
TF	9	312.61	530.83	285.9	Other and unspecified anemias,		both
TNF	1	418.50	311.00	84.6	Malaria		both
TNF	1	418.50	311.00	294.8	Persistent mental disorders due to conditions		both
TNF	1	418.50	311.00	346.9	Migraine		both
TNF	1	418.50	311.00	493.9	Asthma		both
TNF	1	418.50	311.00	995.91	Certain adverse effects not elsewhere classified, Sepsis		both
TNFRSF10B	7	233.79	284.00	173.9	Other malignant neoplasm of skin	*	both
TNFRSF1A	2	805.25	493.00	277.3	Other and unspecified disorders of metabolism, Amyloidosis		both
TP53	2	278.75	331.25	147.9	Malignant neoplasm of nasopharynx		both

TP53	2	278.75	331.25	153	Malignant neoplasm of colon	*	both
TP53	2	278.75	331.25	157.8	Malignant neoplasm of pancreas		both
TP53	2	278.75	331.25	170.9	Malignant neoplasm of bone and articular cartilage		both
TP53	2	278.75	331.25	193	Malignant neoplasm of thyroid gland		both
TP53	2	278.75	331.25	194	Malignant neoplasm of other endocrine glands		both
TP53	2	278.75	331.25	211.5	Benign neoplasm of liver and biliary passages		both
TP53	2	278.75	331.25	216.9	Benign neoplasm of skin		both
TP53	2	278.75	331.25	239.3	Neoplasms of unspecified nature, Breast	*	both
TP53	2	278.75	331.25	259.2	Other endocrine disorders, Carcinoid syndrome		both
TP53	2	278.75	331.25	V84.01	Genetic susceptibility to malignant neoplasm of breast		both
TP63	7	268.50	318.36	374.46	Other disorders of eyelids, Blepharophimosis		both
TP63	7	268.50	318.36	755.4	Other congenital anomalies of limbs,		both
TP63	7	268.50	318.36	755.5	Other congenital anomalies of limbs,		both
TP63	7	268.50	318.36	755.67	Other congenital anomalies of limbs, Anomalies of foot		both
TP63	7	268.50	318.36	757.31	Congenital ectodermal dysplasia		both
TP63	7	268.50	318.36	757.8	Congenital anomalies of the integument,		both
TP73	2	698.75	464.00	194	Malignant neoplasm of other endocrine glands and related structures		both
TSHB	1	724.50	528.00	244.9	Acquired hypothyroidism		both
UBE3A	6	287.08	247.33	759.89	Other and unspecified congenital anomalies		both
CASP8	2	242.25	177.75	162.9	Malignant neoplasm of trachea, bronchus, and lung	*	single
CASP8	2	242.25	177.75	211.5	Benign neoplasm of liver and biliary passages		single
CASP8	2	242.25	177.75	239.3	Neoplasms of unspecified nature, Breast	*	single
CASP8	2	242.25	177.75	279.4	Disorders involving the immune mechanism, Autoimmune disease		single
CEBPA	1	190.00	334.00	208.9	Leukemia of unspecified cell type		single
EP300	4	151.25	226.13	153	Malignant neoplasm of colon		single
EP300	4	151.25	226.13	759.89	Other and unspecified congenital anomalies		single
F9	1	274.50	169.00	193	Malignant neoplasm of thyroid gland		single
F9	1	274.50	169.00	286	Coagulation defects		single
F9	1	274.50	169.00	995.2	Adverse effect of drug, medicinal and biological substance (due) to correct medicinal substance properly administered		single
GNRHR	5	240.00	152.00	253.4	Disorders of the pituitary gland and its hypothalamic control		single
GNRHR	5	240.00	152.00	257.2	Testicular dysfunction		single
MYH7	2	163.50	604.75	359	Muscular dystrophies		single
MYH7	2	163.50	604.75	359.9	Muscular dystrophies		single
MYH7	2	163.50	604.75	425.4	Cardiomyopathy		single

OPRM1	9	275.17	198.72	345.9	Epilepsy and recurrent seizures		single
OPRM1	9	275.17	198.72	709	Other disorders of skin and subcutaneous tissue		single
RB1	3	218.17	183.67	170.9	Malignant neoplasm of bone and articular cartilage		single
RB1	3	218.17	183.67	188.9	Malignant neoplasm of bladder	*	single
RB1	3	218.17	183.67	190.5	Malignant neoplasm of eye, Retina		single
RBL2	3	177.33	590.83	331.4	Other cerebral degenerations, Obstructive hydrocephalus		single
TSC2	3	137.83	235.67	518.89	Other diseases of lung		single
TSC2	3	137.83	235.67	759.5	Other and unspecified congenital anomalies, Tuberous sclerosis		single
APOA2	2	106.00	124.00				neither
APOH	2	132.25	62.75				neither
BAX	2	145.00	114.75				neither
CCL5	2	175.25	172.25				neither
CETP	1	37.00	56.00				neither
DHFR	4	154.75	158.00				neither
EDN1	1	68.50	126.50				neither
HOXD13	2	115.00	105.50				neither
KRT16	1	14.00	83.50				neither
MMP1	1	39.50	7.00				neither

MSH2	1	108.50	83.50	neither
PPARG	1	130.00	45.00	neither
SELE	1	101.00	112.00	neither
SPINK1	1	52.50	137.50	neither
TERT	1	146.50	177.00	neither
TGM1	1	32.50	50.50	Neither

Supplementary Figure S11. Degree of viral targets compared to random targets.

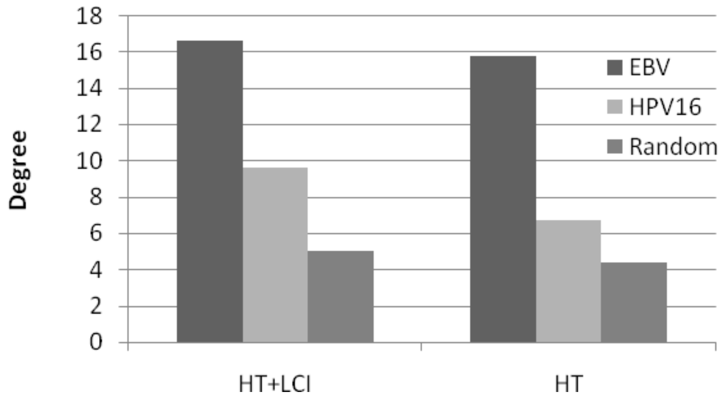

Supplementary Figure S12. Average shortest path between viral targets and randomized genes associated with virally implicated diseases.

a **b**

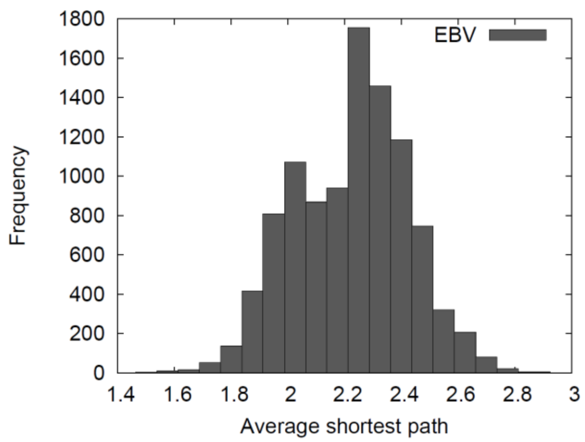

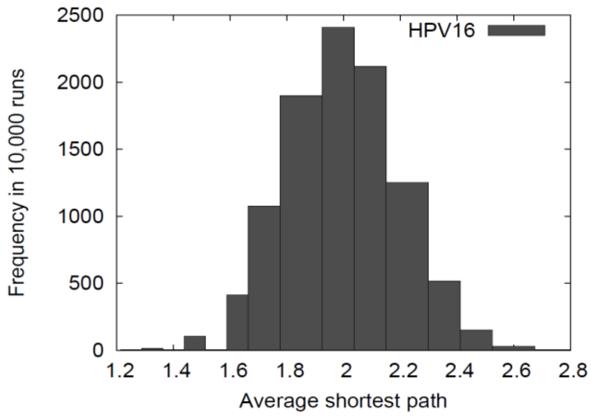

Supplementary Table S17. 38 OMIM diseases that have significant prevalence among HPV patients.

ICD-9 code	ICD-9 description	Patients with disease among HPV patients	Patients with disease among ALL patients	RR	Lower bound	Upper bound	RR (%99 CI)
781	Other diseases due to viruses and Chlamydiae, Viral warts	1630	1630	550.82	516.77	587.10	550.82
6424	Hypertension complicating pregnancy, childbirth.	1	3	183.61	13.97	2413.47	183.61
6238	Noninflammatory disorders of vagina	560	8203	37.60	33.72	41.93	37.60
36212	Other retinal disorders, Exudative retinopathy	1	27	20.40	1.55	268.16	20.40
1905	Malignant neoplasm of eye, Retina	1	35	15.74	1.20	206.86	15.74
2711	Disorders of carbohydrate transport and metabolism, Galactosemia	1	36	15.30	1.16	201.12	15.30
182	Malignant neoplasm of body of uterus	1324	55419	13.16	12.26	14.12	13.16
7528	Congenital anomalies of genital organs	6	260	12.71	4.44	36.38	12.71
2814	Protein-deficiency anemia	4	273	8.07	2.23	29.26	8.07
1991	Malignant neoplasm without specification of site	304	26028	6.43	5.55	7.46	6.43
**1588**	**Malignant neoplasm of peritoneum**	**15**	**1329**	**6.22**	**3.20**	**12.09**	**6.22**
218	Uterine leiomyoma	755	68405	6.08	5.54	6.68	6.08
2693	Mineral deficiency, not elsewhere classified	4	438	5.03	1.39	18.24	5.03
**183**	**Malignant neoplasm of ovary and other uterine adnexa**	**275**	**30275**	**5.00**	**4.28**	**5.84**	**5.00**
2398	Neoplasms of unspecified nature	12	1381	4.79	2.28	10.07	4.79
6927	Contact dermatitis and other eczema, Due to solar radiation	4	495	4.45	1.23	16.14	4.45
330	Cerebral degenerations usually	3	372	4.44	1.00	19.66	4.44

	manifest in childhood						
2698	Other nutritional deficiencies	7	912	4.23	1.60	11.19	4.23
4571	Noninfectious disorders of lymphatic channels	65	8650	4.14	3.01	5.70	4.14
171	Malignant neoplasm of connective and other soft tissue	32	4661	3.78	2.40	5.96	3.78
**284**	**Aplastic anemia and other bone marrow failure syndromes**	**330**	**49062**	**3.70**	**3.22**	**4.27**	**3.70**
**216**	**Benign neoplasm of skin**	**32**	**5480**	**3.22**	**2.04**	**5.07**	**3.22**
7011	Keratoderma	22	3934	3.08	1.78	5.33	3.08
704	Diseases of hair and hair follicles	10	1911	2.88	1.28	6.51	2.88
2880	Diseases of white blood cells, Neutropenia	302	60248	2.76	2.38	3.20	2.76
448	Disease of capillaries	17	3420	2.74	1.47	5.11	2.74
**188**	**Malignant neoplasm of bladder**	**137**	**27706**	**2.72**	**2.19**	**3.39**	**2.72**
593	Other disorders of kidney and ureter	911	184443	2.72	2.50	2.96	2.72
5997	Other disorders of urethra and urinary tract, Hematuria	652	134644	2.67	2.41	2.95	2.67
2362	Neoplasm of uncertain behavior of genitourinary organs, Ovary	15	3212	2.57	1.32	5.00	2.57
170	Malignant neoplasm of bone and articular cartilage	13	2808	2.55	1.25	5.21	2.55
2863	Coagulation defects, Congenital deficiency of other clotting factors	9	2028	2.44	1.04	5.77	2.44
2752	Disorders of mineral metabolism	128	29063	2.43	1.93	3.05	2.43
5698	Other disorders of intestine	232	57630	2.22	1.87	2.63	2.22
**173**	**Other malignant neoplasm of skin**	**81**	**20316**	**2.20**	**1.65**	**2.92**	**2.20**
586	Renal failure, unspecified	183	47789	2.11	1.74	2.55	2.11
453	Other venous embolism and thrombosis	552	147560	2.06	1.85	2.30	2.06
**7062**	**Diseases of sebaceous glands, Sebaceous cyst**	**22**	**5884**	**2.06**	**1.19**	**3.57**	**2.06**

References

1. Lamesch P, Li N, Milstein S, Fan C, Hao T, et al. (2007) hORFeome v3.1: a resource of human open reading frames representing over 10,000 human genes. Genomics 89: 307-315.
2. Rual JF, Hirozane-Kishikawa T, Hao T, Bertin N, Li S, et al. (2004) Human ORFeome version 1.1: a platform for reverse proteomics. Genome Res 14: 2128-2135.
3. Rual JF, Venkatesan K, Hao T, Hirozane-Kishikawa T, Dricot A, et al. (2005) Towards a proteome-scale map of the human protein-protein interaction network. Nature 437: 1173-1178.
4. Dreze M, Monachello D, Lurin C, Cusick ME, Hill DE, et al. (2009) High-quality binary interactome mapping. Methods Enzymol 470: 281-315.
5. Calderwood MA, Venkatesan K, Xing L, Chase MR, Vazquez A, et al. (2007) Epstein-Barr virus and virus human protein interaction maps. Proc Natl Acad Sci USA 104: 7606-7611.

6. Chatr-aryamontri A, Ceol A, Peluso D, Nardozza A, Panni S, et al. (2009) VirusMINT: a viral protein interaction database. Nucleic Acids Res 37: D669-673.
7. Huh KW, DeMasi J, Ogawa H, Nakatani Y, Howley PM, et al. (2005) Association of the human papillomavirus type 16 E7 oncoprotein with the 600-kDa retinoblastoma protein-associated factor, p600. Proc Natl Acad Sci USA 102: 11492-11497.
8. Aranda B, Achuthan P, Alam-Faruque Y, Armean I, Bridge A, et al. (2010) The IntAct molecular interaction database in 2010. Nucleic Acids Res 38: D525-531.
9. Ceol A, Chatr Aryamontri A, Licata L, Peluso D, Briganti L, et al. (2010) MINT, the molecular interaction database: 2009 update. Nucleic Acids Res 38: D532-539.
10. Stelzl U, Worm U, Lalowski M, Haenig C, Brembeck FH, et al. (2005) A human protein-protein interaction network: a resource for annotating the proteome. Cell 122: 957-968.
11. Venkatesan K, Rual JF, Vazquez A, Stelzl U, Lemmens I, et al. (2009) An empirical framework for binary interactome mapping. Nat Methods 6: 83-90.
12. Matys V, Kel-Margoulis OV, Fricke E, Liebich I, Land S, et al. (2006) TRANSFAC and its module TRANSCompel: transcriptional gene regulation in eukaryotes. Nucleic Acids Res 34: D108-110.
13. Mani KM, Lefebvre C, Wang K, Lim WK, Basso K, et al. (2008) A systems biology approach to prediction of oncogenes and molecular perturbation targets in B-cell lymphomas. Mol Syst Biol 4: 169.
14. Lee DS, Park J, Kay KA, Christakis NA, Oltvai ZN, et al. (2008) The implications of human metabolic network topology for disease comorbidity. Proc Natl Acad Sci USA 105: 9880-9885.
15. Kanehisa M, Goto S, Furumichi M, Tanabe M, Hirakawa M (2010) KEGG for representation and analysis of molecular networks involving diseases and drugs. Nucleic Acids Res 38: D355-360.
16. Schellenberger J, Park JO, Conrad TM, Palsson BO (2010) BiGG: a Biochemical Genetic and Genomic knowledgebase of large scale metabolic reconstructions. BMC Bioinformatics 11: 213.
17. Burgard AP, Nikolaev EV, Schilling CH, Maranas CD (2004) Flux coupling analysis of genome-scale metabolic network reconstructions. Genome Res 14: 301-312.
18. Duarte NC, Becker SA, Jamshidi N, Thiele I, Mo ML, et al. (2007) Global reconstruction of the human metabolic network based on genomic and bibliomic data. Proc Natl Acad Sci USA 104: 1777-1782.
19. Amberger J, Bocchini CA, Scott AF, Hamosh A (2009) McKusick's Online Mendelian Inheritance in Man (OMIM). Nucleic Acids Res 37: D793-796.
20. Goh KI, Cusick ME, Valle D, Childs B, Vidal M, et al. (2007) The human disease network. Proc Natl Acad Sci USA 104: 8685-8690.
21. Valiant LG (1979) The complexity of enumeration and reliability problems. Siam J Computing 8: 410-421.
22. Roberts B (2007) Estimating the number of s-t paths in a graph. J Graph Algorithms Applications 11: 195-214.
23. Barabási AL, Oltvai ZN (2004) Network biology: understanding the cell's functional organization. Nat Rev Genet 5: 101-113.
24. Uetz P, Rajagopala SV, Dong YA, Haas J (2004) From ORFeomes to protein interaction maps in viruses. Genome Res 14: 2029-2033.
25. Gerlee P, Lizana L, Sneppen K (2009) Pathway identification by network pruning in the metabolic network of Escherichia coli. Bioinformatics 25: 3282-3288.
26. Gentleman RC, Carey VJ, Bates DM, Bolstad B, Dettling M, et al. (2004) Bioconductor: open software development for computational biology and bioinformatics. Genome Biol 5: R80.
27. Barrett T, Troup DB, Wilhite SE, Ledoux P, Evangelista C, et al. (2010) NCBI GEO: archive for functional genomics data sets--10 years on. Nucleic Acids Res.

28. McLaughlin-Drubin ME, Munger K (2009) Oncogenic activities of human papillomaviruses. Virus Res 143: 195-208.
29. Schiffman M, Castle PE, Jeronimo J, Rodriguez AC, Wacholder S (2007) Human papillomavirus and cervical cancer. Lancet 370: 890-907.
30. Scheffner M, Munger K, Byrne JC, Howley PM (1991) The state of the p53 and retinoblastoma genes in human cervical carcinoma cell lines. Proc Natl Acad Sci USA 88: 5523-5527.
31. Park J, Lee DS, Christakis NA, Barabasi AL (2009) The impact of cellular networks on disease comorbidity. Mol Syst Biol 5: 262.
32. Hidalgo CA, Blumm N, Barabasi AL, Christakis NA (2009) A dynamic network approach for the study of human phenotypes. PLoS Comput Biol 5: e1000353.
33. Katz D, Baptista J, Azen SP, Pike MC (1978) Obtaining confidence intervals for the risk ratio in cohort studies. Biometrics 34: 469-474.
34. Kieff E, Rickinson AB (2001) Epstein-Barr Virus and its replication. In: Knipe DM, Howley PM, editors. Fundamental Virology. Philadelphia: Lippincott Williams & Wilkins. pp. 1185-1247.
35. Navlakha S, Kingsford C (2010) The power of protein interaction networks for associating genes with diseases. Bioinformatics 26: 1057-1063.
36. Vanunu O, Magger O, Ruppin E, Shlomi T, Sharan R (2010) Associating genes and protein complexes with disease via network propagation. PLoS Comput Biol 6: e1000641.
37. Tategu M, Arauchi T, Tanaka R, Nakagawa H, Yoshida K (2007) Systems biology-based identification of crosstalk between E2F transcription factors and the Fanconi anemia pathway. Gene Regul Syst Bio 1: 1-8.
38. Su AI, Cooke MP, Ching KA, Hakak Y, Walker JR, et al. (2002) Large-scale analysis of the human and mouse transcriptomes. Proc Natl Acad Sci USA 99: 4465-4470.
39. Niedobitek G, Agathanggelou A, Rowe M, Jones EL, Jones DB, et al. (1995) Heterogeneous expression of Epstein-Barr virus latent proteins in endemic Burkitt's lymphoma. Blood 86: 659-665.
40. Kelly GL, Milner AE, Baldwin GS, Bell AI, Rickinson AB (2006) Three restricted forms of Epstein-Barr virus latency counteracting apoptosis in c-myc-expressing Burkitt lymphoma cells. Proc Natl Acad Sci USA 103: 14935-14940.
41. Yano T, Sander CA, Andrade RE, Gauwerky CE, Croce CM, et al. (1993) Molecular analysis of the BCL-3 locus at chromosome 17q22 in B-cell neoplasms. Blood 82: 1813-1819.
42. Canoz O, Rassidakis GZ, Admirand JH, Medeiros LJ (2004) Immunohistochemical detection of BCL-3 in lymphoid neoplasms: a survey of 353 cases. Mod Pathol 17: 911-917.
43. Flavell KJ, Murray PG (2000) Hodgkin's disease and the Epstein-Barr virus. Mol Pathol 53: 262-269.
44. Young LS, Rickinson AB (2004) Epstein-Barr virus: 40 years on. Nat Rev Cancer 4: 757-768.
45. Sugawara Y, Mizugaki Y, Uchida T, Torii T, Imai S, et al. (1999) Detection of Epstein-Barr virus (EBV) in hepatocellular carcinoma tissue: a novel EBV latency characterized by the absence of EBV-encoded small RNA expression. Virology 256: 196-202.
46. Li W, Wu BA, Zeng YM, Chen GC, Li XX, et al. (2004) Epstein-Barr virus in hepatocellular carcinogenesis. World J Gastroenterol 10: 3409-3413.
47. Castro CY, Ostrowski ML, Barrios R, Green LK, Popper HH, et al. (2001) Relationship between Epstein-Barr virus and lymphoepithelioma-like carcinoma of the lung: a clinicopathologic study of 6 cases and review of the literature. Hum Pathol 32: 863-872.
48. Ngan RK, Yip TT, Cheng WW, Chan JK, Cho WC, et al. (2002) Circulating Epstein-Barr virus DNA in serum of patients with lymphoepithelioma-like carcinoma of the lung: a potential surrogate marker for monitoring disease. Clin Cancer Res 8: 986-994.
49. Dyer MD, Murali TM, Sobral BW (2008) The landscape of human proteins interacting with viruses and other pathogens. PLoS Pathog 4: e32.

50. Arvin A, Campadelli-Fiume G, Mocarski E, Moore PS, Roizman B, et al. (2007) Human Herpesviruses: Biology, Therapy, and Immunoprophylaxis. Cambridge UK: Cambridge University Press.
51. Slebos RJ, Yi Y, Ely K, Carter J, Evjen A, et al. (2006) Gene expression differences associated with human papillomavirus status in head and neck squamous cell carcinoma. Clin Cancer Res 12: 701-709.
